# Revealing the Presence of a Symbolic Sequence Representing Multiple Nucleotides Based on K-Means Clustering of Oligonucleotides

**DOI:** 10.3390/molecules24020348

**Published:** 2019-01-18

**Authors:** Byoungsang Lee, So Yeon Ahn, Charles Park, James J. Moon, Jung Heon Lee, Dan Luo, Soong Ho Um, Seung Won Shin

**Affiliations:** 1 School of Advanced Materials Science and Engineering, Sungkyunkwan University, Suwon 16419, Gyeonggi-do, Korea; carryer123@skku.edu (B.L.); jhlee7@skku.edu (J.H.L.); 2 School of Chemical Engineering, Sungkyunkwan University, Suwon 16419, Gyeonggi-do, Korea; meliss100@skku.edu (S.Y.A.); sh.um@skku.edu (S.H.U.); 3 Biointerfaces Institute, University of Michigan, Ann Arbor, MI 48109, USA; pcharlie@umich.edu (C.P.); moonjj@med.umich.edu (J.J.M.); 4 Department of Biomedical Engineering, University of Michigan, Ann Arbor, MI 48109, USA; 5 Department of Pharmaceutical Sciences, University of Michigan, Ann Arbor, MI 48109, USA; 6 SKKU Advanced Institute of Nanotechnology (SAINT), Sungkyunkwan University, Suwon 16419, Gyeonggi-do, Korea; 7 Department of Biological and Environmental Engineering, Cornell University, Ithaca, NY 14850, USA; dl97@cornell.edu

**Keywords:** representative nucleotide, hybridization profile, K-means clustering, multiple equilibria, sociogram

## Abstract

In biological systems, a few sequence differences diversify the hybridization profile of nucleotides and enable the quantitative control of cellular metabolism in a cooperative manner. In this respect, the information required for a better understanding may not be in each nucleotide sequence, but representative information contained among them. Existing methodologies for nucleotide sequence design have been optimized to track the function of the genetic molecule and predict interaction with others. However, there has been no attempt to extract new sequence information to represent their inheritance function. Here, we tried to conceptually reveal the presence of a representative sequence from groups of nucleotides. The combined application of the K-means clustering algorithm and the social network analysis theorem enabled the effective calculation of the representative sequence. First, a “common sequence” is made that has the highest hybridization property to analog sequences. Next, the sequence complementary to the common sequence is designated as a ‘representative sequence’. Based on this, we obtained a representative sequence from multiple analog sequences that are 8–10-bases long. Their hybridization was empirically tested, which confirmed that the common sequence had the highest hybridization tendency, and the representative sequence better alignment with the analogs compared to a mere complementary.

## 1. Introduction

In living organisms, the sequence of nucleotides enables the design of biological activities and also conveys essential heritage information to descendants. As is well-known, one of the most important features of nucleotides is their ability to interact with each other in a complementary manner, a process called hybridization. The hybridization of nucleotides in perfect complementation is not only stable but highly selective. This complex and delicate reaction can be interpreted with simple thermodynamic principles. The parameters to calculate the thermodynamic properties of the nucleotides involve enthalpy, entropy, and Gibbs free energy. For instance, the melting temperature (T_m_) of nucleotides is precisely described by the Van’t Hoff equation [1,2], and the hybridization interaction between nucleotides is well-described by the nearest-neighbor model in a sequence-specific manner [3,4]. Moreover, computational tools have enabled the prediction of detailed kinetics of hybridization and have contributed to the development of nucleic acid engineering [5,6,7,8,9].

If there are a few mismatches in a complementary sequence, hybridization becomes weaker and less stable. Even though the hybridization is relatively weak, the presence of mismatches in nucleotides often provides the potential to fine-control reactivity. In the case of RNA interference, when the interfering RNA fragment, such as siRNA or microRNA, is the perfect complement of the target region of mRNA, hybridization at the complementary site leads to endonucleolytic cleavage and degradation of mRNA [10,11,12]. However, when there are mismatches, the complex forms a bulged structure. This mismatched bulge prevents the complex from degrading but makes it possible to delicately regulate the efficiency of protein expression [12,13,14]. A group of microRNAs sharing sequence similarity is categorized as a microRNA family. Their sequences are diversified by only a few bases, but they manipulate thousands of mRNAs in a cooperative and harmonized manner [15,16,17]. Thus, the diversity of sequences effectively enhances the functionality of nucleotides. In other words, every nucleotide has its own hybridization profile consistent with its perfect complementary and reliable mismatches.

What we should consider is, however, that the diversity of sequence information is better for functionality but also might obscure the information itself. Reduction and concentration on the diversified information in the groups of nucleotides may contribute to a better understanding. A number of methodologies have been developed for nucleotide sequence design so far [18,19,20,21]. The existing sequence design methods are ultimately aimed at inducing precise and specific binding with the target site and suppressing unwanted reactions. They provide useful information about biomolecular interactions. However, no attempt has been made to synthesize a hypothetical sequence that plays a representative role for several target nucleotides, and to confirm their functionality. In this study, we tried to reveal the existence of a representative sequence among multiple oligonucleotides through simple calculations of strand-to-strand interaction. As study models, 8–10-based random nucleotides (Origin) and their analogs (Mutants) were randomly fabricated. We assumed that the mutants were the facets of the diversified form of the origin. The procedure for obtaining the representative sequence from the analogs is as follows. Based on the fact that the most basic functionality of a nucleic acid sequence is hybridization with a complementary sequence, the sequence showing the highest hybridization yields to the analogs was searched, and named the ‘Common complement sequence’ (CS). Within the environment where all of the analogs were present at their respective concentrations, the CS with the highest hybridization yield was determined by the sequences of the analogs and their relative concentrations. The representative sequence (RS) was synthesized from the complementary of the CS. The concentration of the RS was determined so that the hybridization yield of the CS was the same as both of the analogs and the RS. For calculating the thermodynamics of the CS and RS, a nearest-neighbor model and multiple reaction equilibrium were used [3,4].

Through this simple procedure, we confirmed that there is a symbolic sequence that can represent the nucleotides and indicate that the information of the nucleotides can be concentrated. This result may affect the design and detection of target sequences and widen the vision of our understanding of cell biology.

## 2. Results

### 2.1. Mapping Nucleotides according to Sequence Similarity

A variety of methods have been used to identify the network of arbitrary elements [22]. Hamming distance or edit distance is used to measure the relative distance for various kinds of sequence information, including nucleic acid sequences. Hamming distances used as representative indices define the distance between sequences by quantifying the differences. Also, there are algorithms for the efficient alignment of randomly given strings. The most representative examples are the Needleman—Wunsch algorithm and the Smith—Waterman algorithm [23,24]. Each algorithm generates a global and local alignment of the strings, respectively. Both algorithms consider the alignment depending on the match, mismatch, and gap between the strings. The reward or penalty for each match, mismatch, and gap can be reflected at the user’s convenience. In the case of the previously developed miRanda algorithm, the nearest-neighbor parameters are applied to each reward and penalty value to obtain a result in accordance with the hybridization thermodynamic principle [21]. The distance between the sequences can also be determined by measuring the hybridization profile between sequences. We applied the sociogram to effectively show the relation between the sequence and the hybridization profile.

**Figure 1:**
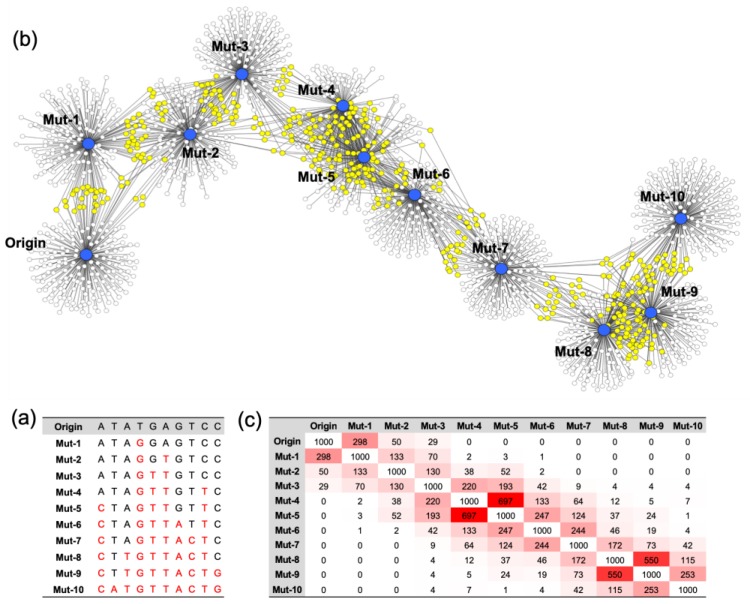
The sequence information and the sociogram of the origin and its mutants. (**a**) The sequences of the mutants were randomly generated by changing the bases of the origin in a cumulative manner. Mutants were created, and the number of each is noted after “Mut-.” (**b**) The origin and the mutants are marked as nodes (blue circles). The top 100 rated complementary sequences (white circles) with the lowest Gibbs free energy were linked with their complements. The complementary sequences have more than two linkages, and are marked as yellow circles. (**c**) For measuring the number of shared complementary sequences (Closeness), 1000 sequences with the lowest Gibbs free energy were used. The closeness of sequences is noted in the table. The cells with a higher number of sequences are marked with a reddish color.

A model nucleotide sequence of 10 random bases (Origin) was generated, and the analog sequences (Mutants) were synthesized by changing the base of the origin in a cumulative manner. Mut-1 was generated by a single base random mutation of the origin, and the mutated base was transferred to the next mutant, Mut-2. Thus, Mut-2 possessed two mismatched sequences from the origin, one of Mut-1 and one of its own. In this way, a total of 10 mutants was generated. When the mutation number becomes higher, the sequence difference between the origin and the mutant is greater. The sequence information of the origin and mutants is noted in Figure 1a. The Gibbs free energy of all the possible complementary strands against the origin and mutants was calculated. Since the sequence consisted of 10 bases, a total of 4^10^ complementary sequences was present. Among all the possible complementary strands, 100 sequences with the lowest Gibbs free energy were selected and connected to each origin or mutant to draw a nondirectional sociogram (Figure 1b). It was shown that the greater the accumulation of mutations in the model nucleotide sequence, the lower the number of shared complementary sequences. The origin shares most of the complementary sequences with one base mismatched nucleotide, Mut-1. Also, the mismatched nucleotides shared most of the complementary sequences with their most similar analogs. This network of the origin and the mutants with the top 1000 rated complementary sequences was quantified, and is presented in Figure 1c. A higher number of shared complementary sequences is indicated with a reddish color. It was clear that every nucleotide had the highest connection with those most similar to it. Also, this result indicates that it is possible to map the nucleotides based on their hybridization profiles.

### 2.2. Generation of an RS from Two Analogs

**Figure 2:**
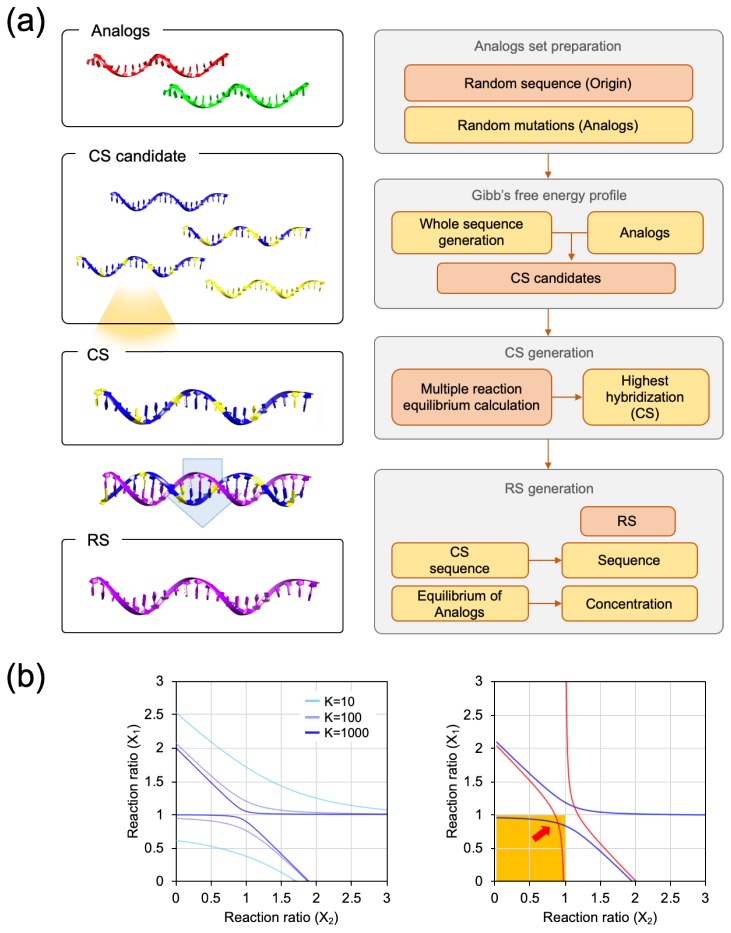
(**a)** The flowchart for calculating the representative sequence (RS) from two analogs. In the complementary validation process, the modified nearest-neighbor model was used. The RS was obtained from the top-rated common complement sequence (CS), and the concentration was adjusted to create an equal amount of hybridization between the analogs. (**b)** The hyperbola graph was obtained from a multiple reaction equilibrium constant calculation, and the hyperbola graph approaches the (1,1) coordinate with the increment in the reaction constant (K). The cross-point of two hyperbola indicates the equilibrium state of the reaction. Reasonable values that were lower than (1,1) were chosen for the summation of the hybridization yield.

Two analogs having mismatches were used as a model to prove the presence of the RS. The calculation procedure is briefly described below and presented in Figure 2a. First, in the procedure for the Gibbs free energy calculation, the nearest-neighbor model was used with some modifications. In the general usage, the nearest-neighbor parameter of the nucleic acid duplex and the terminal base pairs parameters should be included to calculate the enthalpy and entropy of hybridization. Here, we considered the nearest-neighbor parameter in the complementary base pairing for a facile calculation. The nearest-neighbor parameters were referenced from a previous study [4]. The details of the calculation procedure are presented in Figure S1. After the calculation, the Gibbs free energy values of each complementary strand against two analogs were added, and 1000 Strands with the lowest Gibbs free energy were selected as the CS candidates for the next equilibria calculation step. Since the Gibbs free energy values between the analog and the CS candidates were calculated for a single reaction condition, we should convert the Gibbs free energy values to the reaction constants in a multiple reaction, which contains both of the analogs and the CS candidate in a single reaction. The sum of the hybridization yield of the two analogs indicated the involvement of the amount of the CS candidate in the hybridization. Then, the CS was selected from the CS candidates with the highest sum of hybridization yield. The details of the process of the multiple reaction equilibrium are provided in the Figure S2. The formula of the reaction constant (K) was plotted in hyperbolic graphs; as shown in Figure 2b, the hyperbolic graph becomes stiffer with the increment of K. Additionally, the points of intersection in a reasonable range represent the multiple reaction equilibrium state (x). Since one unit of Analogs was involved in the reaction, the (1,1) coordinate indicates perfect hybridization for both Analog 1 and Analog 2. Thus, the reasonable range is the area marked in yellow in Figure 2b. From the CS, an RS and its concentration were calculated. The details of the process for the calculation of the concentration can be found in Figure S3.

**Figure 3:**
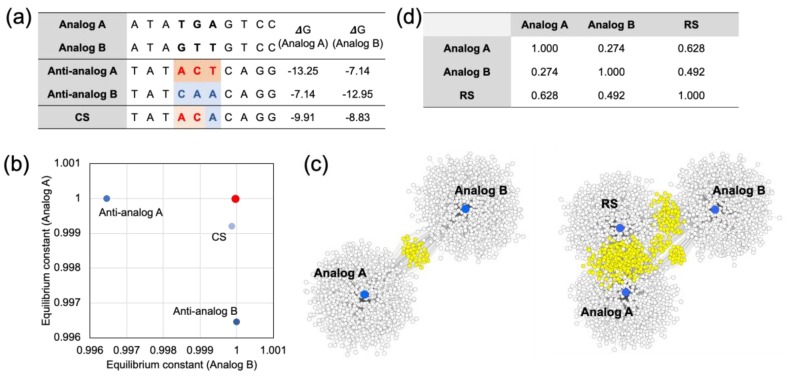
(**a**) The sequence information of the analogs used for calculating the RS. The analogs had three differences in their sequences. A calculated CS was hetero-sequenced for both of the Anti-analogs. The Gibbs free energy values of the sequences show that the CS was not as stable as the Anti-analogs; however, the difference was modest between the two analogs. (**b**) The multiple reaction equilibrium of the CS and Anti-analogs. The equilibrium constant coordinates of the Anti-analogs were biased to their analog, and the distance to the coordinate of perfect hybridization (1,1) (marked with the red dot) was relatively great. Meanwhile, the coordinate of the CS was located closer to the red dot. (**c**) The sociogram mapped with 1000 complementary sequences shows that the RS shared more complementary candidates with the analogs. (**d**) In addition, the Pearson’s correlation coefficients of the Gibbs free energy values against all of the complementary sequences between the analogs and the RS were lower than those of the analogs.

The sequence information of two model analogs with three base mismatches is presented in Figure 3a. When we compared the Gibbs free energy values of the CS and the perfect complement of the analogs (Anti-analogs), it was shown that the CS did not have a minimized Gibbs free energy; the sum of the hybridization yield was higher than for the Anti-analogs. In addition, the calculated multiple reaction equilibrium coordinates showed a closer distance to the (1,1) coordinates than the coordinates of the Anti-analogs (Figure 3b). In the case of Anti-analog A, the hybridization yield against Analog B was less than 0.0035 μM to the perfect hybridization (1,1). Meanwhile, the CS was only less than 0.00013 μM and 0.00089 μM for Analog A and Analog B, respectively. The sum of shorts was lower in the CS compared with the Anti-analogs. Furthermore, the RS was obtained from the CS with complementary sequences. Additionally, the RS and Analogs were mapped with 1000 complementary sequences, and are presented in Figure 3c. The shared complementary sequences are marked with a yellow color. As expected, the RS shared a significant amount of complementary sequences with both Analog A and Analog B. The Pearson’s correlation coefficient of all the Gibbs free energy values calculated from the Analogs was also used to show the similarity of the hybridization profile, and the RS had a higher Pearson’s correlation than the Analogs compared to the value between the Analogs (Figure 3d). The details of the Pearson’s correlation coefficient calculations are presented in Figure S4.

**Figure 4:**
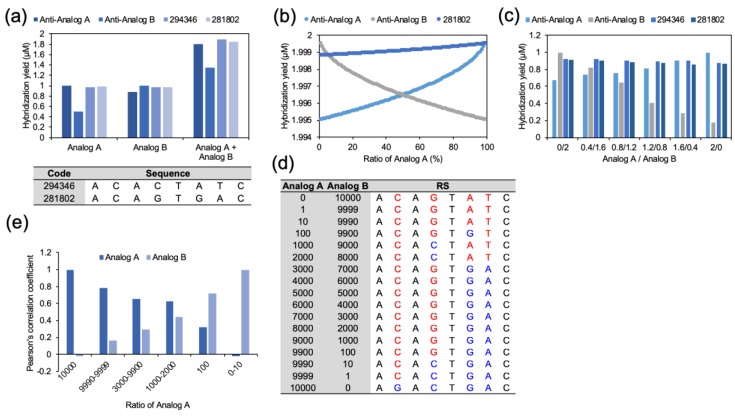
(**a**) Sequence information and Hybridization efficiency of the CS (294346 and 281802) against the analogs. When 1 μM of analog was treated with Anti-analogs and the CSs, the hybridization yields of the analogs were the highest in their own Anti-analog and the lowest in the other’s Anti-analog. Meanwhile, when the analogs were mixed, the CSs showed a higher hybridization yield than the perfect complements. (**b**) The hybridization efficiency of the Anti-analogs and the CSs with analog concentration variation, calculated in silico. (**c**) As expected theoretically, the CSs showed a higher summation of hybridization when the two analogs existed together. In addition, following the ratio diversification of the analogs, a better hybridization efficiency was observed in the mid-region of both the in silico and experimental data. (**d**) The optimized RSs calculated from various concentrations of analogs. (**e**) The Pearson’s correlation coefficients of the optimized RSs against the analogs.

The multiple reactions and hybridization between the analogs and the CS were also proved experimentally. To provide a clear demonstration, we selected two CSs (code numbers: 294346 and 281802). A code number was assigned to every possible complementary sequence, and the sequences of 294,346 and 281,802 are noted in Figure 4a. As shown in Figure 4a, the analogs were reacted with CSs, and their hybridization reactions were measured. For the measurement, analogs were labeled with fluorescent dyes (Cy3 and Cy5), and CSs were labeled with non-fluorescent quencher, IOWA Black. When hybridization between the analogs and the CSs occurs, the fluorescence intensities become weaker. First, 1 μM of an analog (Analog A or Analog B) was combined with 2 μM of its Anti-analogs separately. As expected, the Anti-analogs showed the highest hybridization efficiency with their own analog. However, the hybridization to the other analog was not effective. In the case of anti-Analog B, perfect hybridization was shown with Analog B. Meanwhile, anti-Analog B hybridized to Analog A with only 50.4% efficiency. Even though the CSs showed a lower hybridization efficiency compared to the perfect anti-analogs, the hybridization with both analogs was better. Moreover, in the reaction with the mixed Analogs (1 μM each), CSs showed the highest yield in total hybridization.

This phenomenon was also observed when the concentration of the analogs was verified. Before the actual experiment, the hybridization efficiency of the Anti-analogs and CSs in various concentrations of analogs was calculated in silico (Figure 4b). The concentrations of the analogs were increased from 0 μM to 2 μM, and the sum of the concentration was fixed at 2 μM. At the end-points and nearby, where Analog A or Analog B occupied all of the nucleotides at 2 μM, the perfect complementary sequences showed the highest hybridization efficiencies. However, hybridization of the Anti-analogs significantly decreased with the decrement of their own complements. In contrast, CSs demonstrated sustained hybridization efficiency at all concentrations. The triangle region where CSs showed higher hybridization efficiency well-described the potential of CSs and the RSs. This tendency was also observed in the actual experiments (Figure 4c). The hybridization efficiency of the perfect complementary sequences became lower with the increment of their less-compatible targets, but the CSs showed better hybridization at an Analog A/Analog B ratio from 0.4:1.6 to 1.2:0.8. To make this result more reliable, 100 random analog sets were used to generate the RS (Figure S5). Even though there were differences in the hybridization efficiency values, the results showed solid evidence of the same process in RSs. The Gibbs free energy profile of the analogs and the RSs against all possible complementary sequences were compared using Pearson’s correlation coefficient. Between two-base mismatched analogs, the Pearson’s correlation coefficient was 0.636 ± 0.049. In contrast, the average Pearson’s correlation coefficient between the RS and the analogs was 0.805 ± 0.042. This increment of the coefficient indicated that the RS can delegate the hybridization profile of the analogs. In addition, it was possible to calculate the optimized RSs from various concentrations of analogs. As shown in Figure 4d, two analogs sharing four of a total of eight bases were used for calculating the RS in varied concentrations. Analog concentrations were applied from 0:10,000 to 10,000:0, and optimized RSs were obtained. With the concentration biases, the optimized RSs had greater closeness and a higher Pearson’s correlation coefficient than the dominant analog (Figure 4e).

### 2.3. Generation of RSs from Multiple Sequences

In the coordinate system, the K-means clustering algorithm can generate intuitive and rational centroids for clustering [25,26]. For clustering with the K-means algorithm, the sum of distances from the centroid to the data objects is measured, and the coordinates of centroids are updated to minimize the sum. The centroid itself has a coordinate just like other data objects, although it is not real. Thus, it can be said that the centroid represents the properties of the data objects in the cluster. This is quite similar to the calculation of the RS from the analog sequences. We tried to apply the K-means clustering algorithm to the generation of the RS with multiple sequences. By equating a map of nucleotides obtained from the sociogram with a coordinate system where K-means clustering is performed, it is possible to obtain the RS representing multiple nucleotides similar to the calculation of the centroid using the K-means clustering method. The sum of distance was replaced with the sum of hybridization yield of the CS to each analog, and the maximized hybridization yield of the CS reflects the coordinates of the centroid in the minimized sum of distance of the K-means clustering algorithm.

Several remarkable approaches have been developed to calculate multiple nucleotide equilibrium states. For instance, Robert Dirt and his colleagues developed methodologies for calculating multistrand interactions and the formation of secondary structures by the combination of graph theory and a partition function [6,27,28,29]. However, tremendous resources and calculation times are needed to obtain reasonable results for thousands of reactions simultaneously. Therefore, we applied the calculation in a step-by-step manner. It was shown that the calculation of the RSs from two different analogs was achieved by a simple multiple reaction equilibrium calculation. If we repeat this calculation, it would be possible to discover the RS for a number of sequences. To determine the order of stepwise calculation, the closeness was used. Two analogs with the highest closeness were calculated first to generate their own RS, and the RS participated in the next iterative calculation as one of the analogs. Finally, it was possible to obtain the RS information and its equivalent concentration from the cluster of the analogs.

**Figure 5:**
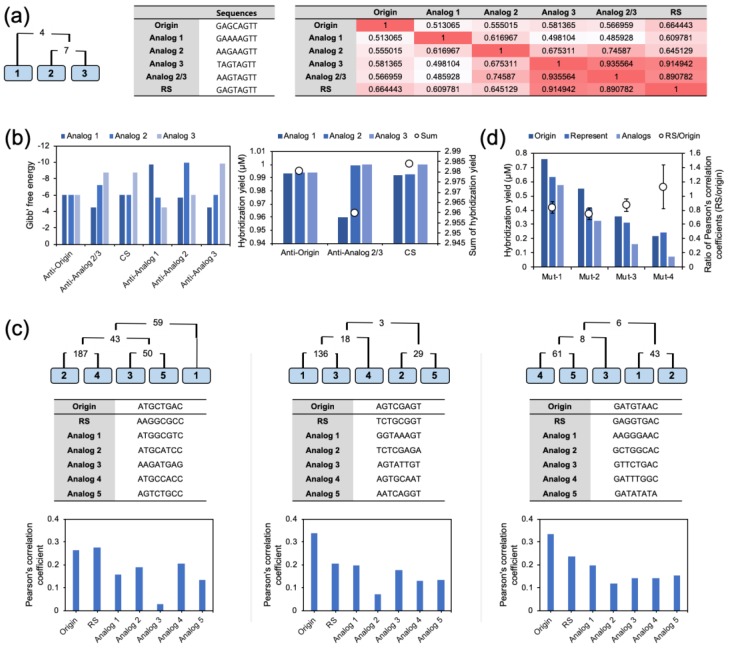
The sequence information of multiple analogs and its calculation tree of the RS. (**a**) Three analogs generated from a single origin with two base mutations. The order of iterative calculation was adjusted by the order of closeness. The Pearson’s correlation coefficient of the final RS also showed a modest increment against Analog 1. (**b**) The Gibbs free energies of the calculated CSs and hybridization yields against the analogs. The sum of hybridization yield was highest in the CSs. (**c**) Three sets of nucleotide clusters with five different analogs. The five analogs were generated from a single origin with three mutations. The RSs were obtained by iterative calculation, and the Pearson’s correlation coefficients were calculated for validation. (**d**) The hybridization yield and the ratio of the Pearson’s correlation coefficients of various mutation base numbers. With an increment of mutation, the functionality of the RS was becoming effective.

To see how the iterative calculation worked, three analogs were generated from an eight-based random origin. The analogs were prepared with two mutations from the origin. The sequence information of the three analogs (Analog 1, Analog 2, and Analog 3) is presented in Figure 5a. The initial concentrations of the analogs were assumed to be 1. In the top 1000 rated complementary sequences-based closeness calculation, Analog 2 and Analog 3 showed the highest closeness. Therefore, the RS of Analog 2 and Analog 3 was calculated first, and named Analog 2/3. Subsequently, the concentration of Analog 2/3 was calculated. The sequences of Analog 2 and Analog 3 were replaced with the sequence and concentration of the calculated Analog 2/3. In succession, Analog 1 and Analog 2/3 were used to calculate the final RS. The Pearson’s correlation coefficients of the origin, Analogs, and RS are noted in Figure 5a. The Pearson’s correlation coefficients of Analog 2/3 against Analog 2 and Analog 3 (0.746 and 0.936, respectively) were higher than the coefficient between Analog 2 and Analog 3 (0.675). In contrast, the Pearson’s correlation coefficient of Analog 2/3 against Analog 1 were not increased. This indicated that the calculated RS was specific to the target analogs. Meanwhile, the RS calculated from Analog 1 and Analog 2/3 showed an increment of the Pearson’s correlation coefficient against the origin and modest Gibbs free energy values. The coefficient value was 0.664, which was higher than the coefficient value of any other Analog. It was remarkable that the Pearson’s correlation of the RS for Analog 2 and Analog 3 was lower than that of Analog 2/3; however, the average coefficient was increased within all the analogs. Moreover, the average Pearson’s correlation coefficient of the initial analogs against the RS was higher than that against the origin. This result not only demonstrates that it was possible to make a RS, but also that its performance might be better than that of its origin.

To validate the RS, the Gibbs free energy and the Pearson’s correlation between the sequences were calculated. As shown in Figure 5b, the Gibbs free energy values of Analog 2/3 were higher than those of the perfect complementary sequences, Anti-Analog 2 for Analog 2, and Anti-Analog3 for Analog 3; however, they were higher than those of the opposite analogs Anti-Analog 2 for Analog 3 and Anti-Analog 3 for Analog 2. The potential of the RS was also revealed in the hybridization yield calculation in Figure 5b. The perfect complementary sequences of the origin, Analog 2/3, and the RS were used to measure the hybridization efficiency. The hybridization yields of the anti-sense origin were similar in the three analogs, and the sum of the constant was 2.980. In the case of Anti-analog 2/3, which was calculated from Analog 2 and Analog 3, the hybridization yields of Analog 2 and Analog 3 were increased compared to the Anti-analogs; however, the constant of Analog 1 was significantly decreased. Thus, the sum of hybridization yields was decreased overall (2.960). Finally, the CS showed a recovered hybridization yield of Analog 1, and the sum was the highest (2.984). Through this result, the potential of the generated RS was proved directly.

For the analysis of multiple nucleotides, the RSs were obtained by the same procedure with five analogs and three mismatched bases. In the model sets, the average Pearson’s correlations of the origin and the RS against the initial analogs were calculated and were compared. Interestingly, the Pearson’s correlation coefficient of the RS was not always higher or lower than the coefficient of the origin. As shown in Figure 5c, the Pearson’s correlation coefficient of the RS was higher or lower than the Pearson’s correlation of the origin in a sequence-dependent manner; however, statistically, it was higher than the coefficients of the analogs. To obtain solid evidence, we created a random set of 100 analogs and measured the averages (Figure S6). The Pearson’s correlation coefficients of the origin, the RS, and the analogs were 0.359 ± 0.074, 0.312 ± 0.0821, and 0.163 ± 0.0922, respectively. This result indicated that our procedure can generate RSs from multiple nucleotides statistically; however, they are not optimized as in the origin. Also, to clarify the effect of the number of mutations in the analogs, 30 sets of analogs were prepared with 1–4 mutations from the eight-based origin, and the Pearson’s correlation coefficients were measured (Figure S7). As shown in Figure 5d, the average Pearson’s correlation coefficient of the origin against the analogs was decreased with the increment of the number of mutations because the difference of the sequences led to less similarity in the hybridization profile. The decrement of the Pearson’s correlation coefficient was also observed in the RS; however, the amount of decrease was smaller than that of the origins. Thus, the ratio of the Pearson’s correlation coefficient (RS/origin) was increased with the number of mutations. When the number of mutations was 4, the average coefficient of the RS was higher than that of the origins. 

We believe this shortage was generated from the imprecise calculation of the Gibbs free energy and the equilibrium constant. Especially, since there are several factors to consider in nucleotide hybridization, such as secondary structure, the equilibrium constant calculation with simple thermodynamic principles may not be sufficient for specific optimization for RS generation. To overcome these inaccuracies, more complex and simultaneous calculations could be applied in the Gibbs energy and equilibrium state calculation process. However, it was demonstrated that the RS showed a much higher correlation with the analogs than any of the single analogs.

## 3. Discussion

One of the main characteristics of nucleic acid as a mediator of information is the specific recognition of complementary sequences. It is well-known that mRNA-microRNA interactions, DNA-DNA interactions, and even specific interactions between nucleic acid and proteins are based on this characteristic. To manipulate the massive number of biological pathways, the nucleotide sequence should be diversified and specialized. However, what we should achieve is not a higher functionality, but evidence of concentrated information. Therefore, as a proof of concept, we tried to concentrate the information underneath multiple nucleotides. For convenience, 8–10 nucleotide sequences were used, and the calculation formula was simplified. Since the study of the RS presented in this paper is in the proof-of-concept stage, it is not sufficient to demonstrate the actual biological significance. However, we would like to argue for the potential of the RS in an understanding of the harmonic regulation of nucleotide sequence clusters presented in such fields as RNA interference. As suggested in the manuscript, some of the microRNAs with similar sequences effectively coordinate the regulation of mRNAs with a similar metabolic pathway. A number of microRNA analogs, generated through a single regulatory mechanism, are involved in the stepwise regulation of specific mRNAs. However, the process of an efficient understanding of intracellular metabolism may be possible by recognizing the profile of the overall profile, rather than the specific quantification of each analog. However, in order to prove the hypothesis above, further deep research must be conducted. First, the number of bases in the analogs must increase significantly up to the biological molecular level. For instance, microRNAs generally consist of 18–25 bases. To apply the method presented in this paper, 4^18^~4^25^ kinds of all possible nucleotide sequences should be separately calculated and compared. This causes a significant increment in computational burden, and it would be inefficient to operate. Likewise, the development of algorithms that enable effective computation of the RS should be achieved. This will allow for in-depth discussion of the biologic significance of the RS. We believe this approach to multiple nucleotide analysis could be a toehold in the interpretation of biological networks.

## 4. Materials and Methods

### 4.1. Sequence Design Method for the RS and CS

The random sequence of the origin was arbitrary, the cumulative mutation and random mutation for generating the analogs were conducted using Python v2.7 (Python Software Foundation, DE, USA), and the random function in the random module was used for generating point mutations.

The RS containing information from multiple analog nucleotides (Mutants) should satisfy the following criteria:

1. maintain sequence similarity with analog nucleotides.
2. delegate the hybridization profile of the multiple nucleotides.

It is intuitively reasonable that the RS should have a similarity of sequences with analog nucleotides in the sense of representing them. Meanwhile, since the fundamental functional feature of a nucleotide is hybridization to their complementary, the RS should represent the hybridization profile of analog nucleotides. To fulfill the above criteria, the synthesis of the RS was based on its Gibbs free energy. First, we calculated the Gibbs free energy of every possible complementary sequence against the mutants. The most favorable complementary sequence with the highest yield of hybridization to the entire mutants was selected and named the CS. Finally, we obtained the RS from the CS, and calculated the concentration of the RS. The characteristics of the RS were verified by computational and statistical analysis methods in silico. In addition, some of the nucleotide sequences were selected and analyzed to demonstrate if the same tendency occurred in actual experiments.

The CS was generated from a group of multiple nucleotides by an iterative calculation. For the iterative process, the principle of the K-means clustering method was adopted. To determine the order of iterative calculations, we introduced a concept of “Closeness”. To create a centroid representing a cluster in K-means clustering, we need an index of the coordinates or the relative distance of each object. To reflect this index, the closeness was used to indicate the relative distance (hybridization similarity) between nucleotides. The closeness showed the hybridization profile similarity among multiple nucleotides, and it was calculated by counting the number of shared sequences of the highest hybridization energy. For instance, it is possible to make a list of 100 sequences having the lowest hybridization Gibbs free energy against sequence A and sequence B, respectively. If 10 sequences in the list overlapped, then the closeness of A and B is 10. This term efficiently indicated the similarity of the hybridization profile between sequences.

### 4.2. Experiments

All of the nucleotides used for the actual experiments were purchased from Integrated DNA Technologies, Inc. (Coralville, IA, USA). Initially, lyophilized nucleotides were dissolved in TE buffer (10 mM Tris, pH 8.0, 0.1 mM EDTA) to 100 μM concentration. The nucleotides were mixed at a ratio corresponding to each experiment with 100 mM NaCl for the hybridization process. The annealing process was performed in a thermocycler provided by the Mastercycler Pro of Eppendorf (Westbury, NY, USA). After heating at 95 °C for 5 min, stepwise temperature was decreased from 95 °C to 25 °C at 0.5 °C per minute. The fluorescence intensities were measured using a SpectraMax M5 provided by Molecular Devices, Inc. (Sunnyvale, CA, USA).

### 4.3. Calculation of Gibbs Free Energy, Pearson’s Correlation Coefficient, Multiple Equilibrium Constant, and Closeness

All calculations were conducted using Python v2.7, including the Gibbs free energy, Pearson’s correlation coefficient, multiple equilibrium constant, and closeness. NumPy v1.8.0rc1 (NumFOCUS, Austin, TX, USA) and SciPy v0.13.0b1 (NumFOCUS, Austin, TX, USA) were used for algorithmic efficiency. Details of the calculation methods and formula are presented in Figures S1–4.

### 4.4. Sociogram

Cytoscape v3.6.0 was used to visualize the sociogram [30]. The origin, the analogs, and the complementary candidates were used as nodes. The complementary candidates were connected to their relevant origin or analogs. The position of nodes was determined through a prefuse force-directed layout.

### 4.5. Data and Code Availability

All relevant data and code are available at https://github.com/seungwonshinDr/RS.

## Supplementary Materials

The following are available online, Figure S1: Calculation of Gibbs free energy, Figure S2: Calculation of the multiple reaction equilibrium of two analogs, Figure S3: Calculation of the concentration of the RS, Figure S4: Calculation of the Pearson’s correlation coefficient, Figure S5: The random sets of two analogs and the calculation of the RS, Figure S6: The random sets of five analogs (eight bases, three mutations), and the calculation of the RS, Figure S7: The random sets of five analogs with mutation number variation.
