# Supplementary material for: Revealing the Presence of a Symbolic Sequence Representing Multiple Nucleotides Based on K-Means Clustering of Oligonucleotides
Source: Molecules. 2019 Jan 18;24(2):348. doi: 10.3390/molecules24020348 (PMC6359743; doi:10.3390/molecules24020348)
Supplement: Supplementary file 1 [file molecules-24-00348-s001.pdf]

Supplementary Information

Revealing the Presence of Symbolic Sequence Representing Multiple Nucleotides: Based on K-means Clustering of Oligonucleotides

Contents

**Figure S1**	Calculation of Gibb's free energy.
**Figure S2**	Calculation of the multiple reaction equilibrium of two analogs.
**Figure S3**	Calculation of the concentration of the RS.
**Figure S4**	Calculation of the Pearson's correlation coefficient.
**Figure S5**	The random sets of two analogs and the calculation of the RS.
**Figure S6**	The random sets of five analogs (8 bases, 3 mutations) and the calculation of the RS.
**Figure S7**	The random sets of five analogs with mutation number variation.
**Appendix**	The codes for the calculation of the representative sequences.

Supplementary 1. Calculation of Gibb's free energy.

The nearest-neighbor model was used for Gibbs's free energy calculation. In the general usage, the nearest-neighbor parameter of the nucleic acid duplex and terminal base pairs parameters should be included to calculate the enthalpy and entropy of hybridization. We considered the nearest-neighbor parameter in the complementary base pairing for facile calculation. The nearest-neighbor parameters were referenced from a previous study, [Ref:1] and the reaction condition was considered as 1 M NaCl, 25 °C and pH 7.

**Interaction**	$\Delta \boldsymbol{H}2^\circ$ **(kcal/mol)**	$\Delta \boldsymbol{S}2^\circ$ **(cal/ K mol)**	$\Delta \boldsymbol{G}2^\circ$ **(kcal/mol)**
**AA/TT**	-9.1	-24.0	-1.9
**AT/TA**	-8.6	-23.9	-1.5
**TA/AT**	-6.0	-16.9	-0.9
**CA/GT**	-5.8	-12.9	-1.9
**GT/CA**	-6.5	-17.3	-1.3
**CT/GA**	-7.8	-20.8	-1.6
**GA/CT**	-5.6	-13.5	-1.6
**CG/GC**	-11.9	-27.8	-3.6
**GC/CG**	-11.1	-26.7	-3.1
**GG/CC**	-11.0	-26.6	-3.1

Gibb's free energy between two stands were calculated with the summation of $\Delta \boldsymbol{G}2^\circ$ in reliable interactions.

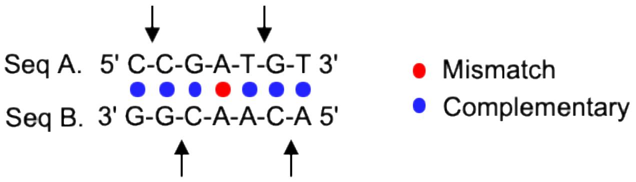

$$\Delta G_{AB} = \Delta G(CC|GG) + \Delta G(CG|GC) + \Delta G(TG|AC) + \Delta G(GT|CA)$$

Supplementary 2. Calculation of the multiple reaction equilibrium of two analogs.

The reaction equation of the analogs with the CS was written as below.

$$[Analog\ 1] + [CS] = [Complex_{A1}] \cdots \Delta G_{A1} = -RTlnK_{A1}$$

$$[Analog\ 2] + [CS] = [Complex_{A2}] \cdots \Delta G_{A2} = -RTlnK_{A2}$$

The multiple reaction of Analog 1 and Analog 2 with the CS was written as below.

	in	*out*	η
Analog 1	1	$1 - x_{A1}$	$\frac{1 - x_{A1}}{4 - x_{A1} - x_{A2}}$
Analog 2	1	$1 - x_{A2}$	$\frac{1 - x_{A2}}{4 - x_{A1} - x_{A2}}$
CS	2	$2 - x_{A1} - x_{A2}$	$\frac{2 - x_{A1} - x_{A2}}{4 - x_{A1} - x_{A2}}$
ComplexA1	0	x_{A1}	$\frac{x_{A1}}{4 - x_{A1} - x_{A2}}$
ComplexA2	0	x_{A2}	$\frac{x_{A2}}{4 - x_{A1} - x_{A2}}$
**Total**		$4 - x_{A1} - x_{A2}$	

The amount of hybridization of Analog 1 and Analog 2 was referred to as x_{A1} and x_{A2}, respectively.

$$K_{A1} = \frac{[Complex_{A1}]}{[Analog\ 1][CS]} = \frac{x_{A1}(4 - x_{A1} - x_{A2})}{(1 - x_{A1})(2 - x_{A1} - x_{A2})}$$

$$K_{A2} = \frac{[Complex_{A2}]}{[Analog\ 2][CS]} = \frac{x_{A2}(4 - x_{A1} - x_{A2})}{(1 - x_{A2})(2 - x_{A1} - x_{A2})}$$

The reaction constants, K_{A1} and K_{A1}, were obtained from the Gibb's free energies of hybridization. And x_{A1} and x_{A2} were obtained by solving the simultaneous equation.

Supplementary 3. Calculation of the concentration of the RS.

From the above calculation of multiple reaction equilibrium of two analogs, it was possible to obtain x_A and x_B. The hybridization yield of the RS (x_R) was equal to the sum of the hybridization yield of the analogs.

$$x_{A1} + x_{A2} = x_R$$

The reaction equation of the RS and the CS was written as below.

$$[RS] + [CS] = [Complex_R] \cdots \Delta G_R = -RTlnK_R$$

The reaction of the RS and the CS was also written as below.

	in	*out*	η
CS	x	$x - x_R$	$\frac{x - x_R}{2x - x_R}$
RS	x	$x - x_R$	$\frac{x - x_R}{2x - x_R}$
*Complex*R	0	x_R	$\frac{x_R}{2x - x_R}$
**Total**		$2x - x_R$	

The x indicates the amount of CS and the RS that equaled the hybridization yield of both of the analogs. The x was obtained from the equation of K_R. The value of K_R was obtained from the Gibb's free energy of hybridization.

$$K_R = \frac{[Complex_R]}{[CS][RS]} = \frac{x_R(2x - x_R)}{(x - x_R)^2}$$

Supplementary 4. Calculation of the Pearson's correlation coefficient.

The similarity of the hybridization profile of the nucleotides was measured with the Pearson's correlation coefficient. All possible sequences were prepared, and the hybridization profiles against the nucleotides of interest were listed. In the case of the 8-based origin, 48 (65,536) sequences existed. From AAAAAAAA to CCCCCCCC, 65,536 of the hybridization Gibb's free energy values against the nucleotides were listed.

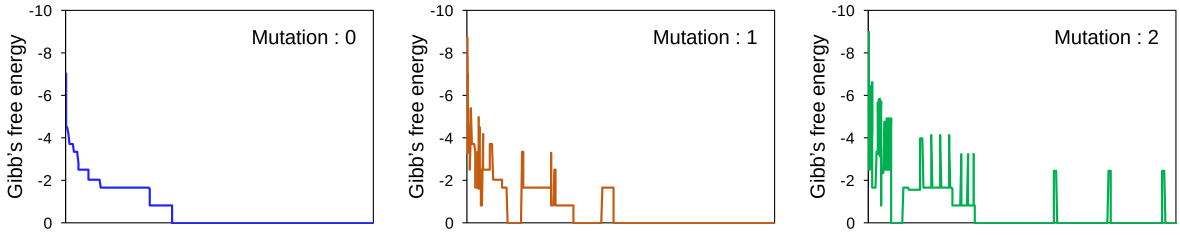

In the above graphs, the Gibb's energy profiles of the origin (number of mutated bases: 0) and the analogs (number of mutated bases: 1,2) are noted. As the mutations accumulated, the hybridization energy profile difference became more pronounced. This difference was quantified with the Pearson's correlation coefficient.

The Pearson's correlation coefficient was calculated with the "pearsonr" function supported by SciPy.org, and the calculation formula is noted below.

$$P_{AB} = \frac{\sum(\Delta G_{Ai} - \Delta\bar{G}_A) \times (\Delta G_{Bi} - \Delta\bar{G}_B)}{\sqrt{\sum(\Delta G_{Ai} - \Delta\bar{G}_A)^2 \times \sum(\Delta G_{Bi} - \Delta\bar{G}_B)^2}}$$

In the equation, the Pearson's correlation coefficient of nucleotide A and nucleotide B (P_{AB}) was obtained from the Gibb's free energy of each sequence (ΔG_{Ai}, ΔG_{Bi}) and the average of the Gibb's free energy ($\Delta\bar{G}_A$, $\Delta\bar{G}_B$).

Supplementary 5. The random sets of two analogs, and the calculation of the RS.

			Pearson correlation coefficient				Closeness		
		Sequences	Analog-1 /Analog-2	Analog-1 /Represent	Analog-2 / Represent		Analog-1	Analog-2	Represent
**1**	Analog-1	AATTACAGAG	0.438180366	0.749938668	0.682072553	Analog-1	0	20	103
	Analog-2	AATTCCCGAG				Analog-2	20	0	58
	Represent	AATTCCAGAG				Represent	103	58	0
**2**	Analog-1	GGGAGCTAGA	0.586027263	0.83348672	0.761853347	Analog-1	0	43	364
	Analog-2	GGCAGCTGGA				Analog-2	43	0	202
	Represent	GGGAGCTGGA				Represent	364	202	0
**3**	Analog-1	GCTTCTTCCG	0.64450625	0.795466311	0.85134936	Analog-1	0	79	154
	Analog-2	GCTCCTGCCG				Analog-2	79	0	508
	Represent	GCTTCTGCCG				Represent	154	508	0
**4**	Analog-1	ACACGTAGTG	0.787937087	1	0.787937087	Analog-1	0	253	997
	Analog-2	TGACGTAGTG				Analog-2	253	0	256
	Represent	ACACGTAGTG				Represent	997	256	0
**5**	Analog-1	GGTCGGCCGA	0.749160567	0.815741106	0.94113895	Analog-1	0	319	319
	Analog-2	AGCCGGCCGA				Analog-2	319	0	847
	Represent	GGCCGGCCGA				Represent	319	847	0
**6**	Analog-1	GGGTCGGGCC	0.68494884	0.897354199	0.800550667	Analog-1	0	154	634
	Analog-2	CGGTCAGGCC				Analog-2	154	0	232
	Represent	CGGTCGGGCC				Represent	634	232	0
**7**	Analog-1	GAACACTAAC	0.58205112	0.7204558	0.811195095	Analog-1	0	106	154
	Analog-2	GTATACTAAC				Analog-2	106	0	448
	Represent	TAATACTAAC				Represent	154	448	0
**8**	Analog-1	CTGTGCAGGC	0.622090195	0.756491315	0.756491315	Analog-1	0	107	133
	Analog-2	CTGCACAGGC				Analog-2	107	0	106
	Represent	CTGCGCAGGC				Represent	133	106	0
**9**	Analog-1	TCAGGTCCTG	0.555773089	0.579479604	0.647662634	Analog-1	0	47	58
	Analog-2	TCAGTTACTG				Analog-2	47	0	106
	Represent	TCATGTACTG				Represent	58	106	0
**10**	Analog-1	TAAGCGACAC	0.743581193	0.812366894	0.934024997	Analog-1	0	322	355
	Analog-2	TAAGCGAGAA				Analog-2	322	0	757
	Represent	TAAGCGAGAC				Represent	355	757	0
**11**	Analog-1	CGGTTTCCTA	0.600829533	0.76807833	0.836949481	Analog-1	0	184	223
	Analog-2	TGTTTTCCTA				Analog-2	184	0	346
	Represent	CGTTTTCCTA				Represent	223	346	0
**12**	Analog-1	TGTCGCAAAA	0.602818857	0.762965485	0.779691377	Analog-1	0	49	67
	Analog-2	TGGTGCAAAA				Analog-2	49	0	205
	Represent	TGTTGCAAAA				Represent	67	205	0
**13**	Analog-1	TCAGAACAGG	0.593242506	0.761006478	0.866671565	Analog-1	0	103	139
	Analog-2	TTAGCACAGG				Analog-2	103	0	613
	Represent	TCAGCACAGG				Represent	139	613	0
**14**	Analog-1	AGCGCTACGG	0.618953875	0.873942502	0.77496401	Analog-1	0	73	403
	Analog-2	AGAGCTCCGG				Analog-2	73	0	169
	Represent	AGCGCTCCGG				Represent	403	169	0

**15**	Analog-1	TCTGAGTCAC	0.839214996	0.911725822	0.931395725	Analog-1	0	442	685
	Analog-2	ACTGAGTCAT				Analog-2	442	0	520
	Represent	ACTGAGTCAC				Represent	685	520	0
**16**	Analog-1	CGCGCTACAT	0.778845775	0.960868125	0.824931477	Analog-1	0	250	860
	Analog-2	CGCGATACAG				Analog-2	250	0	250
	Represent	CGCGCTACAG				Represent	860	250	0
**17**	Analog-1	TGGGAACATT	0.510124078	0.686874782	0.74868737	Analog-1	0	38	214
	Analog-2	TTGGAAGATT				Analog-2	38	0	160
	Represent	AGGGAAGATT				Represent	214	160	0
**18**	Analog-1	CCAGGGCAAG	0.530828543	0.8108642	0.724556199	Analog-1	0	29	160
	Analog-2	CCAGCGCGAG				Analog-2	29	0	85
	Represent	CCAGGGCGAG				Represent	160	85	0
**19**	Analog-1	TCTCATTGTA	0.6450014	0.758110502	0.87608566	Analog-1	0	148	325
	Analog-2	ACTCATTTTA				Analog-2	148	0	532
	Represent	TCTCATTTTA				Represent	325	532	0
**20**	Analog-1	GTCCGATAAA	0.553668459	0.791176888	0.794605507	Analog-1	0	44	157
	Analog-2	GTTCGGTAAA				Analog-2	44	0	193
	Represent	GTCCGGTAAA				Represent	157	193	0
**21**	Analog-1	TGAGATCAGA	0.657193023	0.775650579	0.789161944	Analog-1	0	100	148
	Analog-2	TGAGGACAGA				Analog-2	100	0	160
	Represent	TGAGGTCAGA				Represent	148	160	0
**22**	Analog-1	TACTAGGGGC	0.618467584	0.748434789	0.852755601	Analog-1	0	73	100
	Analog-2	TACTTGAGGC				Analog-2	73	0	376
	Represent	TACTAGAGGC				Represent	100	376	0
**23**	Analog-1	ACTAATTTTG	0.614305209	0.773301003	0.742852884	Analog-1	0	70	250
	Analog-2	ACTACCTTTG				Analog-2	70	0	88
	Represent	ACTACTTTTG				Represent	250	88	0
**24**	Analog-1	AACGACGCGA	0.604974751	0.807520864	0.817800753	Analog-1	0	71	154
	Analog-2	AAGGGCGCGA				Analog-2	71	0	496
	Represent	AACGGCGCGA				Represent	154	496	0
**25**	Analog-1	ATTTTCCGAC	0.661474651	0.86646598	0.753970246	Analog-1	0	123	568
	Analog-2	GTTTTCCGGC				Analog-2	123	0	151
	Represent	GTTTTCCGAT				Represent	568	151	0
**26**	Analog-1	TAGGGCAGCG	0.694782169	0.801611591	0.910211162	Analog-1	0	112	130
	Analog-2	TAGGGCGGCA				Analog-2	112	0	595
	Represent	TAGGGCGGCG				Represent	130	595	0
**27**	Analog-1	GTCCGCTATA	0.504162618	0.590226822	0.896366823	Analog-1	0	13	16
	Analog-2	GTCCCCTTTA				Analog-2	13	0	547
	Represent	GTCCCCTATA				Represent	16	547	0
**28**	Analog-1	GGTGCCGGAA	0.794322929	0.95079162	0.84939432	Analog-1	0	472	889
	Analog-2	GATGCCGGAC				Analog-2	472	0	517
	Represent	GGTGCCGGAC				Represent	889	517	0
**29**	Analog-1	TACTGCTAAG	0.545790384	1	0.545790384	Analog-1	0	43	1000
	Analog-2	TACTTTTAAG				Analog-2	43	0	43
	Represent	TACTGCTAAG				Represent	1000	43	0
**30**	Analog-1	TGGCCTAGTG	0.658033767	0.830012894	0.846830439	Analog-1	0	133	202
	Analog-2	TGGCCGAGAG				Analog-2	133	0	652
	Represent	TGGCCGAGTG				Represent	202	652	0

**31**	Analog-1	AATTCTGGCG	0.690785936	0.825886692	0.881367324	Analog-1	0	220	424
	Analog-2	AATCCTGGCA				Analog-2	220	0	451
	Represent	AATCCTGGCG				Represent	424	451	0
**32**	Analog-1	CGAGTCTACG	0.671770957	0.810727285	0.876787703	Analog-1	0	175	292
	Analog-2	AGAGTGTACG				Analog-2	175	0	559
	Represent	CGAGTGTACG				Represent	292	559	0
**33**	Analog-1	CACATCTAAT	0.687172825	0.77485809	0.833726426	Analog-1	0	154	265
	Analog-2	CACAATTAAT				Analog-2	154	0	229
	Represent	CACATTTAAT				Represent	265	229	0
**34**	Analog-1	GTAAGCCACC	0.638035317	0.755482255	0.87710214	Analog-1	0	84	130
	Analog-2	GTAAACCACA				Analog-2	84	0	580
	Represent	GTAAACCACC				Represent	130	580	0
**35**	Analog-1	CGATGATATC	0.52371255	0.689116076	0.783593814	Analog-1	0	76	205
	Analog-2	CTGTGATATC				Analog-2	76	0	181
	Represent	CTATGATATC				Represent	205	181	0
**36**	Analog-1	TGCGTCGTTC	0.540670937	0.669348251	0.657288517	Analog-1	0	55	76
	Analog-2	TGCTTCCTTC				Analog-2	55	0	319
	Represent	TGCTTCGATC				Represent	76	319	0
**37**	Analog-1	CCAAAATAAA	0.633969766	0.804266998	0.724705947	Analog-1	0	82	403
	Analog-2	CCAAAGCAAA				Analog-2	82	0	109
	Represent	CCAAAACAAA				Represent	403	109	0
**38**	Analog-1	TGCGATATCC	0.652042813	0.799390261	0.842705396	Analog-1	0	139	388
	Analog-2	TGCGTTATTC				Analog-2	139	0	277
	Represent	TGCGATATTC				Represent	388	277	0
**39**	Analog-1	AGGGCACGTG	0.646090225	0.770861831	0.771064305	Analog-1	0	76	103
	Analog-2	AGGGTGCGTG				Analog-2	76	0	103
	Represent	AGGGCGCGTG				Represent	103	103	0
**40**	Analog-1	CAGCTGTGAA	0.621610422	0.705127099	0.788466	Analog-1	0	118	52
	Analog-2	CAAGTGTGAA				Analog-2	118	0	184
	Represent	CAGGTGTGAA				Represent	52	184	0
**41**	Analog-1	TGTGAGAACT	0.73091439	0.826666161	0.894814939	Analog-1	0	127	193
	Analog-2	TGTTAGAACA				Analog-2	127	0	481
	Represent	TGTTAGAACT				Represent	193	481	0
**42**	Analog-1	GGTCTTCGTT	0.586573197	0.742653499	0.823931807	Analog-1	0	82	160
	Analog-2	CGTCTTTGTT				Analog-2	82	0	280
	Represent	GGTCTTTGTT				Represent	160	280	0
**43**	Analog-1	GTCATGGGGA	0.554788846	0.732213615	0.802257264	Analog-1	0	38	67
	Analog-2	GTCCTGTGGA				Analog-2	38	0	460
	Represent	GTCATGTGGA				Represent	67	460	0
**44**	Analog-1	GATTACATCG	0.625128645	0.835772708	0.710568879	Analog-1	0	64	415
	Analog-2	GATGCCATCG				Analog-2	64	0	100
	Represent	GATGACATCG				Represent	415	100	0
**45**	Analog-1	TTCTCTTCAG	0.455268631	0.670219881	0.79842901	Analog-1	0	23	112
	Analog-2	TTCTGTTCGG				Analog-2	23	0	286
	Represent	TTCTCTTCGG				Represent	112	286	0
**46**	Analog-1	AAACTTATGC	0.613435392	0.837533919	0.685630387	Analog-1	0	64	331
	Analog-2	AAACTTGCGC				Analog-2	64	0	85
	Represent	AAACTTGTGC				Represent	331	85	0

**47**	Analog-1	TGGGGATGCG	0.734510627	0.800840289	0.93140574	Analog-1	0	133	169
	Analog-2	AGAGGATGCG				Analog-2	133	0	727
	Represent	TGAGGATGCG				Represent	169	727	0
**48**	Analog-1	GACAAAAGCA	0.610308574	0.80354689	0.830161417	Analog-1	0	67	208
	Analog-2	GAAAAAGGCA				Analog-2	67	0	418
	Represent	GACAAAGGCA				Represent	208	418	0
**49**	Analog-1	AGCCGCAATG	0.697664138	0.826332327	0.746039234	Analog-1	0	160	388
	Analog-2	AGCCGTGATG				Analog-2	160	0	118
	Represent	AGCCGCGATG				Represent	388	118	0
**50**	Analog-1	CACAGAGGCG	0.686131593	1	0.686131593	Analog-1	0	112	1000
	Analog-2	CACAGAGGGA				Analog-2	112	0	112
	Represent	CACAGAGGCG				Represent	1000	112	0
**51**	Analog-1	TATCGGCTGC	0.527691874	0.784039431	0.784509563	Analog-1	0	41	159
	Analog-2	TATCAGCGGC				Analog-2	41	0	199
	Represent	TATCGGCGGC				Represent	159	199	0
**52**	Analog-1	ACGGTAGTCC	0.74325368	0.837266143	0.916861673	Analog-1	0	238	268
	Analog-2	ACGGGAGTCT				Analog-2	238	0	733
	Represent	ACGGGAGTCC				Represent	268	733	0
**53**	Analog-1	TTCTGCAGGC	0.729438897	0.94445157	0.783592459	Analog-1	0	307	802
	Analog-2	GTCCGCAGGC				Analog-2	307	0	334
	Represent	GTCTGCAGGC				Represent	802	334	0
**54**	Analog-1	ACTGGATGCA	0.627455038	0.878105474	0.776691032	Analog-1	0	124	424
	Analog-2	GCTGGATGAA				Analog-2	124	0	388
	Represent	GCTGGATGCA				Represent	424	388	0
**55**	Analog-1	GCTTGCAAAA	0.551537016	0.70501042	0.853010773	Analog-1	0	71	361
	Analog-2	GGTTGCTAAA				Analog-2	71	0	343
	Represent	GGTTGCAAAA				Represent	361	343	0
**56**	Analog-1	TCTGTCGTCA	0.825021686	0.899003995	0.92664835	Analog-1	0	439	577
	Analog-2	ACTGTCGTCC				Analog-2	439	0	748
	Represent	TCTGTCGTCC				Represent	577	748	0
**57**	Analog-1	ATCTAGTGGA	0.689535699	0.796417811	0.800135391	Analog-1	0	124	160
	Analog-2	ATCAGGTGGA				Analog-2	124	0	361
	Represent	ATCTGGTGGA				Represent	160	361	0
**58**	Analog-1	TTGCTCGTAT	0.673986693	0.94270558	0.741704683	Analog-1	0	142	616
	Analog-2	CTTCTCGTAT				Analog-2	142	0	172
	Represent	CTGCTCGTAT				Represent	616	172	0
**59**	Analog-1	GGTTAAAACA	0.609447098	0.759359605	0.849045924	Analog-1	0	124	241
	Analog-2	GGTTAATAAA				Analog-2	124	0	442
	Represent	GGTTAAAAAA				Represent	241	442	0
**60**	Analog-1	ACCCCGCGTA	0.895281196	0.964676098	0.931563189	Analog-1	0	735	828
	Analog-2	CCCCCGCGTG				Analog-2	735	0	802
	Represent	ACCCCGCGTG				Represent	828	802	0
**61**	Analog-1	GAGATGTAGC	0.675052442	0.764349401	0.820487665	Analog-1	0	187	271
	Analog-2	GAGAACTAGC				Analog-2	187	0	316
	Represent	GAGATCTAGC				Represent	271	316	0
**62**	Analog-1	CATGAAATGA	0.490698972	0.70550628	0.833048378	Analog-1	0	32	196
	Analog-2	CGTAAAATGA				Analog-2	32	0	289
	Represent	CGTGAAATGA				Represent	196	289	0

**63**	Analog-1	CTCATCGAAT	0.630041277	0.69683669	0.917717615	Analog-1	0	85	91
	Analog-2	CTCATTGAAC				Analog-2	85	0	469
	Represent	CTCATTGAAT				Represent	91	469	0
**64**	Analog-1	TAGATGTCTT	0.7419982	0.816703453	0.924147495	Analog-1	0	235	496
	Analog-2	TTGATGTCTC				Analog-2	235	0	595
	Represent	TTGATGTCTT				Represent	496	595	0
**65**	Analog-1	CCCGGAGGGC	0.761857939	0.864906961	0.904567694	Analog-1	0	283	472
	Analog-2	CCCGGTGGGT				Analog-2	283	0	496
	Represent	CCCGGTGGGC				Represent	472	496	0
**66**	Analog-1	TCATGCAAGA	0.589630173	0.717011284	0.842144884	Analog-1	0	79	103
	Analog-2	GCATTCAAGA				Analog-2	79	0	316
	Represent	TCATTCAAGA				Represent	103	316	0
**67**	Analog-1	TGTTATGTGA	0.637202526	0.833400762	0.833971585	Analog-1	0	136	391
	Analog-2	TGTGATGTTA				Analog-2	136	0	379
	Represent	TGTGATGTGA				Represent	391	379	0
**68**	Analog-1	CGATGATAAA	0.689338736	0.878130466	0.83105892	Analog-1	0	202	460
	Analog-2	AGATGAAAAA				Analog-2	202	0	316
	Represent	CGATGAAAAA				Represent	460	316	0
**69**	Analog-1	GCATATCAAA	0.524225484	0.744554226	0.773448994	Analog-1	0	73	163
	Analog-2	GCGTAGCAAA				Analog-2	73	0	217
	Represent	GCATAGCAAA				Represent	163	217	0
**70**	Analog-1	ATGAGAATCA	0.527900395	0.712661688	0.859116485	Analog-1	0	35	145
	Analog-2	ACGATAATCA				Analog-2	35	0	292
	Represent	ACGAGAATCA				Represent	145	292	0
**71**	Analog-1	CGTATGTTCG	0.712010501	0.791766032	0.841236846	Analog-1	0	196	256
	Analog-2	CGTAACTTCG				Analog-2	196	0	418
	Represent	CGTATCTTCG				Represent	256	418	0
**72**	Analog-1	CACTTGCAAC	0.653396755	0.794076594	0.865647155	Analog-1	0	65	131
	Analog-2	CACATGCCAC				Analog-2	65	0	520
	Represent	CACTTGCCAC				Represent	131	520	0
**73**	Analog-1	CACCCTGATT	0.556663729	0.701963399	0.833569325	Analog-1	0	55	64
	Analog-2	CACACTGAGT				Analog-2	55	0	658
	Represent	CACACTGATT				Represent	64	658	0
**74**	Analog-1	CCCCTAAGAA	0.534449115	0.596700302	0.767129835	Analog-1	0	40	37
	Analog-2	CCGATAAGAA				Analog-2	40	0	154
	Represent	CCGCTAAGAA				Represent	37	154	0
**75**	Analog-1	GGGCCCTGAC	0.756252749	0.843751245	0.915286507	Analog-1	0	358	544
	Analog-2	TGGCCCTCAC				Analog-2	358	0	613
	Represent	GGGCCCTCAC				Represent	544	613	0
**76**	Analog-1	TACAGTTCTA	0.485499728	0.72381695	0.789895642	Analog-1	0	44	169
	Analog-2	TGCACTTCTA				Analog-2	44	0	226
	Represent	TGCAGTTCTA				Represent	169	226	0
**77**	Analog-1	ATCCCGGGCG	0.658555389	0.789530248	0.803409385	Analog-1	0	166	193
	Analog-2	ATCCCGGAAG				Analog-2	166	0	469
	Represent	ATCCCGGACG				Represent	193	469	0
**78**	Analog-1	CCATTCAATA	0.466148275	0.599491606	0.758803347	Analog-1	0	25	112
	Analog-2	CGGTTCAATA				Analog-2	25	0	145
	Represent	CGATTCAATA				Represent	112	145	0

**79**	Analog-1	CGCCGTCAAC	0.499574821	0.797450247	0.735721595	Analog-1	0	38	184
	Analog-2	CCCCGCCAAC				Analog-2	38	0	295
	Represent	CGCCGCCAAC				Represent	184	295	0
**80**	Analog-1	GTATTACGAG	0.763935336	0.833642344	0.940489715	Analog-1	0	175	205
	Analog-2	GTATCACGAT				Analog-2	175	0	694
	Represent	GTATCACGAG				Represent	205	694	0
**81**	Analog-1	GGCCCTACAA	0.371822529	0.672694772	0.749439824	Analog-1	0	20	49
	Analog-2	GTCGCTACAA				Analog-2	20	0	175
	Represent	GGCGCTACAA				Represent	49	175	0
**82**	Analog-1	GTGCGACAAG	0.810869781	0.870765661	0.937539603	Analog-1	0	274	457
	Analog-2	ATGCGATAAG				Analog-2	274	0	646
	Represent	GTGCGATAAG				Represent	457	646	0
**83**	Analog-1	ATCGTCCGGA	0.811790374	0.811790374	1	Analog-1	0	238	238
	Analog-2	GGCGTCCGGA				Analog-2	238	0	1000
	Represent	GGCGTCCGGA				Represent	238	1000	0
**84**	Analog-1	TGGGTTAACA	0.431894335	0.687616988	0.777540291	Analog-1	0	26	208
	Analog-2	TCGATTAACA				Analog-2	26	0	133
	Represent	TCGGTTAACA				Represent	208	133	0
**85**	Analog-1	TCGAAGCGTG	0.583153742	0.671500928	0.716443358	Analog-1	0	55	67
	Analog-2	TCTAAGCATG				Analog-2	55	0	349
	Represent	TCGTAGCATG				Represent	67	349	0
**86**	Analog-1	TACCCCTTGG	0.633943859	0.830682137	0.82276417	Analog-1	0	130	292
	Analog-2	TCCCCATTGG				Analog-2	130	0	292
	Represent	TCCCCCTTGG				Represent	292	292	0
**87**	Analog-1	GATTGTAATA	0.705077603	0.762490471	0.934802328	Analog-1	0	113	127
	Analog-2	GAGTGTAATC				Analog-2	113	0	670
	Represent	GAGTGTAATA				Represent	127	670	0
**88**	Analog-1	GCCAATACGC	0.676644187	0.81181926	0.8793587	Analog-1	0	115	217
	Analog-2	GCCGATACGG				Analog-2	115	0	562
	Represent	GCCGATACGC				Represent	217	562	0
**89**	Analog-1	TAGCCCTATG	0.741785979	0.800877611	0.870781058	Analog-1	0	406	640
	Analog-2	TAGCCCTTGG				Analog-2	406	0	478
	Represent	TAGCCCTAGG				Represent	640	478	0
**90**	Analog-1	ACTGAAATCC	0.582867182	0.758667525	0.816023781	Analog-1	0	111	304
	Analog-2	AGTGAAATCG				Analog-2	111	0	448
	Represent	AGTGAAATCC				Represent	304	448	0
**91**	Analog-1	GCTCCTGCAT	0.590143329	0.744326505	0.760572359	Analog-1	0	121	448
	Analog-2	GGCCCTGCAT				Analog-2	121	0	169
	Represent	GGTCCTGCAT				Represent	448	169	0
**92**	Analog-1	CAGTGGAAAA	0.637894037	0.698799684	0.937492574	Analog-1	0	133	175
	Analog-2	CCGTGGAAAC				Analog-2	133	0	739
	Represent	CCGTGGAAAA				Represent	175	739	0
**93**	Analog-1	GAGTTTAATT	0.540038399	0.647676471	0.647492078	Analog-1	0	34	37
	Analog-2	GAACTTAATT				Analog-2	34	0	40
	Represent	GAGCTTAATT				Represent	37	40	0
**94**	Analog-1	CCAAATTGTT	0.666893484	0.819572555	0.747724109	Analog-1	0	82	298
	Analog-2	CCAAGCTGTT				Analog-2	82	0	124
	Represent	CCAAACTGTT				Represent	298	124	0

**95**	Analog-1	GGTGGCAGTG	0.579655289	0.76178867	0.841402958	Analog-1	0	115	397
	Analog-2	GCTAGCAGTG				Analog-2	115	0	247
	Represent	GCTGGCAGTG				Represent	397	247	0
**96**	Analog-1	CGCCGTGGCC	0.634415934	0.809977367	0.822453877	Analog-1	0	64	379
	Analog-2	CGCCATGTCC				Analog-2	64	0	280
	Represent	CGCCGTGTCC				Represent	379	280	0
**97**	Analog-1	GTGGGAATGG	0.642852272	0.720463302	0.831172762	Analog-1	0	124	79
	Analog-2	GTATGAATGG				Analog-2	124	0	199
	Represent	GTGTGAATGG				Represent	79	199	0
**98**	Analog-1	GACCCTGTAT	0.639464421	0.716089353	0.927925128	Analog-1	0	58	67
	Analog-2	CACCCCGTAT				Analog-2	58	0	604
	Represent	GACCCCGTAT				Represent	67	604	0
**99**	Analog-1	CCATTGCTGG	0.705096438	0.806981028	0.90728229	Analog-1	0	145	208
	Analog-2	CCATGGCTGT				Analog-2	145	0	631
	Represent	CCATGGCTGG				Represent	208	631	0
**100**	Analog-1	CATCCTCAAT	0.748307264	0.815074065	0.926967219	Analog-1	0	469	454
	Analog-2	CTTCCTCAAC				Analog-2	469	0	730
	Represent	CTTCCTCAAT				Represent	454	730	0

Supplementary 6. The random sets of five Analogs (8 based, 3 mutations), and the calculation of the RS.

		Sequence	Pearson's correlation coefficient	Origin	Represent	Analog 1	Analog 2	Analog 3	Analog 4	Analog 5
1	**Origin**	ACTTGGGG	**Origin**	1.00000	0.39839	0.37005	0.45760	0.58444	0.33905	0.49930
	**Represent**	CCTTCGGC	**Represent**	0.39839	1.00000	0.53386	0.32958	0.33666	0.74107	0.30759
	**Analog 1**	CCTTGTGC	**Analog 1**	0.37005	0.53386	1.00000	0.12570	0.18014	0.24169	0.08934
	**Analog 2**	ACTCTGGT	**Analog 2**	0.45760	0.32958	0.12570	1.00000	0.49203	0.31903	0.41290
	**Analog 3**	TCTAGGGT	**Analog 3**	0.58444	0.33666	0.18014	0.49203	1.00000	0.23101	0.24867
	**Analog 4**	ACATCGGC	**Analog 4**	0.33905	0.74107	0.24169	0.31903	0.23101	1.00000	0.22411
	**Analog 5**	GATTTGGG	**Analog 5**	0.49930	0.30759	0.08934	0.41290	0.24867	0.22411	1.00000
2	**Origin**	CCGCGATA	**Origin**	1.00000	0.32996	0.41112	0.40839	0.45551	0.41944	0.27843
	**Represent**	TCGAGGTA	**Represent**	0.32996	1.00000	0.30197	0.36436	0.28112	0.64511	0.33883
	**Analog 1**	CCGGGTAA	**Analog 1**	0.41112	0.30197	1.00000	0.30764	0.39036	0.31858	-0.05261
	**Analog 2**	ACGTAATA	**Analog 2**	0.40839	0.36436	0.30764	1.00000	0.59222	0.54858	-0.06690
	**Analog 3**	CCGTCATG	**Analog 3**	0.45551	0.28112	0.39036	0.59222	1.00000	0.43813	-0.09175
	**Analog 4**	TCGATATA	**Analog 4**	0.41944	0.64511	0.31858	0.54858	0.43813	1.00000	-0.06351
	**Analog 5**	CATCGGTA	**Analog 5**	0.27843	0.33883	-0.05261	-0.06690	-0.09175	-0.06351	1.00000
3	**Origin**	GTTTGTCT	**Origin**	1.00000	0.06547	0.27535	0.27657	0.35354	0.28337	0.34862
	**Represent**	GAGTCGCT	**Represent**	0.06547	1.00000	0.24838	0.73039	0.26683	0.59643	0.14498
	**Analog 1**	GATACTCT	**Analog 1**	0.27535	0.24838	1.00000	0.14689	0.27387	0.33002	0.11657
	**Analog 2**	ATTTCGCT	**Analog 2**	0.27657	0.73039	0.14689	1.00000	0.18941	0.76061	0.02752
	**Analog 3**	ATGTATCT	**Analog 3**	0.35354	0.26683	0.27387	0.18941	1.00000	0.09972	0.59975
	**Analog 4**	GTTACGCT	**Analog 4**	0.28337	0.59643	0.33002	0.76061	0.09972	1.00000	0.11027
	**Analog 5**	GTGTTTCA	**Analog 5**	0.34862	0.14498	0.11657	0.02752	0.59975	0.11027	1.00000
4	**Origin**	GCGACAAA	**Origin**	1.00000	0.31752	0.64755	0.45731	0.19761	0.28269	0.12449
	**Represent**	CCGAGCCA	**Represent**	0.31752	1.00000	0.30179	0.36188	0.36414	0.20215	0.05627
	**Analog 1**	TCGACAGG	**Analog 1**	0.64755	0.30179	1.00000	0.44876	0.19833	0.13182	-0.03234
	**Analog 2**	CCGTCATA	**Analog 2**	0.45731	0.36188	0.44876	1.00000	-0.01170	-0.01366	-0.09059
	**Analog 3**	GGGACCCA	**Analog 3**	0.19761	0.36414	0.19833	-0.01170	1.00000	0.30444	-0.08140
	**Analog 4**	AGGAGAAA	**Analog 4**	0.28269	0.20215	0.13182	-0.01366	0.30444	1.00000	0.44614
	**Analog 5**	GTCAGAAA	**Analog 5**	0.12449	0.05627	-0.03234	-0.09059	-0.08140	0.44614	1.00000
5	**Origin**	GCGATAGA	**Origin**	1.00000	0.84893	0.54842	0.22956	0.34384	0.35230	0.30338
	**Represent**	GCGATAGC	**Represent**	0.84893	1.00000	0.51431	0.48575	0.31464	0.24483	0.18992
	**Analog 1**	CCGAGAGG	**Analog 1**	0.54842	0.51431	1.00000	0.12940	0.00127	0.25138	0.18615
	**Analog 2**	GTAATAGC	**Analog 2**	0.22956	0.48575	0.12940	1.00000	-0.01779	0.14993	0.17057
	**Analog 3**	GCTCTATA	**Analog 3**	0.34384	0.31464	0.00127	-0.01779	1.00000	-0.09173	0.16127
	**Analog 4**	AGGACAGA	**Analog 4**	0.35230	0.24483	0.25138	0.14993	-0.09173	1.00000	0.57020
	**Analog 5**	TCTACAGA	**Analog 5**	0.30338	0.18992	0.18615	0.17057	0.16127	0.57020	1.00000
6	**Origin**	CATATTTA	**Origin**	1.00000	-0.04695	0.29606	0.49015	0.16426	0.21605	0.17764

	**Represent**	CCTCCGTG	**Represent**	-0.04695	1.00000	0.37208	0.09849	0.29955	0.17582	0.28590
	**Analog 1**	GATCCTTA	**Analog 1**	0.29606	0.37208	1.00000	0.16675	-0.04286	0.10238	-0.03804
	**Analog 2**	CAACTTTG	**Analog 2**	0.49015	0.09849	0.16675	1.00000	-0.02171	0.03247	0.15015
	**Analog 3**	CCTATCTT	**Analog 3**	0.16426	0.29955	-0.04286	-0.02171	1.00000	0.02640	0.14821
	**Analog 4**	TATTTGTA	**Analog 4**	0.21605	0.17582	0.10238	0.03247	0.02640	1.00000	0.48465
	**Analog 5**	CTTATGTG	**Analog 5**	0.17764	0.28590	-0.03804	0.15015	0.14821	0.48465	1.00000
**7**	**Origin**	ACGCCAGA	**Origin**	1.00000	0.19975	0.27629	0.37566	0.30273	0.41208	0.57552
	**Represent**	CCCCCGGC	**Represent**	0.19975	1.00000	0.60157	0.51817	0.13596	0.35420	0.31147
	**Analog 1**	ACCCCCGC	**Analog 1**	0.27629	0.60157	1.00000	0.53025	0.13428	0.11955	0.21052
	**Analog 2**	AACCCAGC	**Analog 2**	0.37566	0.51817	0.53025	1.00000	0.32600	0.15273	0.28574
	**Analog 3**	AGACCAAA	**Analog 3**	0.30273	0.13596	0.13428	0.32600	1.00000	0.18545	-0.02353
	**Analog 4**	ATGCCGTA	**Analog 4**	0.41208	0.35420	0.11955	0.15273	0.18545	1.00000	0.20793
	**Analog 5**	CCGCAAGC	**Analog 5**	0.57552	0.31147	0.21052	0.28574	-0.02353	0.20793	1.00000
**8**	**Origin**	CGATTAAA	**Origin**	1.00000	-0.00884	0.21096	0.08036	0.27626	0.33032	0.50847
	**Represent**	AGTCTCCA	**Represent**	-0.00884	1.00000	0.12023	-0.13087	0.44785	0.12358	0.10748
	**Analog 1**	ACATTACA	**Analog 1**	0.21096	0.12023	1.00000	0.09018	-0.02963	0.17176	0.25760
	**Analog 2**	CAATCATA	**Analog 2**	0.08036	-0.13087	0.09018	1.00000	-0.08523	0.19055	0.22205
	**Analog 3**	GGTCTAAA	**Analog 3**	0.27626	0.44785	-0.02963	-0.08523	1.00000	0.07072	0.06512
	**Analog 4**	TAATTCAA	**Analog 4**	0.33032	0.12358	0.17176	0.19055	0.07072	1.00000	0.21918
	**Analog 5**	AGATTATT	**Analog 5**	0.50847	0.10748	0.25760	0.22205	0.06512	0.21918	1.00000
**9**	**Origin**	AAGGCCGA	**Origin**	1.00000	0.35676	0.45557	0.08643	0.52399	0.38265	0.45143
	**Represent**	GCGGGCGG	**Represent**	0.35676	1.00000	0.55132	0.34694	0.31953	0.30501	0.22461
	**Analog 1**	GCGTCCGA	**Analog 1**	0.45557	0.55132	1.00000	-0.05075	0.40735	-0.00040	0.62265
	**Analog 2**	AATGGCCA	**Analog 2**	0.08643	0.34694	-0.05075	1.00000	0.11061	0.02677	-0.04580
	**Analog 3**	AATACCGG	**Analog 3**	0.52399	0.31953	0.40735	0.11061	1.00000	0.22899	0.48359
	**Analog 4**	AAGGATGG	**Analog 4**	0.38265	0.30501	-0.00040	0.02677	0.22899	1.00000	0.00386
	**Analog 5**	ATGTCCGT	**Analog 5**	0.45143	0.22461	0.62265	-0.04580	0.48359	0.00386	1.00000
**10**	**Origin**	GTTGTAGG	**Origin**	1.00000	0.29802	0.45916	0.19290	0.18047	0.41263	0.49145
	**Represent**	GTCGAAGC	**Represent**	0.29802	1.00000	0.39842	0.38044	0.38256	0.44643	0.39445
	**Analog 1**	GTTAAAGA	**Analog 1**	0.45916	0.39842	1.00000	-0.03372	0.30549	0.17850	0.46297
	**Analog 2**	TTCGTACG	**Analog 2**	0.19290	0.38044	-0.03372	1.00000	0.02788	0.25703	0.03761
	**Analog 3**	GTAGAAAG	**Analog 3**	0.18047	0.38256	0.30549	0.02788	1.00000	0.21399	0.51394
	**Analog 4**	TTAGTAGC	**Analog 4**	0.41263	0.44643	0.17850	0.25703	0.21399	1.00000	0.03185
	**Analog 5**	GTTGAATA	**Analog 5**	0.49145	0.39445	0.46297	0.03761	0.51394	0.03185	1.00000
**11**	**Origin**	CGAGCATG	**Origin**	1.00000	0.41080	0.50548	0.52048	0.31836	0.31468	0.45677
	**Represent**	CGCGCTCG	**Represent**	0.41080	1.00000	0.19164	0.18745	0.36474	0.51745	0.61231
	**Analog 1**	GTAGCAGG	**Analog 1**	0.50548	0.19164	1.00000	0.28965	0.60584	-0.03001	0.35961
	**Analog 2**	CGATCAGA	**Analog 2**	0.52048	0.18745	0.28965	1.00000	0.19053	0.34089	0.14863
	**Analog 3**	CCCGCAGG	**Analog 3**	0.31836	0.36474	0.60584	0.19053	1.00000	-0.07627	0.52725
	**Analog 4**	CGACCTCG	**Analog 4**	0.31468	0.51745	-0.03001	0.34089	-0.07627	1.00000	-0.00367

	**Analog 5**	GGCGCATT	**Analog 5**	0.45677	0.61231	0.35961	0.14863	0.52725	-0.00367	1.00000
**12**	**Origin**	CGTATAAA	**Origin**	1.00000	-0.03717	0.37391	0.19178	0.45934	0.11123	0.41175
	**Represent**	GGCGCCGA	**Represent**	-0.03717	1.00000	0.13806	0.04027	0.00390	0.15015	0.39363
	**Analog 1**	GGTATCAC	**Analog 1**	0.37391	0.13806	1.00000	0.09206	0.44909	0.23782	0.00096
	**Analog 2**	AATATAGA	**Analog 2**	0.19178	0.04027	0.09206	1.00000	0.02184	0.10664	-0.08158
	**Analog 3**	CGAATCAT	**Analog 3**	0.45934	0.00390	0.44909	0.02184	1.00000	0.16997	0.21367
	**Analog 4**	CATACCAA	**Analog 4**	0.11123	0.15015	0.23782	0.10664	0.16997	1.00000	-0.02969
	**Analog 5**	CGCGGAAA	**Analog 5**	0.41175	0.39363	0.00096	-0.08158	0.21367	-0.02969	1.00000
**13**	**Origin**	AAACAACG	**Origin**	1.00000	0.41126	0.27859	0.45433	0.33519	0.44100	0.58182
	**Represent**	GACCAGCG	**Represent**	0.41126	1.00000	0.54353	0.25470	0.49157	0.49476	0.34891
	**Analog 1**	AATCAGCC	**Analog 1**	0.27859	0.54353	1.00000	0.16996	0.56021	0.20491	0.01734
	**Analog 2**	GAACATAG	**Analog 2**	0.45433	0.25470	0.16996	1.00000	0.31537	0.19357	0.20065
	**Analog 3**	AGACAGCT	**Analog 3**	0.33519	0.49157	0.56021	0.31537	1.00000	0.23984	0.01353
	**Analog 4**	CACCAACT	**Analog 4**	0.44100	0.49476	0.20491	0.19357	0.23984	1.00000	0.17762
	**Analog 5**	GAAATACG	**Analog 5**	0.58182	0.34891	0.01734	0.20065	0.01353	0.17762	1.00000
**14**	**Origin**	GGTCTCCC	**Origin**	1.00000	0.49092	0.57409	0.14938	0.44730	0.51736	0.55356
	**Represent**	CGTCTCAC	**Represent**	0.49092	1.00000	0.13169	0.38145	0.23768	0.76740	0.15583
	**Analog 1**	TATTTCCC	**Analog 1**	0.57409	0.13169	1.00000	0.06391	0.18087	0.14254	0.46694
	**Analog 2**	GCGCTCAC	**Analog 2**	0.14938	0.38145	0.06391	1.00000	-0.06876	0.15938	-0.07307
	**Analog 3**	AGTCCTCC	**Analog 3**	0.44730	0.23768	0.18087	-0.06876	1.00000	0.25492	0.32787
	**Analog 4**	CGTCTCTT	**Analog 4**	0.51736	0.76740	0.14254	0.15938	0.25492	1.00000	0.16715
	**Analog 5**	TGTAACCC	**Analog 5**	0.55356	0.15583	0.46694	-0.07307	0.32787	0.16715	1.00000
**15**	**Origin**	CTCGAGCG	**Origin**	1.00000	0.63692	0.34199	0.54006	0.14612	0.44408	0.33293
	**Represent**	CTCGAGTG	**Represent**	0.63692	1.00000	0.57018	0.27083	0.43372	0.24298	0.38396
	**Analog 1**	CTCAAGTT	**Analog 1**	0.34199	0.57018	1.00000	0.14194	0.38475	-0.00757	0.43975
	**Analog 2**	GTAGAGCT	**Analog 2**	0.54006	0.27083	0.14194	1.00000	0.11035	0.34287	0.13750
	**Analog 3**	CTGCAGTG	**Analog 3**	0.14612	0.43372	0.38475	0.11035	1.00000	-0.07610	0.20956
	**Analog 4**	CACGCGCT	**Analog 4**	0.44408	0.24298	-0.00757	0.34287	-0.07610	1.00000	-0.00070
	**Analog 5**	CTCTAGGA	**Analog 5**	0.33293	0.38396	0.43975	0.13750	0.20956	-0.00070	1.00000
**16**	**Origin**	CCCCGACA	**Origin**	1.00000	0.05676	0.23243	0.20409	0.51148	0.47211	0.41811
	**Represent**	CGCACCCA	**Represent**	0.05676	1.00000	0.30795	0.37711	-0.01474	-0.01399	0.34499
	**Analog 1**	CCGCCCCA	**Analog 1**	0.23243	0.30795	1.00000	-0.08519	0.13522	0.16727	0.24440
	**Analog 2**	CGCGGACG	**Analog 2**	0.20409	0.37711	-0.08519	1.00000	0.12346	-0.05981	-0.01384
	**Analog 3**	CCCTTACT	**Analog 3**	0.51148	-0.01474	0.13522	0.12346	1.00000	0.35496	0.41526
	**Analog 4**	CCCCACAA	**Analog 4**	0.47211	-0.01399	0.16727	-0.05981	0.35496	1.00000	0.30884
	**Analog 5**	CCCACTCA	**Analog 5**	0.41811	0.34499	0.24440	-0.01384	0.41526	0.30884	1.00000
**17**	**Origin**	CCAAAACA	**Origin**	1.00000	0.15866	0.20576	0.39689	0.28328	0.33088	0.34966
	**Represent**	GCGTAGCA	**Represent**	0.15866	1.00000	0.20334	0.32741	0.41759	0.44597	0.05024
	**Analog 1**	GCCAAAGA	**Analog 1**	0.20576	0.20334	1.00000	0.19977	0.32728	0.00856	0.09322
	**Analog 2**	TCGAAACT	**Analog 2**	0.39689	0.32741	0.19977	1.00000	0.42295	0.07886	0.25319

	**Analog 3**	CCGTAAGA	**Analog 3**	0.28328	0.41759	0.32728	0.42295	1.00000	-0.09648	0.17195
	**Analog 4**	AGAAAGCA	**Analog 4**	0.33088	0.44597	0.00856	0.07886	-0.09648	1.00000	0.08767
	**Analog 5**	CCTAACCT	**Analog 5**	0.34966	0.05024	0.09322	0.25319	0.17195	0.08767	1.00000
**18**	**Origin**	GCAAGGCC	**Origin**	1.00000	0.80081	0.38430	0.39317	0.55249	0.51808	0.49102
	**Represent**	GCCAGGCC	**Represent**	0.80081	1.00000	0.32467	0.65087	0.33312	0.37016	0.46988
	**Analog 1**	GGAGAGCC	**Analog 1**	0.38430	0.32467	1.00000	0.12371	-0.00888	0.51034	-0.06517
	**Analog 2**	GCCAATCC	**Analog 2**	0.39317	0.65087	0.12371	1.00000	-0.00672	0.18709	0.22132
	**Analog 3**	TCAAGGTA	**Analog 3**	0.55249	0.33312	-0.00888	-0.00672	1.00000	0.16137	0.36622
	**Analog 4**	CCACAGCC	**Analog 4**	0.51808	0.37016	0.51034	0.18709	0.16137	1.00000	0.00572
	**Analog 5**	GCGAGGAT	**Analog 5**	0.49102	0.46988	-0.06517	0.22132	0.36622	0.00572	1.00000
**19**	**Origin**	TTGACAAC	**Origin**	1.00000	0.57246	0.14487	0.37857	0.30221	0.25560	0.41245
	**Represent**	TGGACAAG	**Represent**	0.57246	1.00000	0.44409	0.15237	0.26645	0.33126	0.25642
	**Analog 1**	TGGCCACC	**Analog 1**	0.14487	0.44409	1.00000	0.26483	0.05808	-0.01176	0.10392
	**Analog 2**	TTGGCACA	**Analog 2**	0.37857	0.15237	0.26483	1.00000	0.47194	-0.01615	0.24076
	**Analog 3**	TTCGCAAG	**Analog 3**	0.30221	0.26645	0.05808	0.47194	1.00000	0.08761	0.34687
	**Analog 4**	TCGAGAAG	**Analog 4**	0.25560	0.33126	-0.01176	-0.01615	0.08761	1.00000	0.04005
	**Analog 5**	TTTTCAAA	**Analog 5**	0.41245	0.25642	0.10392	0.24076	0.34687	0.04005	1.00000
**20**	**Origin**	GTTACTAA	**Origin**	1.00000	0.23103	0.32536	0.30403	0.37252	0.42930	0.26904
	**Represent**	GTCGCGAA	**Represent**	0.23103	1.00000	0.08379	0.34663	0.25320	0.14635	0.17432
	**Analog 1**	GTACCTGA	**Analog 1**	0.32536	0.08379	1.00000	0.08876	0.15564	0.15052	-0.09010
	**Analog 2**	TCTGCTAA	**Analog 2**	0.30403	0.34663	0.08876	1.00000	0.23971	0.09277	0.02532
	**Analog 3**	ATCTCTAA	**Analog 3**	0.37252	0.25320	0.15564	0.23971	1.00000	0.38054	0.05664
	**Analog 4**	TTCACTTA	**Analog 4**	0.42930	0.14635	0.15052	0.09277	0.38054	1.00000	-0.02412
	**Analog 5**	CTTAGGAA	**Analog 5**	0.26904	0.17432	-0.09010	0.02532	0.05664	-0.02412	1.00000
**21**	**Origin**	TGTGCAAA	**Origin**	1.00000	0.33842	0.34325	0.18326	0.33065	0.38292	0.22161
	**Represent**	AGTGAACA	**Represent**	0.33842	1.00000	0.35741	0.33639	0.41393	0.46195	0.03918
	**Analog 1**	GGTGTAGA	**Analog 1**	0.34325	0.35741	1.00000	-0.02719	0.18140	0.19668	0.45083
	**Analog 2**	TATTCACA	**Analog 2**	0.18326	0.33639	-0.02719	1.00000	-0.02183	0.07860	-0.07662
	**Analog 3**	AGTCAAAA	**Analog 3**	0.33065	0.41393	0.18140	-0.02183	1.00000	0.34578	0.18248
	**Analog 4**	AATGAAAA	**Analog 4**	0.38292	0.46195	0.19668	0.07860	0.34578	1.00000	0.20488
	**Analog 5**	GGAGTAAA	**Analog 5**	0.22161	0.03918	0.45083	-0.07662	0.18248	0.20488	1.00000
**22**	**Origin**	GCACCCGC	**Origin**	1.00000	0.75277	0.60369	0.31475	0.23898	0.36401	0.41495
	**Represent**	GCGCCCGC	**Represent**	0.75277	1.00000	0.54938	0.65360	0.10468	0.34041	0.33596
	**Analog 1**	GCTAACGC	**Analog 1**	0.60369	0.54938	1.00000	0.00203	-0.12255	0.27512	0.44681
	**Analog 2**	ACGCCCTC	**Analog 2**	0.31475	0.65360	0.00203	1.00000	0.14468	0.31626	-0.06569
	**Analog 3**	GTACCACC	**Analog 3**	0.23898	0.10468	-0.12255	0.14468	1.00000	0.32653	-0.09301
	**Analog 4**	GCTCCATC	**Analog 4**	0.36401	0.34041	0.27512	0.31626	0.32653	1.00000	-0.12087
	**Analog 5**	GGATTCGC	**Analog 5**	0.41495	0.33596	0.44681	-0.06569	-0.09301	-0.12087	1.00000
**23**	**Origin**	AGGGGAGT	**Origin**	1.00000	0.21150	0.13472	0.58100	0.30532	0.47652	0.47370
	**Represent**	CCGCGAGG	**Represent**	0.21150	1.00000	0.45397	0.15389	0.03396	0.46900	0.09703

	**Analog 1**	ATGCGGGT	**Analog 1**	0.13472	0.45397	1.00000	0.04457	0.00437	0.00522	0.08997
	**Analog 2**	TGGGGTGG	**Analog 2**	0.58100	0.15389	0.04457	1.00000	0.16072	0.33430	0.22028
	**Analog 3**	ATCGGATT	**Analog 3**	0.30532	0.03396	0.00437	0.16072	1.00000	0.41649	-0.00589
	**Analog 4**	CCGGGATT	**Analog 4**	0.47652	0.46900	0.00522	0.33430	0.41649	1.00000	0.21479
	**Analog 5**	CAGGTAGT	**Analog 5**	0.47370	0.09703	0.08997	0.22028	-0.00589	0.21479	1.00000
**24**	**Origin**	GATGAAGC	**Origin**	1.00000	0.51510	0.36165	0.17793	0.43243	0.18096	0.51654
	**Represent**	CAGAAAGC	**Represent**	0.51510	1.00000	0.31896	0.22526	0.18732	0.45207	0.41748
	**Analog 1**	GCTTGAGC	**Analog 1**	0.36165	0.31896	1.00000	0.06389	-0.04421	-0.08147	0.62379
	**Analog 2**	CATTAACC	**Analog 2**	0.17793	0.22526	0.06389	1.00000	0.54289	0.53959	0.01294
	**Analog 3**	CATGAACG	**Analog 3**	0.43243	0.18732	-0.04421	0.54289	1.00000	0.28264	-0.03856
	**Analog 4**	GAGAAACC	**Analog 4**	0.18096	0.45207	-0.08147	0.53959	0.28264	1.00000	0.07957
	**Analog 5**	GAATGAGC	**Analog 5**	0.51654	0.41748	0.62379	0.01294	-0.03856	0.07957	1.00000
**25**	**Origin**	GCATGCGG	**Origin**	1.00000	0.68348	0.36535	0.52105	0.49649	0.16148	0.68705
	**Represent**	GCACGCGC	**Represent**	0.68348	1.00000	0.18045	0.61990	0.39176	0.32869	0.48292
	**Analog 1**	TCATGTGT	**Analog 1**	0.36535	0.18045	1.00000	0.13761	0.48166	0.04297	0.24654
	**Analog 2**	GCAACCGC	**Analog 2**	0.52105	0.61990	0.13761	1.00000	0.43328	-0.02234	0.25853
	**Analog 3**	TCATACGT	**Analog 3**	0.49649	0.39176	0.48166	0.43328	1.00000	-0.01165	0.40158
	**Analog 4**	GAACGGGG	**Analog 4**	0.16148	0.32869	0.04297	-0.02234	-0.01165	1.00000	0.04492
	**Analog 5**	TTATGCGA	**Analog 5**	0.68705	0.48292	0.24654	0.25853	0.40158	0.04492	1.00000
**26**	**Origin**	CTATAAGT	**Origin**	1.00000	0.12914	0.27201	0.30602	0.28056	0.35488	0.45445
	**Represent**	TCTTCGGT	**Represent**	0.12914	1.00000	-0.09270	0.75382	-0.08450	0.24625	0.13833
	**Analog 1**	CTATTCTT	**Analog 1**	0.27201	-0.09270	1.00000	0.06222	0.21417	-0.08908	0.01450
	**Analog 2**	TTATCGGT	**Analog 2**	0.30602	0.75382	0.06222	1.00000	-0.09039	-0.01794	0.07939
	**Analog 3**	CTTCAATT	**Analog 3**	0.28056	-0.08450	0.21417	-0.09039	1.00000	0.10914	0.04698
	**Analog 4**	CCTTAAGA	**Analog 4**	0.35488	0.24625	-0.08908	-0.01794	0.10914	1.00000	0.36015
	**Analog 5**	TCATAAGC	**Analog 5**	0.45445	0.13833	0.01450	0.07939	0.04698	0.36015	1.00000
**27**	**Origin**	ATTTCTAC	**Origin**	1.00000	0.29983	0.32313	0.37261	0.41293	0.38409	0.17167
	**Represent**	ATTCGAAC	**Represent**	0.29983	1.00000	0.18655	0.17006	0.12004	0.20204	0.59928
	**Analog 1**	ATTAATAG	**Analog 1**	0.32313	0.18655	1.00000	0.31738	0.63797	-0.02282	0.03635
	**Analog 2**	ATTTAGAA	**Analog 2**	0.37261	0.17006	0.31738	1.00000	0.43320	0.02433	-0.02537
	**Analog 3**	TTTTATAG	**Analog 3**	0.41293	0.12004	0.63797	0.43320	1.00000	0.13190	0.04456
	**Analog 4**	TTGTCAAC	**Analog 4**	0.38409	0.20204	-0.02282	0.02433	0.13190	1.00000	0.03060
	**Analog 5**	AGTCGTAC	**Analog 5**	0.17167	0.59928	0.03635	-0.02537	0.04456	0.03060	1.00000
**28**	**Origin**	GACTTTTA	**Origin**	1.00000	0.29228	0.52664	0.29027	0.59512	0.44965	0.48296
	**Represent**	GACGGATG	**Represent**	0.29228	1.00000	0.27834	0.49869	0.42063	0.28748	0.25723
	**Analog 1**	AACTCTTG	**Analog 1**	0.52664	0.27834	1.00000	0.30867	0.57910	0.30227	0.48680
	**Analog 2**	GACGCTAA	**Analog 2**	0.29027	0.49869	0.30867	1.00000	0.14394	0.17811	0.37969
	**Analog 3**	TACTTATG	**Analog 3**	0.59512	0.42063	0.57910	0.14394	1.00000	0.35605	0.39907
	**Analog 4**	GACTGCTT	**Analog 4**	0.44965	0.28748	0.30227	0.17811	0.35605	1.00000	0.36349
	**Analog 5**	GACTCAAA	**Analog 5**	0.48296	0.25723	0.48680	0.37969	0.39907	0.36349	1.00000

**29**	**Origin**	CCCTCTGC	**Origin**	1.00000	0.36829	0.35837	0.24957	0.35734	0.23714	0.42849
	**Represent**	ACATCCGC	**Represent**	0.36829	1.00000	0.71499	0.39088	-0.02878	0.53119	0.24490
	**Analog 1**	CCACCCGC	**Analog 1**	0.35837	0.71499	1.00000	0.11630	-0.06738	0.37923	0.22233
	**Analog 2**	ACATCTCC	**Analog 2**	0.24957	0.39088	0.11630	1.00000	0.03140	0.05572	-0.01131
	**Analog 3**	TCCTTTTC	**Analog 3**	0.35734	-0.02878	-0.06738	0.03140	1.00000	0.06713	0.17488
	**Analog 4**	CACTCCGA	**Analog 4**	0.23714	0.53119	0.37923	0.05572	0.06713	1.00000	0.03499
	**Analog 5**	GCCACAGC	**Analog 5**	0.42849	0.24490	0.22233	-0.01131	0.17488	0.03499	1.00000
**30**	**Origin**	TCGCCCGT	**Origin**	1.00000	0.39383	0.39577	0.49805	0.58911	0.47662	0.28660
	**Represent**	TGGCCTGG	**Represent**	0.39383	1.00000	0.82617	0.42821	0.03967	0.22285	0.44455
	**Analog 1**	TGGCCTAT	**Analog 1**	0.39577	0.82617	1.00000	0.28853	0.00088	0.20017	0.31782
	**Analog 2**	TGTCCCGG	**Analog 2**	0.49805	0.42821	0.28853	1.00000	0.35611	0.43311	0.34144
	**Analog 3**	CCGACCGA	**Analog 3**	0.58911	0.03967	0.00088	0.35611	1.00000	0.61142	-0.01144
	**Analog 4**	AGGACCGT	**Analog 4**	0.47662	0.22285	0.20017	0.43311	0.61142	1.00000	0.10430
	**Analog 5**	TTTCCTGT	**Analog 5**	0.28660	0.44455	0.31782	0.34144	-0.01144	0.10430	1.00000
**31**	**Origin**	AGGACTCA	**Origin**	1.00000	0.65832	0.52139	0.46070	0.27684	0.33553	0.30873
	**Represent**	GGGATTCA	**Represent**	0.65832	1.00000	0.53785	0.62437	0.36562	0.52268	0.48467
	**Analog 1**	GGGACACT	**Analog 1**	0.52139	0.53785	1.00000	0.14496	0.13402	-0.01384	-0.02245
	**Analog 2**	TTGATTCA	**Analog 2**	0.46070	0.62437	0.14496	1.00000	0.60785	0.63816	0.55347
	**Analog 3**	ATGATCCA	**Analog 3**	0.27684	0.36562	0.13402	0.60785	1.00000	0.38275	0.25324
	**Analog 4**	ATAATTCA	**Analog 4**	0.33553	0.52268	-0.01384	0.63816	0.38275	1.00000	0.79570
	**Analog 5**	AAAATTCA	**Analog 5**	0.30873	0.48467	-0.02245	0.55347	0.25324	0.79570	1.00000
**32**	**Origin**	CCAGCTGT	**Origin**	1.00000	0.35231	0.34354	0.35354	0.40473	0.25256	0.50742
	**Represent**	GCGGCCGT	**Represent**	0.35231	1.00000	0.28917	0.71749	0.07342	0.25794	0.31023
	**Analog 1**	CCAAGCGT	**Analog 1**	0.34354	0.28917	1.00000	0.05383	0.17780	0.03462	0.06235
	**Analog 2**	GCGGCGGT	**Analog 2**	0.35354	0.71749	0.05383	1.00000	0.07332	0.25232	0.31160
	**Analog 3**	AGAACTGT	**Analog 3**	0.40473	0.07342	0.17780	0.07332	1.00000	0.23291	0.40340
	**Analog 4**	CAGGTTGT	**Analog 4**	0.25256	0.25794	0.03462	0.25232	0.23291	1.00000	0.10828
	**Analog 5**	AGAGCAGT	**Analog 5**	0.50742	0.31023	0.06235	0.31160	0.40340	0.10828	1.00000
**33**	**Origin**	CTTTGGCG	**Origin**	1.00000	0.24815	0.61242	0.25692	0.29504	0.36747	0.53333
	**Represent**	TGTTGTCA	**Represent**	0.24815	1.00000	0.28782	0.01709	0.09251	0.74352	0.06966
	**Analog 1**	TGTGGGCG	**Analog 1**	0.61242	0.28782	1.00000	-0.01611	0.13873	0.04471	0.41346
	**Analog 2**	CTTTCGTC	**Analog 2**	0.25692	0.01709	-0.01611	1.00000	-0.00951	0.13208	0.43942
	**Analog 3**	CAATGGGG	**Analog 3**	0.29504	0.09251	0.13873	-0.00951	1.00000	0.10825	-0.01559
	**Analog 4**	TTTTGTCA	**Analog 4**	0.36747	0.74352	0.04471	0.13208	0.10825	1.00000	0.07796
	**Analog 5**	ACTTCGCG	**Analog 5**	0.53333	0.06966	0.41346	0.43942	-0.01559	0.07796	1.00000
**34**	**Origin**	GGCACCTC	**Origin**	1.00000	0.31834	0.17460	0.55455	0.28411	0.30856	0.23478
	**Represent**	GGCGCGCC	**Represent**	0.31834	1.00000	0.03662	0.67414	0.36788	0.31036	0.15495
	**Analog 1**	GGAATCGC	**Analog 1**	0.17460	0.03662	1.00000	0.08457	-0.06376	0.10171	-0.09197
	**Analog 2**	GGCACGCA	**Analog 2**	0.55455	0.67414	0.08457	1.00000	-0.00518	0.47153	0.26147
	**Analog 3**	GACGCCTG	**Analog 3**	0.28411	0.36788	-0.06376	-0.00518	1.00000	0.07000	-0.01181

	**Analog 4**	GGGACGTG	**Analog 4**	0.30856	0.31036	0.10171	0.47153	0.07000	1.00000	0.07560
	**Analog 5**	GTCACACC	**Analog 5**	0.23478	0.15495	-0.09197	0.26147	-0.01181	0.07560	1.00000
**35**	**Origin**	CCGAAGAA	**Origin**	1.00000	0.30420	0.37490	0.19094	0.46881	0.32796	0.17660
	**Represent**	TCGTTGGA	**Represent**	0.30420	1.00000	0.17468	0.30083	0.57259	-0.07391	-0.07105
	**Analog 1**	TTGATGAA	**Analog 1**	0.37490	0.17468	1.00000	0.04007	0.08313	0.21371	-0.01882
	**Analog 2**	CTTAAGGA	**Analog 2**	0.19094	0.30083	0.04007	1.00000	-0.08369	0.39982	0.23293
	**Analog 3**	TCGTATAA	**Analog 3**	0.46881	0.57259	0.08313	-0.08369	1.00000	-0.05395	-0.09640
	**Analog 4**	CTCAAGAG	**Analog 4**	0.32796	-0.07391	0.21371	0.39982	-0.05395	1.00000	0.42800
	**Analog 5**	CACAAGTA	**Analog 5**	0.17660	-0.07105	-0.01882	0.23293	-0.09640	0.42800	1.00000
**36**	**Origin**	AGACCACC	**Origin**	1.00000	0.37480	0.48192	0.21910	0.47583	0.32278	0.33583
	**Represent**	TGCGCACC	**Represent**	0.37480	1.00000	0.11547	0.28477	0.21324	0.84118	0.17487
	**Analog 1**	TGACCCCT	**Analog 1**	0.48192	0.11547	1.00000	0.20761	0.40762	0.02691	0.28676
	**Analog 2**	AGCCGCCC	**Analog 2**	0.21910	0.28477	0.20761	1.00000	0.17369	0.24119	-0.01275
	**Analog 3**	TGACATCC	**Analog 3**	0.47583	0.21324	0.40762	0.17369	1.00000	-0.02390	0.10477
	**Analog 4**	AGCGCACA	**Analog 4**	0.32278	0.84118	0.02691	0.24119	-0.02390	1.00000	0.09548
	**Analog 5**	TGGCCAAC	**Analog 5**	0.33583	0.17487	0.28676	-0.01275	0.10477	0.09548	1.00000
**37**	**Origin**	AGAGCCTT	**Origin**	1.00000	0.11110	0.12236	0.35861	0.16937	0.29655	0.15855
	**Represent**	AGGGGCGT	**Represent**	0.11110	1.00000	0.32912	0.03086	0.14339	0.00141	0.18171
	**Analog 1**	ACAGTCGT	**Analog 1**	0.12236	0.32912	1.00000	-0.03235	0.05654	0.15132	-0.12926
	**Analog 2**	AGATTATT	**Analog 2**	0.35861	0.03086	-0.03235	1.00000	0.06252	-0.02405	0.17315
	**Analog 3**	AAAGGGTT	**Analog 3**	0.16937	0.14339	0.05654	0.06252	1.00000	0.32751	-0.03515
	**Analog 4**	AAAGACTA	**Analog 4**	0.29655	0.00141	0.15132	-0.02405	0.32751	1.00000	-0.10134
	**Analog 5**	AGGCCTTT	**Analog 5**	0.15855	0.18171	-0.12926	0.17315	-0.03515	-0.10134	1.00000
**38**	**Origin**	CCATTGCT	**Origin**	1.00000	0.72318	0.37524	0.39826	0.30105	0.50748	0.31314
	**Represent**	CCAGTGCC	**Represent**	0.72318	1.00000	0.24421	0.44023	0.42783	0.51991	0.20376
	**Analog 1**	GCGTGGCT	**Analog 1**	0.37524	0.24421	1.00000	0.18543	0.28637	0.45148	0.07299
	**Analog 2**	CCATGTCC	**Analog 2**	0.39826	0.44023	0.18543	1.00000	0.17088	0.18961	0.32376
	**Analog 3**	GCAGTACT	**Analog 3**	0.30105	0.42783	0.28637	0.17088	1.00000	0.26841	0.32408
	**Analog 4**	GCCTTGCC	**Analog 4**	0.50748	0.51991	0.45148	0.18961	0.26841	1.00000	0.04380
	**Analog 5**	ACACTTCT	**Analog 5**	0.31314	0.20376	0.07299	0.32376	0.32408	0.04380	1.00000
**39**	**Origin**	AGTACACC	**Origin**	1.00000	0.17560	0.25071	0.37135	0.33025	0.32078	0.35461
	**Represent**	CGGCCGCC	**Represent**	0.17560	1.00000	0.90810	0.21175	0.28067	0.19372	0.44501
	**Analog 1**	AGGCCGCC	**Analog 1**	0.25071	0.90810	1.00000	0.04302	0.13659	0.26650	0.46186
	**Analog 2**	CGTACCCT	**Analog 2**	0.37135	0.21175	0.04302	1.00000	0.67963	0.05757	0.39530
	**Analog 3**	CGCACCCC	**Analog 3**	0.33025	0.28067	0.13659	0.67963	1.00000	0.05345	0.16700
	**Analog 4**	AGGATACG	**Analog 4**	0.32078	0.19372	0.26650	0.05757	0.05345	1.00000	0.05208
	**Analog 5**	GGTACGCT	**Analog 5**	0.35461	0.44501	0.46186	0.39530	0.16700	0.05208	1.00000
**40**	**Origin**	ACTACTTT	**Origin**	1.00000	0.35812	0.39375	0.47294	0.19490	0.26454	0.28136
	**Represent**	ACCACCTG	**Represent**	0.35812	1.00000	0.10744	0.24910	-0.03355	0.47268	0.53114
	**Analog 1**	GATACTGT	**Analog 1**	0.39375	0.10744	1.00000	-0.03723	0.03529	0.13242	-0.07393

	**Analog 2**	ACTTTTTG	**Analog 2**	0.47294	0.24910	-0.03723	1.00000	0.05734	-0.07850	0.14909
	**Analog 3**	AAGAGTTT	**Analog 3**	0.19490	-0.03355	0.03529	0.05734	1.00000	0.22616	0.04654
	**Analog 4**	ATGACCTT	**Analog 4**	0.26454	0.47268	0.13242	-0.07850	0.22616	1.00000	0.03989
	**Analog 5**	ACCAAATT	**Analog 5**	0.28136	0.53114	-0.07393	0.14909	0.04654	0.03989	1.00000
**41**	**Origin**	GGGCCCAT	**Origin**	1.00000	0.26214	0.46813	0.24389	0.27019	0.37226	0.51232
	**Represent**	GCGGCCAC	**Represent**	0.26214	1.00000	0.47635	-0.02896	0.42188	0.29453	0.32160
	**Analog 1**	GCGCCCCG	**Analog 1**	0.46813	0.47635	1.00000	-0.07275	0.00056	0.39872	0.26932
	**Analog 2**	GGTACAAT	**Analog 2**	0.24389	-0.02896	-0.07275	1.00000	0.09563	-0.06013	0.04850
	**Analog 3**	AGGGCTAT	**Analog 3**	0.27019	0.42188	0.00056	0.09563	1.00000	-0.00024	0.02710
	**Analog 4**	TCGCGCAT	**Analog 4**	0.37226	0.29453	0.39872	-0.06013	-0.00024	1.00000	0.11452
	**Analog 5**	TGACCCAC	**Analog 5**	0.51232	0.32160	0.26932	0.04850	0.02710	0.11452	1.00000
**42**	**Origin**	CGCCACGT	**Origin**	1.00000	0.89011	0.41904	0.58478	0.51988	0.28745	0.37784
	**Represent**	CGCCACGC	**Represent**	0.89011	1.00000	0.39484	0.72393	0.43010	0.26987	0.35631
	**Analog 1**	CTCCACAA	**Analog 1**	0.41904	0.39484	1.00000	0.14919	-0.00220	0.37639	0.15557
	**Analog 2**	CGCCTAGC	**Analog 2**	0.58478	0.72393	0.14919	1.00000	0.28962	0.16682	0.23335
	**Analog 3**	AGCGTCGT	**Analog 3**	0.51988	0.43010	-0.00220	0.28962	1.00000	-0.05037	0.56791
	**Analog 4**	CACCATTT	**Analog 4**	0.28745	0.26987	0.37639	0.16682	-0.05037	1.00000	0.09621
	**Analog 5**	AGCGACTT	**Analog 5**	0.37784	0.35631	0.15557	0.23335	0.56791	0.09621	1.00000
**43**	**Origin**	TGTTAGCC	**Origin**	1.00000	0.45396	0.46861	0.42660	0.54736	0.58313	0.19257
	**Represent**	TATCCGCC	**Represent**	0.45396	1.00000	0.35048	0.19335	0.29266	0.64657	0.10379
	**Analog 1**	AGGTGGCC	**Analog 1**	0.46861	0.35048	1.00000	0.13252	0.26462	0.38767	-0.00787
	**Analog 2**	TGTACCCC	**Analog 2**	0.42660	0.19335	0.13252	1.00000	0.10077	0.45978	-0.00619
	**Analog 3**	TGACAGCT	**Analog 3**	0.54736	0.29266	0.26462	0.10077	1.00000	0.28068	0.11248
	**Analog 4**	GGTACGCC	**Analog 4**	0.58313	0.64657	0.38767	0.45978	0.28068	1.00000	-0.00993
	**Analog 5**	TATTAACT	**Analog 5**	0.19257	0.10379	-0.00787	-0.00619	0.11248	-0.00993	1.00000
**44**	**Origin**	AGATTATA	**Origin**	1.00000	0.03715	0.21426	0.38589	0.23326	0.33054	0.14159
	**Represent**	GGGTAACG	**Represent**	0.03715	1.00000	0.39192	0.40546	0.18645	0.57090	0.32557
	**Analog 1**	TGGTTAGA	**Analog 1**	0.21426	0.39192	1.00000	0.02200	0.05359	0.58585	0.22117
	**Analog 2**	AGACTACG	**Analog 2**	0.38589	0.40546	0.02200	1.00000	0.21093	0.02702	-0.08497
	**Analog 3**	GGTCTATA	**Analog 3**	0.23326	0.18645	0.05359	0.21093	1.00000	0.36838	0.05045
	**Analog 4**	GGGTTATC	**Analog 4**	0.33054	0.57090	0.58585	0.02702	0.36838	1.00000	0.32169
	**Analog 5**	ATGTAATA	**Analog 5**	0.14159	0.32557	0.22117	-0.08497	0.05045	0.32169	1.00000
**45**	**Origin**	TTCAAGGA	**Origin**	1.00000	0.56316	0.48750	0.36562	0.20101	0.10925	0.55234
	**Represent**	TTCGAGGG	**Represent**	0.56316	1.00000	0.48905	0.43450	0.03723	0.03396	0.22046
	**Analog 1**	TATGAGGA	**Analog 1**	0.48750	0.48905	1.00000	0.22825	-0.07612	-0.05691	-0.07795
	**Analog 2**	TTAAGGGG	**Analog 2**	0.36562	0.43450	0.22825	1.00000	0.05788	0.45655	0.05332
	**Analog 3**	TTGAATAA	**Analog 3**	0.20101	0.03723	-0.07612	0.05788	1.00000	0.56713	0.43847
	**Analog 4**	TTGAGGAA	**Analog 4**	0.10925	0.03396	-0.05691	0.45655	0.56713	1.00000	0.11420
	**Analog 5**	TTCAATAG	**Analog 5**	0.55234	0.22046	-0.07795	0.05332	0.43847	0.11420	1.00000
**46**	**Origin**	CACGTCAG	**Origin**	1.00000	-0.01653	0.22453	0.48096	0.40177	0.30868	0.31793

	**Represent**	CGGGCCCG	**Represent**	-0.01653	1.00000	0.32192	0.33695	0.33703	0.18013	0.15930
	**Analog 1**	CAAATCCG	**Analog 1**	0.22453	0.32192	1.00000	-0.06255	-0.06166	0.07309	-0.02971
	**Analog 2**	TTCGCCAG	**Analog 2**	0.48096	0.33695	-0.06255	1.00000	0.88241	0.16031	0.09367
	**Analog 3**	CTCGCCAA	**Analog 3**	0.40177	0.33703	-0.06166	0.88241	1.00000	0.07425	-0.00229
	**Analog 4**	CGTTTCAG	**Analog 4**	0.30868	0.18013	0.07309	0.16031	0.07425	1.00000	0.08131
	**Analog 5**	TAGGTTAG	**Analog 5**	0.31793	0.15930	-0.02971	0.09367	-0.00229	0.08131	1.00000
**47**	**Origin**	GTGTTGAC	**Origin**	1.00000	0.37110	0.37025	0.27383	0.42197	0.24434	0.14490
	**Represent**	GGGTTCAC	**Represent**	0.37110	1.00000	0.31586	0.35618	0.09238	0.52486	0.35376
	**Analog 1**	GGACTGAC	**Analog 1**	0.37025	0.31586	1.00000	0.64995	-0.12906	0.11997	0.29730
	**Analog 2**	GGATAGAC	**Analog 2**	0.27383	0.35618	0.64995	1.00000	-0.04078	0.12688	0.17588
	**Analog 3**	GTGTAACC	**Analog 3**	0.42197	0.09238	-0.12906	-0.04078	1.00000	-0.08962	-0.06168
	**Analog 4**	GATTTCAC	**Analog 4**	0.24434	0.52486	0.11997	0.12688	-0.08962	1.00000	-0.07961
	**Analog 5**	GGGGTGTC	**Analog 5**	0.14490	0.35376	0.29730	0.17588	-0.06168	-0.07961	1.00000
**48**	**Origin**	GCCAAGTA	**Origin**	1.00000	0.58696	0.47530	0.48256	0.40497	0.46605	0.26627
	**Represent**	TCCAAGGA	**Represent**	0.58696	1.00000	0.21336	0.41292	0.22300	0.24868	0.32158
	**Analog 1**	GCCTAACA	**Analog 1**	0.47530	0.21336	1.00000	0.28134	-0.08928	-0.08546	0.17797
	**Analog 2**	CCCATATA	**Analog 2**	0.48256	0.41292	0.28134	1.00000	-0.03522	0.01012	0.01367
	**Analog 3**	GATAAGTT	**Analog 3**	0.40497	0.22300	-0.08928	-0.03522	1.00000	0.57615	-0.02500
	**Analog 4**	TTAAAGTA	**Analog 4**	0.46605	0.24868	-0.08546	0.01012	0.57615	1.00000	-0.01199
	**Analog 5**	GCGAGGGA	**Analog 5**	0.26627	0.32158	0.17797	0.01367	-0.02500	-0.01199	1.00000
**49**	**Origin**	CCTGAGTC	**Origin**	1.00000	0.27229	0.43702	0.44644	0.28325	0.43665	0.53082
	**Represent**	GCCGTGTC	**Represent**	0.27229	1.00000	0.26668	0.39773	0.55625	0.47347	0.16646
	**Analog 1**	CCTGTGGG	**Analog 1**	0.43702	0.26668	1.00000	0.04004	-0.00545	0.41942	0.15805
	**Analog 2**	GCAGAGTT	**Analog 2**	0.44644	0.39773	0.04004	1.00000	0.18483	0.14764	0.35379
	**Analog 3**	TCCGAATC	**Analog 3**	0.28325	0.55625	-0.00545	0.18483	1.00000	0.08497	0.02774
	**Analog 4**	GTTGTGTC	**Analog 4**	0.43665	0.47347	0.41942	0.14764	0.08497	1.00000	0.19158
	**Analog 5**	ACTTAGTA	**Analog 5**	0.53082	0.16646	0.15805	0.35379	0.02774	0.19158	1.00000
**50**	**Origin**	AGTGCCTT	**Origin**	1.00000	0.41060	0.14739	0.35689	0.55815	0.14541	0.16289
	**Represent**	CGCGCCCT	**Represent**	0.41060	1.00000	0.21162	0.38228	0.53645	-0.00873	0.42074
	**Analog 1**	ACCGGCTT	**Analog 1**	0.14739	0.21162	1.00000	0.00814	0.04670	0.54631	0.28675
	**Analog 2**	CGAGCGTT	**Analog 2**	0.35689	0.38228	0.00814	1.00000	0.19705	-0.07497	0.22682
	**Analog 3**	CCTGCCCT	**Analog 3**	0.55815	0.53645	0.04670	0.19705	1.00000	0.25462	-0.06160
	**Analog 4**	ACTGGCAT	**Analog 4**	0.14541	-0.00873	0.54631	-0.07497	0.25462	1.00000	-0.06601
	**Analog 5**	AGCGAGTT	**Analog 5**	0.16289	0.42074	0.28675	0.22682	-0.06160	-0.06601	1.00000
**51**	**Origin**	CCTGTGTC	**Origin**	1.00000	0.39165	0.29104	0.42318	0.28850	0.27150	0.40809
	**Represent**	GCTGCGTG	**Represent**	0.39165	1.00000	0.14396	0.12328	0.77970	0.29349	0.20633
	**Analog 1**	CTTGATTC	**Analog 1**	0.29104	0.14396	1.00000	0.33956	0.36424	-0.08143	-0.01677
	**Analog 2**	CTTGTGGG	**Analog 2**	0.42318	0.12328	0.33956	1.00000	0.30540	0.36145	0.25035
	**Analog 3**	CTTGCGTG	**Analog 3**	0.28850	0.77970	0.36424	0.30540	1.00000	-0.02330	0.23456
	**Analog 4**	GCTATGGC	**Analog 4**	0.27150	0.29349	-0.08143	0.36145	-0.02330	1.00000	0.07992

	**Analog 5**	CGAGTGTG	**Analog 5**	0.40809	0.20633	-0.01677	0.25035	0.23456	0.07992	1.00000
**52**	**Origin**	GTTGCAAC	**Origin**	1.00000	0.35589	0.25921	0.25563	0.40911	0.50013	0.39740
	**Represent**	GTTCCCAC	**Represent**	0.35589	1.00000	0.24279	0.58988	0.21629	0.07464	0.51175
	**Analog 1**	GTCCGAAC	**Analog 1**	0.25921	0.24279	1.00000	0.31472	0.09433	0.04226	0.21969
	**Analog 2**	GTCACCAC	**Analog 2**	0.25563	0.58988	0.31472	1.00000	0.28560	0.07298	0.11730
	**Analog 3**	GTTACACT	**Analog 3**	0.40911	0.21629	0.09433	0.28560	1.00000	0.43345	0.18683
	**Analog 4**	GTGGCACG	**Analog 4**	0.50013	0.07464	0.04226	0.07298	0.43345	1.00000	0.09368
	**Analog 5**	AATCCAAC	**Analog 5**	0.39740	0.51175	0.21969	0.11730	0.18683	0.09368	1.00000
**53**	**Origin**	CAGTCGAT	**Origin**	1.00000	0.20214	0.42981	0.41333	0.17193	0.35280	0.49149
	**Represent**	CGCACGTT	**Represent**	0.20214	1.00000	0.34870	0.15399	0.19022	0.00636	0.45009
	**Analog 1**	CATACGGT	**Analog 1**	0.42981	0.34870	1.00000	0.22001	0.12742	-0.05555	0.27324
	**Analog 2**	TCGCCGAT	**Analog 2**	0.41333	0.15399	0.22001	1.00000	-0.07236	0.07607	0.28897
	**Analog 3**	CACTTGTT	**Analog 3**	0.17193	0.19022	0.12742	-0.07236	1.00000	-0.08333	-0.02360
	**Analog 4**	TGGTCAAT	**Analog 4**	0.35280	0.00636	-0.05555	0.07607	-0.08333	1.00000	0.18251
	**Analog 5**	CGTTCGAG	**Analog 5**	0.49149	0.45009	0.27324	0.28897	-0.02360	0.18251	1.00000
**54**	**Origin**	ATTGTAAG	**Origin**	1.00000	0.30189	0.20527	0.22603	0.23698	0.29762	0.29952
	**Represent**	AACGTCAG	**Represent**	0.30189	1.00000	0.10562	0.32577	0.63651	0.14425	0.36976
	**Analog 1**	AGAGTAGG	**Analog 1**	0.20527	0.10562	1.00000	-0.02084	-0.02205	0.38663	-0.08272
	**Analog 2**	ATCGAAAC	**Analog 2**	0.22603	0.32577	-0.02084	1.00000	0.66864	0.03396	-0.03535
	**Analog 3**	AACGAAAG	**Analog 3**	0.23698	0.63651	-0.02205	0.66864	1.00000	-0.01062	0.02924
	**Analog 4**	CTAGTATG	**Analog 4**	0.29762	0.14425	0.38663	0.03396	-0.01062	1.00000	-0.03200
	**Analog 5**	TTTCTCAG	**Analog 5**	0.29952	0.36976	-0.08272	-0.03535	0.02924	-0.03200	1.00000
**55**	**Origin**	TGCCCAGT	**Origin**	1.00000	0.39254	0.40838	0.30202	0.36779	0.43786	0.41583
	**Represent**	CACCGAGT	**Represent**	0.39254	1.00000	-0.01770	0.31535	0.06027	0.49099	0.79589
	**Analog 1**	TGCAAATT	**Analog 1**	0.40838	-0.01770	1.00000	-0.04246	0.38762	-0.05631	-0.01229
	**Analog 2**	TCCCTTGT	**Analog 2**	0.30202	0.31535	-0.04246	1.00000	0.08193	0.28888	0.33454
	**Analog 3**	GGCACGGT	**Analog 3**	0.36779	0.06027	0.38762	0.08193	1.00000	0.11527	0.06848
	**Analog 4**	CACCCCGT	**Analog 4**	0.43786	0.49099	-0.05631	0.28888	0.11527	1.00000	0.28142
	**Analog 5**	ATCCGAGT	**Analog 5**	0.41583	0.79589	-0.01229	0.33454	0.06848	0.28142	1.00000
**56**	**Origin**	CGAGATCA	**Origin**	1.00000	0.22326	0.42395	0.47858	0.12113	0.40610	0.35829
	**Represent**	AGGGATGA	**Represent**	0.22326	1.00000	0.24195	0.08920	0.27247	0.07224	0.67821
	**Analog 1**	ATAGATAA	**Analog 1**	0.42395	0.24195	1.00000	-0.02765	0.18400	0.04270	0.22015
	**Analog 2**	AGATTTCA	**Analog 2**	0.47858	0.08920	-0.02765	1.00000	0.10041	0.40177	0.25603
	**Analog 3**	CAAGTTGA	**Analog 3**	0.12113	0.27247	0.18400	0.10041	1.00000	-0.07921	-0.02943
	**Analog 4**	TGTTATCA	**Analog 4**	0.40610	0.07224	0.04270	0.40177	-0.07921	1.00000	0.24006
	**Analog 5**	AGGGATCG	**Analog 5**	0.35829	0.67821	0.22015	0.25603	-0.02943	0.24006	1.00000
**57**	**Origin**	AGATCAGT	**Origin**	1.00000	0.25281	0.58763	0.32865	0.15329	0.41197	0.53139
	**Represent**	CCGTCAAT	**Represent**	0.25281	1.00000	0.43171	0.08971	0.10097	0.07677	0.45544
	**Analog 1**	TTGTCAGT	**Analog 1**	0.58763	0.43171	1.00000	-0.08360	-0.03559	0.24619	0.50850
	**Analog 2**	AGAATAAT	**Analog 2**	0.32865	0.08971	-0.08360	1.00000	0.41463	0.09297	-0.08786

	**Analog 3**	AGCTTAAT	**Analog 3**	0.15329	0.10097	-0.03559	0.41463	1.00000	0.07448	0.12940
	**Analog 4**	AGTCCAGC	**Analog 4**	0.41197	0.07677	0.24619	0.09297	0.07448	1.00000	0.22006
	**Analog 5**	CCCTCAGT	**Analog 5**	0.53139	0.45544	0.50850	-0.08786	0.12940	0.22006	1.00000
**58**	**Origin**	CAGACGTT	**Origin**	1.00000	0.13653	0.22759	0.24282	0.34914	0.13963	0.29247
	**Represent**	AGGAAGCT	**Represent**	0.13653	1.00000	0.02906	-0.07454	0.37738	0.71913	-0.07161
	**Analog 1**	CATAACTT	**Analog 1**	0.22759	0.02906	1.00000	0.07684	0.21922	0.03383	0.31837
	**Analog 2**	CAGTCTGT	**Analog 2**	0.24282	-0.07454	0.07684	1.00000	-0.01446	-0.07855	0.55963
	**Analog 3**	AGGACCTT	**Analog 3**	0.34914	0.37738	0.21922	-0.01446	1.00000	0.06764	0.36916
	**Analog 4**	CTGAAGCT	**Analog 4**	0.13963	0.71913	0.03383	-0.07855	0.06764	1.00000	-0.07556
	**Analog 5**	CAGTCCTA	**Analog 5**	0.29247	-0.07161	0.31837	0.55963	0.36916	-0.07556	1.00000
**59**	**Origin**	GTCGGAAT	**Origin**	1.00000	0.19078	0.49668	0.33680	0.45118	0.25044	0.34885
	**Represent**	GCCCGAAA	**Represent**	0.19078	1.00000	0.21476	0.41939	0.28161	0.25104	0.50371
	**Analog 1**	CTTGGAAA	**Analog 1**	0.49668	0.21476	1.00000	0.44280	0.09278	0.40935	0.11912
	**Analog 2**	GCGGGAAA	**Analog 2**	0.33680	0.41939	0.44280	1.00000	0.13154	0.46660	0.03629
	**Analog 3**	ACCGTAAT	**Analog 3**	0.45118	0.28161	0.09278	0.13154	1.00000	0.09999	-0.02651
	**Analog 4**	GCTGGTAT	**Analog 4**	0.25044	0.25104	0.40935	0.46660	0.09999	1.00000	-0.06101
	**Analog 5**	GTCCGAGG	**Analog 5**	0.34885	0.50371	0.11912	0.03629	-0.02651	-0.06101	1.00000
**60**	**Origin**	CGCAGTTC	**Origin**	1.00000	0.45953	0.24962	0.23484	0.49854	0.47161	0.39319
	**Represent**	CGGATTTC	**Represent**	0.45953	1.00000	0.46239	0.20473	0.62845	0.06200	-0.03465
	**Analog 1**	TGGAGGTC	**Analog 1**	0.24962	0.46239	1.00000	-0.04380	0.45397	0.10880	0.06755
	**Analog 2**	CCCATTTT	**Analog 2**	0.23484	0.20473	-0.04380	1.00000	-0.05067	0.54037	0.17401
	**Analog 3**	CGGAGTAG	**Analog 3**	0.49854	0.62845	0.45397	-0.05067	1.00000	0.21700	0.23686
	**Analog 4**	TCCAGTTG	**Analog 4**	0.47161	0.06200	0.10880	0.54037	0.21700	1.00000	0.44278
	**Analog 5**	TACAGTCC	**Analog 5**	0.39319	-0.03465	0.06755	0.17401	0.23686	0.44278	1.00000
**61**	**Origin**	CGTCGAGC	**Origin**	1.00000	0.38869	0.51947	0.47388	0.55525	0.44664	0.41673
	**Represent**	CGGCAGGC	**Represent**	0.38869	1.00000	0.52403	0.36733	-0.00296	0.12458	0.73398
	**Analog 1**	CGGCGAAT	**Analog 1**	0.51947	0.52403	1.00000	0.11350	0.31947	0.38222	0.18365
	**Analog 2**	CGTGACGC	**Analog 2**	0.47388	0.36733	0.11350	1.00000	-0.01724	0.41061	0.40697
	**Analog 3**	ACTCGAGG	**Analog 3**	0.55525	-0.00296	0.31947	-0.01724	1.00000	0.10722	-0.00559
	**Analog 4**	CGTGGAAG	**Analog 4**	0.44664	0.12458	0.38222	0.41061	0.10722	1.00000	0.14182
	**Analog 5**	CGACAGGC	**Analog 5**	0.41673	0.73398	0.18365	0.40697	-0.00559	0.14182	1.00000
**62**	**Origin**	GAGTCACG	**Origin**	1.00000	-0.12836	0.49796	0.45929	0.20294	0.12646	0.33963
	**Represent**	GCTGTCAG	**Represent**	-0.12836	1.00000	0.25217	-0.12537	0.15445	0.20359	0.34572
	**Analog 1**	GATGTACG	**Analog 1**	0.49796	0.25217	1.00000	0.14320	0.18053	-0.08724	0.07002
	**Analog 2**	GTATCACC	**Analog 2**	0.45929	-0.12537	0.14320	1.00000	0.00265	-0.03494	-0.08142
	**Analog 3**	GCGGCGCG	**Analog 3**	0.20294	0.15445	0.18053	0.00265	1.00000	0.35723	-0.07901
	**Analog 4**	GCGTAATG	**Analog 4**	0.12646	0.20359	-0.08724	-0.03494	0.35723	1.00000	0.10651
	**Analog 5**	GAGTTCAG	**Analog 5**	0.33963	0.34572	0.07002	-0.08142	-0.07901	0.10651	1.00000
**63**	**Origin**	TCTATAAA	**Origin**	1.00000	0.38965	0.47282	0.29061	0.36191	0.41985	0.22558
	**Represent**	CCTATCAC	**Represent**	0.38965	1.00000	0.18810	0.24175	0.18339	0.34835	0.47396

	**Analog 1**	ATTATAAT	**Analog 1**	0.47282	0.18810	1.00000	0.13464	0.59693	0.47868	0.13280
	**Analog 2**	CCCCTAAA	**Analog 2**	0.29061	0.24175	0.13464	1.00000	0.05912	0.12149	0.05222
	**Analog 3**	ATTATTAA	**Analog 3**	0.36191	0.18339	0.59693	0.05912	1.00000	0.22804	0.25749
	**Analog 4**	GATATAAC	**Analog 4**	0.41985	0.34835	0.47868	0.12149	0.22804	1.00000	0.17755
	**Analog 5**	TAAATCAA	**Analog 5**	0.22558	0.47396	0.13280	0.05222	0.25749	0.17755	1.00000
**64**	**Origin**	TGGATTGT	**Origin**	1.00000	0.14195	0.13146	0.27436	0.36696	0.39523	0.39197
	**Represent**	TGAAGCGC	**Represent**	0.14195	1.00000	0.03773	0.19598	0.53723	0.44374	0.42914
	**Analog 1**	TCGAGTCT	**Analog 1**	0.13146	0.03773	1.00000	-0.08294	-0.06650	0.32078	-0.08294
	**Analog 2**	TGATTTTT	**Analog 2**	0.27436	0.19598	-0.08294	1.00000	0.21242	0.06848	0.41767
	**Analog 3**	TGGTTCGC	**Analog 3**	0.36696	0.53723	-0.06650	0.21242	1.00000	0.27482	0.36230
	**Analog 4**	TGGAGCCT	**Analog 4**	0.39523	0.44374	0.32078	0.06848	0.27482	1.00000	0.02759
	**Analog 5**	TGACTTGC	**Analog 5**	0.39197	0.42914	-0.08294	0.41767	0.36230	0.02759	1.00000
**65**	**Origin**	AGTCTGTC	**Origin**	1.00000	0.34017	0.43759	0.40231	0.16698	0.27909	0.35022
	**Represent**	AGCCTGCC	**Represent**	0.34017	1.00000	0.21639	0.32388	0.55295	0.30237	0.89844
	**Analog 1**	ATTCTGGT	**Analog 1**	0.43759	0.21639	1.00000	0.64319	0.32615	-0.03703	0.22412
	**Analog 2**	AGACTGGT	**Analog 2**	0.40231	0.32388	0.64319	1.00000	0.12302	0.07741	0.33381
	**Analog 3**	ATTATGCC	**Analog 3**	0.16698	0.55295	0.32615	0.12302	1.00000	0.36504	0.40880
	**Analog 4**	AGTATTCC	**Analog 4**	0.27909	0.30237	-0.03703	0.07741	0.36504	1.00000	0.13976
	**Analog 5**	AGCCTGCA	**Analog 5**	0.35022	0.89844	0.22412	0.33381	0.40880	0.13976	1.00000
**66**	**Origin**	TGTGGAGA	**Origin**	1.00000	0.04417	0.60072	0.29591	0.35592	0.27823	0.30048
	**Represent**	CGGTGCGC	**Represent**	0.04417	1.00000	0.18572	0.29306	0.42212	0.35092	-0.01449
	**Analog 1**	CATGGAGC	**Analog 1**	0.60072	0.18572	1.00000	0.10537	0.41397	0.34351	0.21802
	**Analog 2**	CGTTGACA	**Analog 2**	0.29591	0.29306	0.10537	1.00000	-0.02393	-0.01635	-0.07315
	**Analog 3**	TATGGCGG	**Analog 3**	0.35592	0.42212	0.41397	-0.02393	1.00000	0.00702	0.20873
	**Analog 4**	TGGGAAGC	**Analog 4**	0.27823	0.35092	0.34351	-0.01635	0.00702	1.00000	-0.00335
	**Analog 5**	TTCGGTGA	**Analog 5**	0.30048	-0.01449	0.21802	-0.07315	0.20873	-0.00335	1.00000
**67**	**Origin**	GCACCGAG	**Origin**	1.00000	0.21113	0.41945	0.25037	0.23707	0.60894	0.31221
	**Represent**	CGCCCAAG	**Represent**	0.21113	1.00000	0.26944	0.11871	0.24216	0.40775	0.18770
	**Analog 1**	CTACCCAG	**Analog 1**	0.41945	0.26944	1.00000	0.51127	0.06996	0.34560	0.06319
	**Analog 2**	GAACCCCG	**Analog 2**	0.25037	0.11871	0.51127	1.00000	-0.07180	0.25902	-0.06933
	**Analog 3**	CCCCTGAG	**Analog 3**	0.23707	0.24216	0.06996	-0.07180	1.00000	0.09563	0.06950
	**Analog 4**	CGACCGAA	**Analog 4**	0.60894	0.40775	0.34560	0.25902	0.09563	1.00000	-0.03826
	**Analog 5**	GCGTCAAG	**Analog 5**	0.31221	0.18770	0.06319	-0.06933	0.06950	-0.03826	1.00000
**68**	**Origin**	AGACAGGT	**Origin**	1.00000	0.27823	0.34134	0.23157	0.32751	0.23126	0.43277
	**Represent**	TGCCACGT	**Represent**	0.27823	1.00000	0.12749	0.26533	0.42388	0.47309	0.23569
	**Analog 1**	ACACAAGA	**Analog 1**	0.34134	0.12749	1.00000	0.10166	0.06736	-0.02342	0.41394
	**Analog 2**	AGGCACTT	**Analog 2**	0.23157	0.26533	0.10166	1.00000	0.06148	0.43476	0.16014
	**Analog 3**	AGCCTGGA	**Analog 3**	0.32751	0.42388	0.06736	0.06148	1.00000	0.06542	-0.01392
	**Analog 4**	AGGAACGT	**Analog 4**	0.23126	0.47309	-0.02342	0.43476	0.06542	1.00000	-0.02929
	**Analog 5**	TGACAACT	**Analog 5**	0.43277	0.23569	0.41394	0.16014	-0.01392	-0.02929	1.00000

**69**	**Origin**	AGGCGCAT	**Origin**	1.00000	0.62741	0.46203	0.12316	0.44244	0.30293	0.30353
	**Represent**	ACGCGCTT	**Represent**	0.62741	1.00000	0.21242	0.14872	0.20182	0.17637	0.57324
	**Analog 1**	TGGCAGAT	**Analog 1**	0.46203	0.21242	1.00000	0.16801	0.51612	0.07875	0.34682
	**Analog 2**	AGTCACTT	**Analog 2**	0.12316	0.14872	0.16801	1.00000	0.00059	0.00908	-0.06053
	**Analog 3**	GGGCCAAT	**Analog 3**	0.44244	0.20182	0.51612	0.00059	1.00000	0.07732	0.41107
	**Analog 4**	CGCCGTAT	**Analog 4**	0.30293	0.17637	0.07875	0.00908	0.07732	1.00000	0.00373
	**Analog 5**	ACGCTAAT	**Analog 5**	0.30353	0.57324	0.34682	-0.06053	0.41107	0.00373	1.00000
**70**	**Origin**	CTTTCCCG	**Origin**	1.00000	0.34449	0.17195	0.38664	0.50849	0.51498	0.07044
	**Represent**	CTTCCCGG	**Represent**	0.34449	1.00000	0.30915	0.64864	0.15356	0.54871	0.43933
	**Analog 1**	CTTCACAG	**Analog 1**	0.17195	0.30915	1.00000	-0.01526	-0.06098	0.23103	-0.02047
	**Analog 2**	TATTCCGG	**Analog 2**	0.38664	0.64864	-0.01526	1.00000	0.30277	0.21010	0.66784
	**Analog 3**	CCATCCCA	**Analog 3**	0.50849	0.15356	-0.06098	0.30277	1.00000	0.38678	-0.01837
	**Analog 4**	ATTCCCCC	**Analog 4**	0.51498	0.54871	0.23103	0.21010	0.38678	1.00000	-0.02040
	**Analog 5**	CATTTCGG	**Analog 5**	0.07044	0.43933	-0.02047	0.66784	-0.01837	-0.02040	1.00000
**71**	**Origin**	TTTTTCGC	**Origin**	1.00000	0.48364	0.45026	0.44121	0.55074	0.54434	0.32783
	**Represent**	TTCGCCGC	**Represent**	0.48364	1.00000	0.23973	0.15195	0.23535	0.74450	0.16918
	**Analog 1**	GTTTCAGC	**Analog 1**	0.45026	0.23973	1.00000	0.47815	0.09821	0.43638	-0.03198
	**Analog 2**	CGTTTAGC	**Analog 2**	0.44121	0.15195	0.47815	1.00000	0.04475	0.20172	0.02574
	**Analog 3**	ATTCTCGG	**Analog 3**	0.55074	0.23535	0.09821	0.04475	1.00000	0.33975	0.14388
	**Analog 4**	GTTGCCGC	**Analog 4**	0.54434	0.74450	0.43638	0.20172	0.33975	1.00000	-0.02467
	**Analog 5**	TTCTTCTT	**Analog 5**	0.32783	0.16918	-0.03198	0.02574	0.14388	-0.02467	1.00000
**72**	**Origin**	GCAGGCGA	**Origin**	1.00000	0.85840	0.59507	0.55256	0.54281	0.45039	0.27129
	**Represent**	CCAGGCGA	**Represent**	0.85840	1.00000	0.73288	0.38219	0.55687	0.46647	0.42494
	**Analog 1**	CCAGGCCT	**Analog 1**	0.59507	0.73288	1.00000	0.64016	0.34511	0.24711	0.30025
	**Analog 2**	GCATGCCT	**Analog 2**	0.55256	0.38219	0.64016	1.00000	0.35094	0.24704	0.14814
	**Analog 3**	TCACGCGC	**Analog 3**	0.54281	0.55687	0.34511	0.35094	1.00000	0.73343	0.18269
	**Analog 4**	GAACGCGT	**Analog 4**	0.45039	0.46647	0.24711	0.24704	0.73343	1.00000	0.03427
	**Analog 5**	CCAAGAGA	**Analog 5**	0.27129	0.42494	0.30025	0.14814	0.18269	0.03427	1.00000
**73**	**Origin**	CTTGCACG	**Origin**	1.00000	0.69837	0.38908	0.39770	0.48111	0.50435	0.50469
	**Represent**	TATGCACC	**Represent**	0.69837	1.00000	0.25154	0.46735	0.68809	0.55265	0.28150
	**Analog 1**	CATGATCG	**Analog 1**	0.38908	0.25154	1.00000	0.10896	0.15212	0.10201	0.19650
	**Analog 2**	CAAGCATG	**Analog 2**	0.39770	0.46735	0.10896	1.00000	0.51659	0.59244	0.10064
	**Analog 3**	CACGCACC	**Analog 3**	0.48111	0.68809	0.15212	0.51659	1.00000	0.34183	0.22459
	**Analog 4**	GGTGCATG	**Analog 4**	0.50435	0.55265	0.10201	0.59244	0.34183	1.00000	0.08432
	**Analog 5**	TTGACACG	**Analog 5**	0.50469	0.28150	0.19650	0.10064	0.22459	0.08432	1.00000
**74**	**Origin**	ATATAAAT	**Origin**	1.00000	0.01985	0.41780	0.11769	0.49835	0.11416	0.30322
	**Represent**	ATTTTGCC	**Represent**	0.01985	1.00000	0.25768	0.18531	-0.13064	0.40351	0.37543
	**Analog 1**	CTATAACC	**Analog 1**	0.41780	0.25768	1.00000	0.01944	0.13752	0.10979	0.08819
	**Analog 2**	ATTTGAGT	**Analog 2**	0.11769	0.18531	0.01944	1.00000	-0.07177	-0.04614	0.02073
	**Analog 3**	TAAAAAAT	**Analog 3**	0.49835	-0.13064	0.13752	-0.07177	1.00000	-0.02933	-0.02451

	**Analog 4**	ATAGAGCT	**Analog 4**	0.11416	0.40351	0.10979	-0.04614	-0.02933	1.00000	0.11239
	**Analog 5**	ATATTGAG	**Analog 5**	0.30322	0.37543	0.08819	0.02073	-0.02451	0.11239	1.00000
**75**	**Origin**	AGCGCGAG	**Origin**	1.00000	0.57329	0.16406	0.69067	0.33294	0.32980	0.32765
	**Represent**	CGCGGGAG	**Represent**	0.57329	1.00000	0.10502	0.46448	0.26781	0.61549	0.09433
	**Analog 1**	AGGGTCAG	**Analog 1**	0.16406	0.10502	1.00000	0.03524	0.46313	-0.02712	0.60001
	**Analog 2**	TGCGCTAA	**Analog 2**	0.69067	0.46448	0.03524	1.00000	0.29051	0.29264	0.37339
	**Analog 3**	CGGGCAAG	**Analog 3**	0.33294	0.26781	0.46313	0.29051	1.00000	-0.02741	0.66026
	**Analog 4**	ACCGGGAA	**Analog 4**	0.32980	0.61549	-0.02712	0.29264	-0.02741	1.00000	-0.02560
	**Analog 5**	TGGGCCAG	**Analog 5**	0.32765	0.09433	0.60001	0.37339	0.66026	-0.02560	1.00000
**76**	**Origin**	TAAGCAGA	**Origin**	1.00000	0.54174	0.12808	0.67463	0.58615	0.54446	0.27820
	**Represent**	GGTGCAGG	**Represent**	0.54174	1.00000	-0.06117	0.78199	0.57256	0.23968	0.25589
	**Analog 1**	TAATCTCA	**Analog 1**	0.12808	-0.06117	1.00000	-0.05766	-0.00970	0.47926	-0.08710
	**Analog 2**	AGTGCAGA	**Analog 2**	0.67463	0.78199	-0.05766	1.00000	0.45756	0.26366	0.11819
	**Analog 3**	GGAGCAAA	**Analog 3**	0.58615	0.57256	-0.00970	0.45756	1.00000	0.40442	0.11410
	**Analog 4**	TAAGCTCC	**Analog 4**	0.54446	0.23968	0.47926	0.26366	0.40442	1.00000	0.07393
	**Analog 5**	TCAGGAGG	**Analog 5**	0.27820	0.25589	-0.08710	0.11819	0.11410	0.07393	1.00000
**77**	**Origin**	TGACTTAA	**Origin**	1.00000	0.21546	0.25353	0.43748	0.32355	0.13526	0.49513
	**Represent**	CGAATGAC	**Represent**	0.21546	1.00000	0.09389	0.42200	0.29536	0.56516	0.33557
	**Analog 1**	TTACGTAC	**Analog 1**	0.25353	0.09389	1.00000	-0.02630	0.56386	-0.03460	-0.03337
	**Analog 2**	TGAATAAT	**Analog 2**	0.43748	0.42200	-0.02630	1.00000	0.11502	0.26988	0.61952
	**Analog 3**	CGACGTGA	**Analog 3**	0.32355	0.29536	0.56386	0.11502	1.00000	-0.08444	0.14090
	**Analog 4**	TAAATGAA	**Analog 4**	0.13526	0.56516	-0.03460	0.26988	-0.08444	1.00000	0.19193
	**Analog 5**	TGAATTTC	**Analog 5**	0.49513	0.33557	-0.03337	0.61952	0.14090	0.19193	1.00000
**78**	**Origin**	GTTTAGGA	**Origin**	1.00000	0.31847	0.36776	0.42505	0.47324	0.27990	0.51630
	**Represent**	ATTGCGGC	**Represent**	0.31847	1.00000	0.17174	0.83291	0.13303	0.06712	0.50381
	**Analog 1**	GCTCAGGT	**Analog 1**	0.36776	0.17174	1.00000	0.17187	0.13947	-0.02955	0.34593
	**Analog 2**	TTTGCGGA	**Analog 2**	0.42505	0.83291	0.17187	1.00000	0.09606	0.02190	0.23028
	**Analog 3**	ATTTAGAG	**Analog 3**	0.47324	0.13303	0.13947	0.09606	1.00000	0.40262	0.28843
	**Analog 4**	ATTTACTA	**Analog 4**	0.27990	0.06712	-0.02955	0.02190	0.40262	1.00000	0.09297
	**Analog 5**	ATATAGGC	**Analog 5**	0.51630	0.50381	0.34593	0.23028	0.28843	0.09297	1.00000
**79**	**Origin**	AGCTCTGA	**Origin**	1.00000	0.13953	0.14822	0.29830	0.36355	0.56202	0.49325
	**Represent**	GGAGCTTA	**Represent**	0.13953	1.00000	0.11017	0.07456	0.64610	0.28340	0.31089
	**Analog 1**	AGTTGTTA	**Analog 1**	0.14822	0.11017	1.00000	-0.07338	-0.01302	-0.00614	0.19435
	**Analog 2**	ATCACTGC	**Analog 2**	0.29830	0.07456	-0.07338	1.00000	0.22415	0.53316	-0.00615
	**Analog 3**	CGAGCTGA	**Analog 3**	0.36355	0.64610	-0.01302	0.22415	1.00000	0.25877	0.00623
	**Analog 4**	GGCACTGT	**Analog 4**	0.56202	0.28340	-0.00614	0.53316	0.25877	1.00000	0.50506
	**Analog 5**	GGCTATTA	**Analog 5**	0.49325	0.31089	0.19435	-0.00615	0.00623	0.50506	1.00000
**80**	**Origin**	ACCTGCCT	**Origin**	1.00000	0.67095	0.66635	0.47157	0.40361	0.51287	0.59163
	**Represent**	ACCTGCGC	**Represent**	0.67095	1.00000	0.42737	0.42519	0.61129	0.45392	0.93102
	**Analog 1**	GACTGCCA	**Analog 1**	0.66635	0.42737	1.00000	0.48275	0.36841	0.21364	0.43124

	**Analog 2**	TGCTGCTT	**Analog 2**	0.47157	0.42519	0.48275	1.00000	0.35716	0.22086	0.42928
	**Analog 3**	TCTTGCGT	**Analog 3**	0.40361	0.61129	0.36841	0.35716	1.00000	0.12884	0.68547
	**Analog 4**	ACCTGGAG	**Analog 4**	0.51287	0.45392	0.21364	0.22086	0.12884	1.00000	0.36566
	**Analog 5**	TCCTGCGC	**Analog 5**	0.59163	0.93102	0.43124	0.42928	0.68547	0.36566	1.00000
**81**	**Origin**	GGCTAGTT	**Origin**	1.00000	0.18851	0.51168	0.37161	0.26180	0.53907	0.16535
	**Represent**	GGGTCGCT	**Represent**	0.18851	1.00000	0.00351	0.53679	0.33121	0.33798	0.30785
	**Analog 1**	CGCTATAT	**Analog 1**	0.51168	0.00351	1.00000	0.01260	0.12538	0.60021	0.35154
	**Analog 2**	GGTTCGTA	**Analog 2**	0.37161	0.53679	0.01260	1.00000	-0.00896	0.52270	0.03424
	**Analog 3**	TCCTAGCT	**Analog 3**	0.26180	0.33121	0.12538	-0.00896	1.00000	0.10620	-0.04442
	**Analog 4**	CGCTCGTG	**Analog 4**	0.53907	0.33798	0.60021	0.52270	0.10620	1.00000	0.25777
	**Analog 5**	CGGTAATT	**Analog 5**	0.16535	0.30785	0.35154	0.03424	-0.04442	0.25777	1.00000
**82**	**Origin**	GGAACGCG	**Origin**	1.00000	0.82241	0.22520	0.35374	0.27768	0.32132	0.20252
	**Represent**	GTAACGCG	**Represent**	0.82241	1.00000	0.23694	0.51541	0.30600	0.22610	0.39965
	**Analog 1**	GAACCGGG	**Analog 1**	0.22520	0.23694	1.00000	0.24850	-0.07030	-0.02283	-0.01598
	**Analog 2**	GTTACGTG	**Analog 2**	0.35374	0.51541	0.24850	1.00000	0.06815	-0.04861	0.33773
	**Analog 3**	GCGACTCG	**Analog 3**	0.27768	0.30600	-0.07030	0.06815	1.00000	0.16351	0.23845
	**Analog 4**	TGAGCCCG	**Analog 4**	0.32132	0.22610	-0.02283	-0.04861	0.16351	1.00000	-0.01865
	**Analog 5**	GTAACTAG	**Analog 5**	0.20252	0.39965	-0.01598	0.33773	0.23845	-0.01865	1.00000
**83**	**Origin**	GCTCTCTG	**Origin**	1.00000	0.66353	0.22299	0.42205	0.41207	0.60221	0.24414
	**Represent**	GCGCTCTG	**Represent**	0.66353	1.00000	0.60373	0.35638	0.21704	0.36291	0.21678
	**Analog 1**	CCGCTCCG	**Analog 1**	0.22299	0.60373	1.00000	-0.01433	-0.02136	0.21845	0.37694
	**Analog 2**	GCAGTATG	**Analog 2**	0.42205	0.35638	-0.01433	1.00000	0.06006	-0.02974	0.32659
	**Analog 3**	TTTCACTG	**Analog 3**	0.41207	0.21704	-0.02136	0.06006	1.00000	0.35650	0.24849
	**Analog 4**	AATCTCTA	**Analog 4**	0.60221	0.36291	0.21845	-0.02974	0.35650	1.00000	-0.01329
	**Analog 5**	GCACACCG	**Analog 5**	0.24414	0.21678	0.37694	0.32659	0.24849	-0.01329	1.00000
**84**	**Origin**	TCCTTACC	**Origin**	1.00000	0.42138	0.43679	0.24605	0.47357	0.35089	0.30960
	**Represent**	CCCCTCCC	**Represent**	0.42138	1.00000	0.49054	0.16957	0.28176	0.36528	0.33867
	**Analog 1**	AACTTCCC	**Analog 1**	0.43679	0.49054	1.00000	-0.02646	0.12485	0.27755	-0.06672
	**Analog 2**	ACCGTAGC	**Analog 2**	0.24605	0.16957	-0.02646	1.00000	0.39294	-0.02666	0.17806
	**Analog 3**	GCCGCACC	**Analog 3**	0.47357	0.28176	0.12485	0.39294	1.00000	0.13956	0.29897
	**Analog 4**	CCATTTCC	**Analog 4**	0.35089	0.36528	0.27755	-0.02666	0.13956	1.00000	-0.05809
	**Analog 5**	TCCCCAAC	**Analog 5**	0.30960	0.33867	-0.06672	0.17806	0.29897	-0.05809	1.00000
**85**	**Origin**	TGAAGCGA	**Origin**	1.00000	0.48032	0.44986	0.27451	0.56755	0.46613	0.40631
	**Represent**	TGCAGCAA	**Represent**	0.48032	1.00000	-0.00734	-0.00499	0.64044	0.04518	0.58306
	**Analog 1**	CAAACCGA	**Analog 1**	0.44986	-0.00734	1.00000	0.06915	0.23104	0.35138	-0.04266
	**Analog 2**	AGATGTGA	**Analog 2**	0.27451	-0.00499	0.06915	1.00000	0.04832	0.13333	0.16168
	**Analog 3**	CGCAGCGG	**Analog 3**	0.56755	0.64044	0.23104	0.04832	1.00000	0.43691	0.25681
	**Analog 4**	CGAAACGC	**Analog 4**	0.46613	0.04518	0.35138	0.13333	0.43691	1.00000	0.00936
	**Analog 5**	TGTTGCAA	**Analog 5**	0.40631	0.58306	-0.04266	0.16168	0.25681	0.00936	1.00000
**86**	**Origin**	ATTTACGT	**Origin**	1.00000	0.28067	0.23741	0.18343	0.27716	0.52623	0.66495

	**Represent**	ATTTCAGG	**Represent**	0.28067	1.00000	0.59443	0.50891	0.13736	0.17663	0.10327
	**Analog 1**	AGTTCAGT	**Analog 1**	0.23741	0.59443	1.00000	0.59655	0.10490	-0.02247	-0.02035
	**Analog 2**	AGTTAAGG	**Analog 2**	0.18343	0.50891	0.59655	1.00000	-0.00164	0.23499	0.03732
	**Analog 3**	ATTGGTGT	**Analog 3**	0.27716	0.13736	0.10490	-0.00164	1.00000	-0.04814	0.08721
	**Analog 4**	ACATACGG	**Analog 4**	0.52623	0.17663	-0.02247	0.23499	-0.04814	1.00000	0.47032
	**Analog 5**	CTTAACGA	**Analog 5**	0.66495	0.10327	-0.02035	0.03732	0.08721	0.47032	1.00000
**87**	**Origin**	TAGACCGG	**Origin**	1.00000	0.33858	0.13584	0.16764	0.34877	0.64814	0.25734
	**Represent**	TACACCCG	**Represent**	0.33858	1.00000	0.45688	0.27178	0.38139	0.18400	-0.01820
	**Analog 1**	TAAACACG	**Analog 1**	0.13584	0.45688	1.00000	0.31149	0.31269	-0.04205	-0.01119
	**Analog 2**	TAGAAGCG	**Analog 2**	0.16764	0.27178	0.31149	1.00000	-0.04742	-0.10121	0.26516
	**Analog 3**	GACACAGG	**Analog 3**	0.34877	0.38139	0.31269	-0.04742	1.00000	0.29477	-0.02974
	**Analog 4**	GATTCCGG	**Analog 4**	0.64814	0.18400	-0.04205	-0.10121	0.29477	1.00000	-0.08998
	**Analog 5**	TAGATTTG	**Analog 5**	0.25734	-0.01820	-0.01119	0.26516	-0.02974	-0.08998	1.00000
**88**	**Origin**	CGAACATG	**Origin**	1.00000	0.35103	0.38996	0.37475	0.49136	0.25973	0.33246
	**Represent**	CGGAGCTG	**Represent**	0.35103	1.00000	0.51514	0.10332	0.06644	0.13541	0.60904
	**Analog 1**	CGGGGATG	**Analog 1**	0.38996	0.51514	1.00000	0.04828	0.09257	0.13500	0.14552
	**Analog 2**	CTGACATC	**Analog 2**	0.37475	0.10332	0.04828	1.00000	0.45735	-0.03257	-0.03988
	**Analog 3**	GTCACATG	**Analog 3**	0.49136	0.06644	0.09257	0.45735	1.00000	-0.03192	0.08116
	**Analog 4**	GGAAGAAG	**Analog 4**	0.25973	0.13541	0.13500	-0.03257	-0.03192	1.00000	0.52753
	**Analog 5**	GGAAGCTG	**Analog 5**	0.33246	0.60904	0.14552	-0.03988	0.08116	0.52753	1.00000
**89**	**Origin**	TGCTACGA	**Origin**	1.00000	0.01121	0.17953	0.40527	0.54342	0.48548	0.30934
	**Represent**	TCCGGCAC	**Represent**	0.01121	1.00000	0.23431	0.19502	0.54545	-0.06108	0.27175
	**Analog 1**	TCCTATGG	**Analog 1**	0.17953	0.23431	1.00000	0.14492	0.00665	-0.05087	0.13809
	**Analog 2**	TGATGCGG	**Analog 2**	0.40527	0.19502	0.14492	1.00000	0.44923	0.38485	-0.01248
	**Analog 3**	AGCGGCGA	**Analog 3**	0.54342	0.54545	0.00665	0.44923	1.00000	0.30198	0.00645
	**Analog 4**	TGGATCGA	**Analog 4**	0.48548	-0.06108	-0.05087	0.38485	0.30198	1.00000	-0.05788
	**Analog 5**	TTCTACAC	**Analog 5**	0.30934	0.27175	0.13809	-0.01248	0.00645	-0.05788	1.00000
**90**	**Origin**	TTTTCTTA	**Origin**	1.00000	0.18031	0.26472	0.17262	0.21746	0.21223	0.14482
	**Represent**	TATTACTA	**Represent**	0.18031	1.00000	-0.03066	0.29114	0.55757	0.02680	-0.05897
	**Analog 1**	TTTCCATC	**Analog 1**	0.26472	-0.03066	1.00000	0.05266	0.11419	-0.01287	0.10506
	**Analog 2**	TTATTCTA	**Analog 2**	0.17262	0.29114	0.05266	1.00000	0.02931	-0.09332	0.07073
	**Analog 3**	TATTAATA	**Analog 3**	0.21746	0.55757	0.11419	0.02931	1.00000	0.05143	-0.05400
	**Analog 4**	TGTTCGCA	**Analog 4**	0.21223	0.02680	-0.01287	-0.09332	0.05143	1.00000	0.25069
	**Analog 5**	TTGACGTA	**Analog 5**	0.14482	-0.05897	0.10506	0.07073	-0.05400	0.25069	1.00000
**91**	**Origin**	GAGTTTAC	**Origin**	1.00000	0.20396	0.32520	0.27874	0.39920	0.23802	0.20519
	**Represent**	GGGTTGCC	**Represent**	0.20396	1.00000	-0.08021	0.15700	0.05023	0.46355	0.62739
	**Analog 1**	GAAGTTAG	**Analog 1**	0.32520	-0.08021	1.00000	0.05691	0.22857	-0.04001	0.21264
	**Analog 2**	GCGTTAAG	**Analog 2**	0.27874	0.15700	0.05691	1.00000	0.10326	0.14107	0.01145
	**Analog 3**	TATTTTAA	**Analog 3**	0.39920	0.05023	0.22857	0.10326	1.00000	0.04467	0.10821
	**Analog 4**	GGGTGTAT	**Analog 4**	0.23802	0.46355	-0.04001	0.14107	0.04467	1.00000	-0.07761

	**Analog 5**	GAATTGCC	**Analog 5**	0.20519	0.62739	0.21264	0.01145	0.10821	-0.07761	1.00000
**92**	**Origin**	TAGTTCGC	**Origin**	1.00000	0.12236	0.27291	0.35610	0.15135	0.20067	0.23078
	**Represent**	TGGGTCTC	**Represent**	0.12236	1.00000	0.33632	0.63284	0.59285	0.33976	0.33033
	**Analog 1**	TGGTTAGG	**Analog 1**	0.27291	0.33632	1.00000	0.61120	0.49984	0.22994	0.02678
	**Analog 2**	TGGTTCTT	**Analog 2**	0.35610	0.63284	0.61120	1.00000	0.70772	-0.01027	0.36865
	**Analog 3**	TGGTACTC	**Analog 3**	0.15135	0.59285	0.49984	0.70772	1.00000	-0.05493	0.24739
	**Analog 4**	TAGGTTGG	**Analog 4**	0.20067	0.33976	0.22994	-0.01027	-0.05493	1.00000	-0.04480
	**Analog 5**	AACTTCTC	**Analog 5**	0.23078	0.33033	0.02678	0.36865	0.24739	-0.04480	1.00000
**93**	**Origin**	TATCTTAT	**Origin**	1.00000	-0.02767	0.42593	0.57018	0.26413	0.17708	0.22511
	**Represent**	TGTGGTTT	**Represent**	-0.02767	1.00000	-0.06785	-0.07368	0.27383	0.19596	0.58065
	**Analog 1**	TCTCTAAC	**Analog 1**	0.42593	-0.06785	1.00000	0.53040	0.30862	-0.08129	-0.03421
	**Analog 2**	CATCTAAG	**Analog 2**	0.57018	-0.07368	0.53040	1.00000	0.16966	-0.03089	0.23841
	**Analog 3**	TGTCCTAC	**Analog 3**	0.26413	0.27383	0.30862	0.16966	1.00000	-0.09300	0.01320
	**Analog 4**	TAAGTTTT	**Analog 4**	0.17708	0.19596	-0.08129	-0.03089	-0.09300	1.00000	0.04141
	**Analog 5**	CATGGTAT	**Analog 5**	0.22511	0.58065	-0.03421	0.23841	0.01320	0.04141	1.00000
**94**	**Origin**	CCATTCTA	**Origin**	1.00000	-0.05121	0.34363	0.30005	0.44131	0.25015	0.17406
	**Represent**	CTTGGCGT	**Represent**	-0.05121	1.00000	0.19836	0.10913	0.30435	-0.05992	0.18322
	**Analog 1**	CCGTGCTG	**Analog 1**	0.34363	0.19836	1.00000	0.14956	0.17802	0.68565	0.10907
	**Analog 2**	CCTGTATA	**Analog 2**	0.30005	0.10913	0.14956	1.00000	0.17453	0.20172	-0.00819
	**Analog 3**	CCCTTCGT	**Analog 3**	0.44131	0.30435	0.17802	0.17453	1.00000	0.14700	-0.00939
	**Analog 4**	CCGTGTTA	**Analog 4**	0.25015	-0.05992	0.68565	0.20172	0.14700	1.00000	-0.02218
	**Analog 5**	CTTTCCTA	**Analog 5**	0.17406	0.18322	0.10907	-0.00819	-0.00939	-0.02218	1.00000
**95**	**Origin**	GGAGACGT	**Origin**	1.00000	0.28318	0.36416	0.29583	0.31928	0.36949	0.55257
	**Represent**	CGACAAGT	**Represent**	0.28318	1.00000	-0.01708	0.08420	0.42582	0.41660	0.44577
	**Analog 1**	GGCGACCC	**Analog 1**	0.36416	-0.01708	1.00000	0.13532	-0.01135	0.13585	0.11367
	**Analog 2**	GGAACCAT	**Analog 2**	0.29583	0.08420	0.13532	1.00000	0.05481	0.40476	0.13572
	**Analog 3**	CGAGGGGT	**Analog 3**	0.31928	0.42582	-0.01135	0.05481	1.00000	0.13418	0.10265
	**Analog 4**	GGAAAAGA	**Analog 4**	0.36949	0.41660	0.13585	0.40476	0.13418	1.00000	0.29559
	**Analog 5**	TGACACGA	**Analog 5**	0.55257	0.44577	0.11367	0.13572	0.10265	0.29559	1.00000
**96**	**Origin**	TCGAGCGC	**Origin**	1.00000	0.56129	0.35574	0.31750	0.44310	0.37245	0.35529
	**Represent**	GCGAGGGC	**Represent**	0.56129	1.00000	0.35870	0.44348	0.18126	0.37565	0.62665
	**Analog 1**	CCTAGTGC	**Analog 1**	0.35574	0.35870	1.00000	-0.01990	0.22940	0.58833	0.30333
	**Analog 2**	GCGACCAC	**Analog 2**	0.31750	0.44348	-0.01990	1.00000	0.26506	-0.02092	0.02356
	**Analog 3**	TTAACCGC	**Analog 3**	0.44310	0.18126	0.22940	0.26506	1.00000	0.24601	0.19043
	**Analog 4**	ACTAGAGC	**Analog 4**	0.37245	0.37565	0.58833	-0.02092	0.24601	1.00000	0.32386
	**Analog 5**	CGGAGGGC	**Analog 5**	0.35529	0.62665	0.30333	0.02356	0.19043	0.32386	1.00000
**97**	**Origin**	TACTTGCA	**Origin**	1.00000	-0.00984	0.19827	0.16527	0.42419	0.13078	0.23532
	**Represent**	TGGTGGGA	**Represent**	-0.00984	1.00000	-0.13343	-0.05860	-0.09467	0.56491	0.09703
	**Analog 1**	TAACTTCA	**Analog 1**	0.19827	-0.13343	1.00000	0.61703	0.13114	-0.13352	0.40564
	**Analog 2**	TAGCTTCA	**Analog 2**	0.16527	-0.05860	0.61703	1.00000	0.10808	-0.13168	0.34505

	**Analog 3**	GACTACCA	**Analog 3**	0.42419	-0.09467	0.13114	0.10808	1.00000	0.05034	0.11428
	**Analog 4**	TCCTGGGA	**Analog 4**	0.13078	0.56491	-0.13352	-0.13168	0.05034	1.00000	-0.08995
	**Analog 5**	TGTTTTCA	**Analog 5**	0.23532	0.09703	0.40564	0.34505	0.11428	-0.08995	1.00000
**98**	**Origin**	TGACAATA	**Origin**	1.00000	0.74273	0.54004	0.51760	0.27897	0.61393	0.33461
	**Represent**	TGACATTC	**Represent**	0.74273	1.00000	0.36986	0.66369	0.24610	0.53396	0.19089
	**Analog 1**	TGATAAAT	**Analog 1**	0.54004	0.36986	1.00000	-0.00973	0.26669	0.12405	-0.01278
	**Analog 2**	ATACATTA	**Analog 2**	0.51760	0.66369	-0.00973	1.00000	-0.01957	0.56307	0.26320
	**Analog 3**	TGAGCCTA	**Analog 3**	0.27897	0.24610	0.26669	-0.01957	1.00000	-0.04830	0.06176
	**Analog 4**	GTACAATC	**Analog 4**	0.61393	0.53396	0.12405	0.56307	-0.04830	1.00000	0.30427
	**Analog 5**	AAACCATA	**Analog 5**	0.33461	0.19089	-0.01278	0.26320	0.06176	0.30427	1.00000
**99**	**Origin**	CATCGGAA	**Origin**	1.00000	0.25162	0.31463	0.23255	0.13534	0.27557	0.22175
	**Represent**	CAAGCGAA	**Represent**	0.25162	1.00000	0.21030	0.40049	0.32451	0.60606	0.32817
	**Analog 1**	AATTTGAA	**Analog 1**	0.31463	0.21030	1.00000	0.04613	-0.01702	0.14582	0.04667
	**Analog 2**	CAAGGTAA	**Analog 2**	0.23255	0.40049	0.04613	1.00000	0.01476	0.18175	0.36638
	**Analog 3**	CAGCCGCA	**Analog 3**	0.13534	0.32451	-0.01702	0.01476	1.00000	0.53114	0.31518
	**Analog 4**	CAACCGAT	**Analog 4**	0.27557	0.60606	0.14582	0.18175	0.53114	1.00000	0.65375
	**Analog 5**	CAACCTAA	**Analog 5**	0.22175	0.32817	0.04667	0.36638	0.31518	0.65375	1.00000
**100**	**Origin**	GGGATACT	**Origin**	1.00000	0.13129	0.52962	0.47088	0.43194	0.41476	0.18278
	**Represent**	GCGCAACG	**Represent**	0.13129	1.00000	0.36222	0.24249	0.23861	0.40695	0.39011
	**Analog 1**	CGGAAACG	**Analog 1**	0.52962	0.36222	1.00000	0.48209	0.21378	0.29316	0.08727
	**Analog 2**	AAGAAACT	**Analog 2**	0.47088	0.24249	0.48209	1.00000	0.00253	0.09998	0.20378
	**Analog 3**	GGGCTTCC	**Analog 3**	0.43194	0.23861	0.21378	0.00253	1.00000	0.59786	-0.01202
	**Analog 4**	GGGCAAAT	**Analog 4**	0.41476	0.40695	0.29316	0.09998	0.59786	1.00000	-0.05587
	**Analog 5**	GCGAGCCT	**Analog 5**	0.18278	0.39011	0.08727	0.20378	-0.01202	-0.05587	1.00000

Supplementary 7. The random sets of five Analogs with mutation number variation.

Mismatch-1		Sequence	Pearson's correlation coefficient	Origin	Represent	Analog 1	Analog 2	Analog 3	Analog 4	Analog 5
1	**Origin**	TGCATACC	**Origin**	1.00000	0.61568	0.85860	0.59815	0.83867	0.66183	0.72867
	**Represent**	TGCGCACC	**Represent**	0.61568	1.00000	0.49636	0.36339	0.68775	0.41399	0.79049
	**Analog 1**	TGCATACA	**Analog 1**	0.85860	0.49636	1.00000	0.43361	0.70803	0.48010	0.59708
	**Analog 2**	TGGATACC	**Analog 2**	0.59815	0.36339	0.43361	1.00000	0.46778	0.70263	0.45045
	**Analog 3**	TGCACACC	**Analog 3**	0.83867	0.68775	0.70803	0.46778	1.00000	0.51423	0.62207
	**Analog 4**	TGTATACC	**Analog 4**	0.66183	0.41399	0.48010	0.70263	0.51423	1.00000	0.51006
	**Analog 5**	TGCGTACC	**Analog 5**	0.72867	0.79049	0.59708	0.45045	0.62207	0.51006	1.00000
2	**Origin**	CTAGCGTT	**Origin**	1.00000	1.00000	0.81684	0.55077	0.81684	0.68340	0.75781
	**Represent**	CTAGCGTT	**Represent**	1.00000	1.00000	0.81684	0.55077	0.81684	0.68340	0.75781
	**Analog 1**	CTAGCGAT	**Analog 1**	0.81684	0.81684	1.00000	0.31070	1.00000	0.59424	0.77318
	**Analog 2**	CTAGAGTT	**Analog 2**	0.55077	0.55077	0.31070	1.00000	0.31070	0.41157	0.28527
	**Analog 3**	CTAGCGAT	**Analog 3**	0.81684	0.81684	1.00000	0.31070	1.00000	0.59424	0.77318
	**Analog 4**	CTAGCTTT	**Analog 4**	0.68340	0.68340	0.59424	0.41157	0.59424	1.00000	0.54207
	**Analog 5**	CTAGCGGT	**Analog 5**	0.75781	0.75781	0.77318	0.28527	0.77318	0.54207	1.00000
3	**Origin**	TAGCTTGC	**Origin**	1.00000	0.75441	0.68542	0.70055	0.93926	0.75441	0.83164
	**Represent**	TAGCTGGC	**Represent**	0.75441	1.00000	0.55060	0.44765	0.70558	1.00000	0.57747
	**Analog 1**	TAGCTTAC	**Analog 1**	0.68542	0.55060	1.00000	0.27691	0.63622	0.55060	0.75227
	**Analog 2**	TATCTTGC	**Analog 2**	0.70055	0.44765	0.27691	1.00000	0.64817	0.44765	0.47148
	**Analog 3**	CAGCTTGC	**Analog 3**	0.93926	0.70558	0.63622	0.64817	1.00000	0.70558	0.77699
	**Analog 4**	TAGCTGGC	**Analog 4**	0.75441	1.00000	0.55060	0.44765	0.70558	1.00000	0.57747
	**Analog 5**	TAGCTTGA	**Analog 5**	0.83164	0.57747	0.75227	0.47148	0.77699	0.57747	1.00000
4	**Origin**	CGCGCTCG	**Origin**	1.00000	1.00000	0.73638	0.89480	0.81055	0.72041	0.83286
	**Represent**	CGCGCTCG	**Represent**	1.00000	1.00000	0.73638	0.89480	0.81055	0.72041	0.83286
	**Analog 1**	CTCGCTCG	**Analog 1**	0.73638	0.73638	1.00000	0.60148	0.50038	0.37980	0.51457
	**Analog 2**	CGCGCTCA	**Analog 2**	0.89480	0.89480	0.60148	1.00000	0.84740	0.58701	0.86995
	**Analog 3**	CGCGCTTG	**Analog 3**	0.81055	0.81055	0.50038	0.84740	1.00000	0.48714	0.88162
	**Analog 4**	CGCACTCG	**Analog 4**	0.72041	0.72041	0.37980	0.58701	0.48714	1.00000	0.50097
	**Analog 5**	CGCGCTAG	**Analog 5**	0.83286	0.83286	0.51457	0.86995	0.88162	0.50097	1.00000
5	**Origin**	GTAAATCC	**Origin**	1.00000	0.43856	0.72249	0.68396	0.72249	0.81405	0.66984
	**Represent**	GTAAACCG	**Represent**	0.43856	1.00000	0.70060	0.20934	0.70060	0.25646	0.77814
	**Analog 1**	GTAAATCG	**Analog 1**	0.72249	0.70060	1.00000	0.44471	1.00000	0.53467	0.43639
	**Analog 2**	GGAAATCC	**Analog 2**	0.68396	0.20934	0.44471	1.00000	0.44471	0.50324	0.40979
	**Analog 3**	GTAAATCG	**Analog 3**	0.72249	0.70060	1.00000	0.44471	1.00000	0.53467	0.43639
	**Analog 4**	GTATATCC	**Analog 4**	0.81405	0.25646	0.53467	0.50324	0.53467	1.00000	0.49034

	**Analog 5**	GTAAACCC	**Analog 5**	0.66984	0.77814	0.43639	0.40979	0.43639	0.49034	1.00000
**6**	**Origin**	ATTTGCAC	**Origin**	1.00000	0.36214	0.81003	0.72219	0.60885	0.78826	0.78826
	**Represent**	AGGTGCGC	**Represent**	0.36214	1.00000	0.44906	0.37344	0.74560	0.47179	0.47179
	**Analog 1**	AGTTGCAC	**Analog 1**	0.81003	0.44906	1.00000	0.54353	0.45852	0.66480	0.66480
	**Analog 2**	ATTTGCTC	**Analog 2**	0.72219	0.37344	0.54353	1.00000	0.62760	0.52391	0.52391
	**Analog 3**	ATTTGCGC	**Analog 3**	0.60885	0.74560	0.45852	0.62760	1.00000	0.44201	0.44201
	**Analog 4**	ATGTGCAC	**Analog 4**	0.78826	0.47179	0.66480	0.52391	0.44201	1.00000	1.00000
	**Analog 5**	ATGTGCAC	**Analog 5**	0.78826	0.47179	0.66480	0.52391	0.44201	1.00000	1.00000
**7**	**Origin**	GACCTGCA	**Origin**	1.00000	1.00000	0.70918	0.94794	0.77930	0.74905	0.71675
	**Represent**	GACCTGCA	**Represent**	1.00000	1.00000	0.70918	0.94794	0.77930	0.74905	0.71675
	**Analog 1**	GGCCTGCA	**Analog 1**	0.70918	0.70918	1.00000	0.74205	0.70882	0.55537	0.42444
	**Analog 2**	TACCTGCA	**Analog 2**	0.94794	0.94794	0.74205	1.00000	0.81338	0.68435	0.64179
	**Analog 3**	GTCCTGCA	**Analog 3**	0.77930	0.77930	0.70882	0.81338	1.00000	0.62456	0.46460
	**Analog 4**	GAACTGCA	**Analog 4**	0.74905	0.74905	0.55537	0.68435	0.62456	1.00000	0.40445
	**Analog 5**	GACCTGTA	**Analog 5**	0.71675	0.71675	0.42444	0.64179	0.46460	0.40445	1.00000
**8**	**Origin**	ACGTGTAC	**Origin**	1.00000	1.00000	0.77505	0.86927	0.91342	0.74305	0.66527
	**Represent**	ACGTGTAC	**Represent**	1.00000	1.00000	0.77505	0.86927	0.91342	0.74305	0.66527
	**Analog 1**	ACGTTTAC	**Analog 1**	0.77505	0.77505	1.00000	0.64338	0.67414	0.79506	0.37289
	**Analog 2**	CCGTGTAC	**Analog 2**	0.86927	0.86927	0.64338	1.00000	0.78239	0.61691	0.64361
	**Analog 3**	ACGTGTAA	**Analog 3**	0.91342	0.91342	0.67414	0.78239	1.00000	0.64641	0.55307
	**Analog 4**	ACGTCTAC	**Analog 4**	0.74305	0.74305	0.79506	0.61691	0.64641	1.00000	0.35792
	**Analog 5**	AAGTGTAC	**Analog 5**	0.66527	0.66527	0.37289	0.64361	0.55307	0.35792	1.00000
**9**	**Origin**	AGGCTATA	**Origin**	1.00000	0.05589	0.41089	0.83377	0.75780	0.93475	0.61884
	**Represent**	AGCACCTC	**Represent**	0.05589	1.00000	0.40158	0.17678	0.09553	0.14986	0.14128
	**Analog 1**	AGCCTATA	**Analog 1**	0.41089	0.40158	1.00000	0.28924	0.22972	0.36990	0.29190
	**Analog 2**	AGGCTCTA	**Analog 2**	0.83377	0.17678	0.28924	1.00000	0.62174	0.77953	0.46208
	**Analog 3**	AGGCCATA	**Analog 3**	0.75780	0.09553	0.22972	0.62174	1.00000	0.70509	0.49356
	**Analog 4**	AGGCTATC	**Analog 4**	0.93475	0.14986	0.36990	0.77953	0.70509	1.00000	0.56490
	**Analog 5**	AGGATATA	**Analog 5**	0.61884	0.14128	0.29190	0.46208	0.49356	0.56490	1.00000
**10**	**Origin**	CTGTAGTA	**Origin**	1.00000	1.00000	0.66726	0.91175	0.90171	0.75126	0.69484
	**Represent**	CTGTAGTA	**Represent**	1.00000	1.00000	0.66726	0.91175	0.90171	0.75126	0.69484
	**Analog 1**	CTTTAGTA	**Analog 1**	0.66726	0.66726	1.00000	0.59861	0.54389	0.39802	0.36857
	**Analog 2**	CTGTAGTG	**Analog 2**	0.91175	0.91175	0.59861	1.00000	0.81912	0.69372	0.63956
	**Analog 3**	ATGTAGTA	**Analog 3**	0.90171	0.90171	0.54389	0.81912	1.00000	0.64551	0.59717
	**Analog 4**	CTGTAGAA	**Analog 4**	0.75126	0.75126	0.39802	0.69372	0.64551	1.00000	0.67951
	**Analog 5**	CTGTAGGA	**Analog 5**	0.69484	0.69484	0.36857	0.63956	0.59717	0.67951	1.00000
**11**	**Origin**	GCATTTAC	**Origin**	1.00000	0.77731	0.76781	0.77731	0.54181	0.84140	0.66676
	**Represent**	GCACTTAC	**Represent**	0.77731	1.00000	0.54289	1.00000	0.32030	0.70121	0.51432

	**Analog 1**	TCATTTAC	**Analog 1**	0.76781	0.54289	1.00000	0.54289	0.61975	0.57391	0.43249
	**Analog 2**	GCACTTAC	**Analog 2**	0.77731	1.00000	0.54289	1.00000	0.32030	0.70121	0.51432
	**Analog 3**	GAATTTAC	**Analog 3**	0.54181	0.32030	0.61975	0.32030	1.00000	0.32476	0.36373
	**Analog 4**	GCATATAC	**Analog 4**	0.84140	0.70121	0.57391	0.70121	0.32476	1.00000	0.51724
	**Analog 5**	GCGTTTAC	**Analog 5**	0.66676	0.51432	0.43249	0.51432	0.36373	0.51724	1.00000
**12**	**Origin**	AATAGCGA	**Origin**	1.00000	1.00000	0.81968	0.81945	0.87724	0.81945	0.44513
	**Represent**	AATAGCGA	**Represent**	1.00000	1.00000	0.81968	0.81945	0.87724	0.81945	0.44513
	**Analog 1**	AGTAGCGA	**Analog 1**	0.81968	0.81968	1.00000	0.77390	0.70832	0.77390	0.27931
	**Analog 2**	ACTAGCGA	**Analog 2**	0.81945	0.81945	0.77390	1.00000	0.70802	1.00000	0.27844
	**Analog 3**	AATAGCGG	**Analog 3**	0.87724	0.87724	0.70832	0.70802	1.00000	0.70802	0.33302
	**Analog 4**	ACTAGCGA	**Analog 4**	0.81945	0.81945	0.77390	1.00000	0.70802	1.00000	0.27844
	**Analog 5**	AATAGGGA	**Analog 5**	0.44513	0.44513	0.27931	0.27844	0.33302	0.27844	1.00000
**13**	**Origin**	ACTTGCAG	**Origin**	1.00000	1.00000	0.90358	0.89901	0.67776	0.87022	0.74703
	**Represent**	ACTTGCAG	**Represent**	1.00000	1.00000	0.90358	0.89901	0.67776	0.87022	0.74703
	**Analog 1**	ACTTGCAC	**Analog 1**	0.90358	0.90358	1.00000	0.80301	0.66770	0.77760	0.65887
	**Analog 2**	TCTTGCAG	**Analog 2**	0.89901	0.89901	0.80301	1.00000	0.58438	0.87793	0.65034
	**Analog 3**	ACTTGCCG	**Analog 3**	0.67776	0.67776	0.66770	0.58438	1.00000	0.56648	0.46313
	**Analog 4**	CCTTGCAG	**Analog 4**	0.87022	0.87022	0.77760	0.87793	0.56648	1.00000	0.63020
	**Analog 5**	ACTGGCAG	**Analog 5**	0.74703	0.74703	0.65887	0.65034	0.46313	0.63020	1.00000
**14**	**Origin**	GTCTTTCC	**Origin**	1.00000	0.25838	0.77126	0.78120	0.78242	0.69087	0.78120
	**Represent**	GACTTACG	**Represent**	0.25838	1.00000	0.51350	0.45900	0.14996	0.55932	0.45900
	**Analog 1**	GTCTTTCG	**Analog 1**	0.77126	0.51350	1.00000	0.55931	0.57165	0.47596	0.55931
	**Analog 2**	GTCTTACC	**Analog 2**	0.78120	0.45900	0.55931	1.00000	0.64406	0.46719	1.00000
	**Analog 3**	GTCTCTCC	**Analog 3**	0.78242	0.14996	0.57165	0.64406	1.00000	0.48777	0.64406
	**Analog 4**	GACTTTCC	**Analog 4**	0.69087	0.55932	0.47596	0.46719	0.48777	1.00000	0.46719
	**Analog 5**	GTCTTACC	**Analog 5**	0.78120	0.45900	0.55931	1.00000	0.64406	0.46719	1.00000
**15**	**Origin**	AACCAATT	**Origin**	1.00000	0.28973	0.77526	0.77526	0.54027	0.82900	0.94361
	**Represent**	AACGAGTA	**Represent**	0.28973	1.00000	0.54349	0.54349	0.73756	0.28891	0.32331
	**Analog 1**	AACCAGTT	**Analog 1**	0.77526	0.54349	1.00000	1.00000	0.35341	0.77515	0.71645
	**Analog 2**	AACCAGTT	**Analog 2**	0.77526	0.54349	1.00000	1.00000	0.35341	0.77515	0.71645
	**Analog 3**	AACGAATT	**Analog 3**	0.54027	0.73756	0.35341	0.35341	1.00000	0.37564	0.47489
	**Analog 4**	AACCATTT	**Analog 4**	0.82900	0.28891	0.77515	0.77515	0.37564	1.00000	0.76738
	**Analog 5**	AACCAATA	**Analog 5**	0.94361	0.32331	0.71645	0.71645	0.47489	0.76738	1.00000
**16**	**Origin**	CAGGAGGG	**Origin**	1.00000	0.42189	0.76422	0.76908	0.67926	0.69679	0.69679
	**Represent**	CCGGACGG	**Represent**	0.42189	1.00000	0.17815	0.42305	0.72417	0.73617	0.73617
	**Analog 1**	CAGTAGGG	**Analog 1**	0.76422	0.17815	1.00000	0.51428	0.43210	0.46460	0.46460
	**Analog 2**	CTGGAGGG	**Analog 2**	0.76908	0.42305	0.51428	1.00000	0.46092	0.69733	0.69733
	**Analog 3**	CAGGACGG	**Analog 3**	0.67926	0.72417	0.43210	0.46092	1.00000	0.41794	0.41794

	**Analog 4**	CCGGAGGG	**Analog 4**	0.69679	0.73617	0.46460	0.69733	0.41794	1.00000	1.00000
	**Analog 5**	CCGGAGGG	**Analog 5**	0.69679	0.73617	0.46460	0.69733	0.41794	1.00000	1.00000
**17**	**Origin**	AAGAATCA	**Origin**	1.00000	1.00000	0.63288	0.71556	0.72813	0.93650	0.67757
	**Represent**	AAGAATCA	**Represent**	1.00000	1.00000	0.63288	0.71556	0.72813	0.93650	0.67757
	**Analog 1**	ACGAATCA	**Analog 1**	0.63288	0.63288	1.00000	0.36005	0.62955	0.65862	0.37923
	**Analog 2**	AAGAATTA	**Analog 2**	0.71556	0.71556	0.36005	1.00000	0.41469	0.63158	0.52793
	**Analog 3**	ATGAATCA	**Analog 3**	0.72813	0.72813	0.62955	0.41469	1.00000	0.75563	0.43654
	**Analog 4**	TAGAATCA	**Analog 4**	0.93650	0.93650	0.65862	0.63158	0.75563	1.00000	0.61395
	**Analog 5**	AAGAACCA	**Analog 5**	0.67757	0.67757	0.37923	0.52793	0.43654	0.61395	1.00000
**18**	**Origin**	CTCCCCAA	**Origin**	1.00000	0.66362	0.92961	0.66362	0.73035	0.68193	0.57483
	**Represent**	CTCCCGAA	**Represent**	0.66362	1.00000	0.59052	1.00000	0.42409	0.39607	0.39676
	**Analog 1**	TTCCCCAA	**Analog 1**	0.92961	0.59052	1.00000	0.59052	0.74385	0.69498	0.50477
	**Analog 2**	CTCCCGAA	**Analog 2**	0.66362	1.00000	0.59052	1.00000	0.42409	0.39607	0.39676
	**Analog 3**	CCCCCCAA	**Analog 3**	0.73035	0.42409	0.74385	0.42409	1.00000	0.62854	0.35188
	**Analog 4**	CGCCCCAA	**Analog 4**	0.68193	0.39607	0.69498	0.39607	0.62854	1.00000	0.32866
	**Analog 5**	CTCCGCAA	**Analog 5**	0.57483	0.39676	0.50477	0.39676	0.35188	0.32866	1.00000
**19**	**Origin**	TCGCGCGC	**Origin**	1.00000	1.00000	0.94735	0.76980	0.75706	0.75656	0.77116
	**Represent**	TCGCGCGC	**Represent**	1.00000	1.00000	0.94735	0.76980	0.75706	0.75656	0.77116
	**Analog 1**	ACGCGCGC	**Analog 1**	0.94735	0.94735	1.00000	0.71263	0.70068	0.70046	0.71457
	**Analog 2**	TCGCGAGC	**Analog 2**	0.76980	0.76980	0.71263	1.00000	0.46219	0.63895	0.65062
	**Analog 3**	TCACGCGC	**Analog 3**	0.75706	0.75706	0.70068	0.46219	1.00000	0.45759	0.46787
	**Analog 4**	TCGCACGC	**Analog 4**	0.75656	0.75656	0.70046	0.63895	0.45759	1.00000	0.46962
	**Analog 5**	TCGCGCTC	**Analog 5**	0.77116	0.77116	0.71457	0.65062	0.46787	0.46962	1.00000
**20**	**Origin**	ACAGGACG	**Origin**	1.00000	0.56388	0.68343	0.85459	0.70049	0.70418	0.68343
	**Represent**	GCAGCACG	**Represent**	0.56388	1.00000	0.86795	0.71738	0.25469	0.38921	0.86795
	**Analog 1**	ACAGCACG	**Analog 1**	0.68343	0.86795	1.00000	0.56076	0.36730	0.49012	1.00000
	**Analog 2**	GCAGGACG	**Analog 2**	0.85459	0.71738	0.56076	1.00000	0.56225	0.58475	0.56076
	**Analog 3**	ACAGGATG	**Analog 3**	0.70049	0.25469	0.36730	0.56225	1.00000	0.50531	0.36730
	**Analog 4**	ACAGGGCG	**Analog 4**	0.70418	0.38921	0.49012	0.58475	0.50531	1.00000	0.49012
	**Analog 5**	ACAGCACG	**Analog 5**	0.68343	0.86795	1.00000	0.56076	0.36730	0.49012	1.00000
**21**	**Origin**	GATAGATA	**Origin**	1.00000	0.71163	0.65953	0.89623	0.74269	0.74269	0.71163
	**Represent**	GATAGACA	**Represent**	0.71163	1.00000	0.38771	0.65591	0.50820	0.50820	1.00000
	**Analog 1**	GATATATA	**Analog 1**	0.65953	0.38771	1.00000	0.57130	0.41460	0.41460	0.38771
	**Analog 2**	GATAGATC	**Analog 2**	0.89623	0.65591	0.57130	1.00000	0.66028	0.66028	0.65591
	**Analog 3**	GAGAGATA	**Analog 3**	0.74269	0.50820	0.41460	0.66028	1.00000	1.00000	0.50820
	**Analog 4**	GAGAGATA	**Analog 4**	0.74269	0.50820	0.41460	0.66028	1.00000	1.00000	0.50820
	**Analog 5**	GATAGACA	**Analog 5**	0.71163	1.00000	0.38771	0.65591	0.50820	0.50820	1.00000
**22**	**Origin**	CTTCGACT	**Origin**	1.00000	0.42884	0.89781	0.79180	0.72088	0.74079	0.79180

	**Represent**	GTGCGAAT	**Represent**	0.42884	1.00000	0.52960	0.59182	0.75581	0.42780	0.59182
	**Analog 1**	GTTCGACT	**Analog 1**	0.89781	0.52960	1.00000	0.68632	0.63299	0.64163	0.68632
	**Analog 2**	CTTCGAAT	**Analog 2**	0.79180	0.59182	0.68632	1.00000	0.52577	0.79122	1.00000
	**Analog 3**	CTGCGACT	**Analog 3**	0.72088	0.75581	0.63299	0.52577	1.00000	0.49103	0.52577
	**Analog 4**	CTTCGAGT	**Analog 4**	0.74079	0.42780	0.64163	0.79122	0.49103	1.00000	0.79122
	**Analog 5**	CTTCGAAT	**Analog 5**	0.79180	0.59182	0.68632	1.00000	0.52577	0.79122	1.00000
**23**	**Origin**	TAAGTAGC	**Origin**	1.00000	0.79072	0.72552	0.91881	0.67851	0.79072	0.66809
	**Represent**	TCAGTAGC	**Represent**	0.79072	1.00000	0.53129	0.73750	0.49654	1.00000	0.55208
	**Analog 1**	TAAGTAGG	**Analog 1**	0.72552	0.53129	1.00000	0.65867	0.38508	0.53129	0.41679
	**Analog 2**	CAAGTAGC	**Analog 2**	0.91881	0.73750	0.65867	1.00000	0.61580	0.73750	0.61205
	**Analog 3**	TAACTAGC	**Analog 3**	0.67851	0.49654	0.38508	0.61580	1.00000	0.49654	0.46750
	**Analog 4**	TCAGTAGC	**Analog 4**	0.79072	1.00000	0.53129	0.73750	0.49654	1.00000	0.55208
	**Analog 5**	TACGTAGC	**Analog 5**	0.66809	0.55208	0.41679	0.61205	0.46750	0.55208	1.00000
**24**	**Origin**	GTGCATGT	**Origin**	1.00000	1.00000	0.90440	0.74236	0.78597	0.62263	0.62263
	**Represent**	GTGCATGT	**Represent**	1.00000	1.00000	0.90440	0.74236	0.78597	0.62263	0.62263
	**Analog 1**	CTGCATGT	**Analog 1**	0.90440	0.90440	1.00000	0.65418	0.68087	0.51553	0.51553
	**Analog 2**	GTGCACGT	**Analog 2**	0.74236	0.74236	0.65418	1.00000	0.63651	0.39135	0.39135
	**Analog 3**	GTGCATTT	**Analog 3**	0.78597	0.78597	0.68087	0.63651	1.00000	0.36702	0.36702
	**Analog 4**	GTGGATGT	**Analog 4**	0.62263	0.62263	0.51553	0.39135	0.36702	1.00000	1.00000
	**Analog 5**	GTGGATGT	**Analog 5**	0.62263	0.62263	0.51553	0.39135	0.36702	1.00000	1.00000
**25**	**Origin**	ACCAACCT	**Origin**	1.00000	1.00000	0.64501	0.72805	0.64613	0.72893	0.74187
	**Represent**	ACCAACCT	**Represent**	1.00000	1.00000	0.64501	0.72805	0.64613	0.72893	0.74187
	**Analog 1**	ACGAACCT	**Analog 1**	0.64501	0.64501	1.00000	0.49538	0.44880	0.34991	0.37211
	**Analog 2**	ACCGACCT	**Analog 2**	0.72805	0.72805	0.49538	1.00000	0.41277	0.46067	0.47933
	**Analog 3**	AGCAACCT	**Analog 3**	0.64613	0.64613	0.44880	0.41277	1.00000	0.35239	0.37442
	**Analog 4**	ACCAATCT	**Analog 4**	0.72893	0.72893	0.34991	0.46067	0.35239	1.00000	0.62409
	**Analog 5**	ACCAACTT	**Analog 5**	0.74187	0.74187	0.37211	0.47933	0.37442	0.62409	1.00000
**26**	**Origin**	CCCATACA	**Origin**	1.00000	0.31347	0.72097	0.85243	0.49784	0.78081	0.75825
	**Represent**	CGCATCCA	**Represent**	0.31347	1.00000	0.27563	0.20433	0.79597	0.18268	0.56674
	**Analog 1**	CCCATGCA	**Analog 1**	0.72097	0.27563	1.00000	0.59844	0.29582	0.62750	0.66425
	**Analog 2**	CCCATACC	**Analog 2**	0.85243	0.20433	0.59844	1.00000	0.37540	0.75604	0.62781
	**Analog 3**	CGCATACA	**Analog 3**	0.49784	0.79597	0.29582	0.37540	1.00000	0.24956	0.30950
	**Analog 4**	CCCATATA	**Analog 4**	0.78081	0.18268	0.62750	0.75604	0.24956	1.00000	0.66560
	**Analog 5**	CCCATCCA	**Analog 5**	0.75825	0.56674	0.66425	0.62781	0.30950	0.66560	1.00000
**27**	**Origin**	GCCTGCTG	**Origin**	1.00000	0.74216	0.80660	0.73720	0.69537	0.74216	0.79637
	**Represent**	GCCTGCGG	**Represent**	0.74216	1.00000	0.74263	0.47695	0.45256	1.00000	0.54678
	**Analog 1**	GCCTGCAG	**Analog 1**	0.80660	0.74263	1.00000	0.52149	0.49261	0.74263	0.59335
	**Analog 2**	GCCTTCTG	**Analog 2**	0.73720	0.47695	0.52149	1.00000	0.77226	0.47695	0.49136

	**Analog 3**	GCCTCCTG	**Analog 3**	0.69537	0.45256	0.49261	0.77226	1.00000	0.45256	0.46289
	**Analog 4**	GCCTGCGG	**Analog 4**	0.74216	1.00000	0.74263	0.47695	0.45256	1.00000	0.54678
	**Analog 5**	GCATGCTG	**Analog 5**	0.79637	0.54678	0.59335	0.49136	0.46289	0.54678	1.00000
**28**	**Origin**	GAATGGTC	**Origin**	1.00000	0.52307	0.72551	0.78761	0.90792	0.90783	0.88057
	**Represent**	CAAGGGTT	**Represent**	0.52307	1.00000	0.81369	0.41688	0.53289	0.60075	0.64089
	**Analog 1**	GAAGGGTC	**Analog 1**	0.72551	0.81369	1.00000	0.52154	0.63481	0.63447	0.61635
	**Analog 2**	GTATGGTC	**Analog 2**	0.78761	0.41688	0.52154	1.00000	0.80721	0.68835	0.77714
	**Analog 3**	AAATGGTC	**Analog 3**	0.90792	0.53289	0.63481	0.80721	1.00000	0.81107	0.89819
	**Analog 4**	GAATGGTT	**Analog 4**	0.90783	0.60075	0.63447	0.68835	0.81107	1.00000	0.78700
	**Analog 5**	CAATGGTC	**Analog 5**	0.88057	0.64089	0.61635	0.77714	0.89819	0.78700	1.00000
**29**	**Origin**	AACGTGGC	**Origin**	1.00000	0.65532	0.77359	0.93481	0.74882	0.70623	0.74002
	**Represent**	CACGCGGC	**Represent**	0.65532	1.00000	0.52153	0.71771	0.62513	0.94801	0.48344
	**Analog 1**	AACGTTGC	**Analog 1**	0.77359	0.52153	1.00000	0.71097	0.61088	0.57228	0.46885
	**Analog 2**	CACGTGGC	**Analog 2**	0.93481	0.71771	0.71097	1.00000	0.69136	0.65230	0.67596
	**Analog 3**	AACGGGGC	**Analog 3**	0.74882	0.62513	0.61088	0.69136	1.00000	0.67358	0.57308
	**Analog 4**	AACGCGGC	**Analog 4**	0.70623	0.94801	0.57228	0.65230	0.67358	1.00000	0.53550
	**Analog 5**	AACATGGC	**Analog 5**	0.74002	0.48344	0.46885	0.67596	0.57308	0.53550	1.00000
**30**	**Origin**	GGTGGGAT	**Origin**	1.00000	0.50237	0.76372	0.72780	0.74468	0.76372	0.82200
	**Represent**	GTTGGGCT	**Represent**	0.50237	1.00000	0.77964	0.73609	0.54698	0.77964	0.48253
	**Analog 1**	GGTGGGCT	**Analog 1**	0.76372	0.77964	1.00000	0.50842	0.52037	1.00000	0.60757
	**Analog 2**	GTTGGGAT	**Analog 2**	0.72780	0.73609	0.50842	1.00000	0.79231	0.50842	0.69989
	**Analog 3**	GATGGGAT	**Analog 3**	0.74468	0.54698	0.52037	0.79231	1.00000	0.52037	0.71642
	**Analog 4**	GGTGGGCT	**Analog 4**	0.76372	0.77964	1.00000	0.50842	0.52037	1.00000	0.60757
	**Analog 5**	CGTGGGAT	**Analog 5**	0.82200	0.48253	0.60757	0.69989	0.71642	0.60757	1.00000
**Mismatch-2**		Sequence	**Pearson's correlation coefficient**	**Origin**	**Origin**	**Represent**	**Analog 1**	**Analog 2**	**Analog 3**	**Analog 4**
**1**	**Origin**	GATTGGTG	**Origin**	1.00000	0.68779	0.65089	0.50745	0.67716	0.77902	0.57596
	**Represent**	GATCGGTG	**Represent**	0.68779	1.00000	0.44404	0.44904	0.39399	0.49585	0.90287
	**Analog 1**	GGATGGTG	**Analog 1**	0.65089	0.44404	1.00000	0.24374	0.52885	0.62553	0.33700
	**Analog 2**	GATGAGTG	**Analog 2**	0.50745	0.44904	0.24374	1.00000	0.16868	0.29739	0.33096
	**Analog 3**	GTTTGGTT	**Analog 3**	0.67716	0.39399	0.52885	0.16868	1.00000	0.65692	0.39654
	**Analog 4**	TCTTGGTG	**Analog 4**	0.77902	0.49585	0.62553	0.29739	0.65692	1.00000	0.38577
	**Analog 5**	GATCGGTC	**Analog 5**	0.57596	0.90287	0.33700	0.33096	0.39654	0.38577	1.00000
**2**	**Origin**	GTGCATAC	**Origin**	1.00000	0.67096	0.71278	0.44208	0.51579	0.50162	0.55473
	**Represent**	TTGCATCC	**Represent**	0.67096	1.00000	0.51398	0.42146	0.35509	0.78602	0.48429
	**Analog 1**	CAGCATAC	**Analog 1**	0.71278	0.51398	1.00000	0.44842	0.30537	0.64908	0.56288
	**Analog 2**	GGGCATGC	**Analog 2**	0.44208	0.42146	0.44842	1.00000	0.06574	0.41660	0.76597
	**Analog 3**	TTGTATAC	**Analog 3**	0.51579	0.35509	0.30537	0.06574	1.00000	0.07855	0.14437

	**Analog 4**	GAGCATCC	**Analog 4**	0.50162	0.78602	0.64908	0.41660	0.07855	1.00000	0.47880
	**Analog 5**	GGGCATAG	**Analog 5**	0.55473	0.48429	0.56288	0.76597	0.14437	0.47880	1.00000
**3**	**Origin**	TGCCTCGT	**Origin**	1.00000	0.03838	0.57856	0.52215	0.41158	0.67911	0.48031
	**Represent**	GGGCGCCC	**Represent**	0.03838	1.00000	0.15643	0.47361	0.17945	0.30648	0.37714
	**Analog 1**	GGTCTCGT	**Analog 1**	0.57856	0.15643	1.00000	0.69898	0.21095	0.22444	0.33280
	**Analog 2**	GGGCTCGT	**Analog 2**	0.52215	0.47361	0.69898	1.00000	0.18842	0.20277	0.30337
	**Analog 3**	TACCTCCT	**Analog 3**	0.41158	0.17945	0.21095	0.18842	1.00000	0.62594	0.20217
	**Analog 4**	TGCCTCCC	**Analog 4**	0.67911	0.30648	0.22444	0.20277	0.62594	1.00000	0.16836
	**Analog 5**	TTCCGCGT	**Analog 5**	0.48031	0.37714	0.33280	0.30337	0.20217	0.16836	1.00000
**4**	**Origin**	AGCACCCC	**Origin**	1.00000	0.35062	0.38303	0.60747	0.45165	0.49719	0.61190
	**Represent**	GGCGCGCC	**Represent**	0.35062	1.00000	0.51940	0.56873	0.53680	0.14671	0.24572
	**Analog 1**	AACACGCC	**Analog 1**	0.38303	0.51940	1.00000	0.04542	-0.00743	0.17343	0.24818
	**Analog 2**	AGCGCCCA	**Analog 2**	0.60747	0.56873	0.04542	1.00000	0.80006	0.33875	0.31989
	**Analog 3**	AGCGCCAC	**Analog 3**	0.45165	0.53680	-0.00743	0.80006	1.00000	0.16826	0.15534
	**Analog 4**	ACCTCCCC	**Analog 4**	0.49719	0.14671	0.17343	0.33875	0.16826	1.00000	0.44922
	**Analog 5**	GGGACCCC	**Analog 5**	0.61190	0.24572	0.24818	0.31989	0.15534	0.44922	1.00000
**5**	**Origin**	GGAGGCGG	**Origin**	1.00000	0.40847	0.85233	0.61215	0.68633	0.49344	0.80942
	**Represent**	GGCGGGGG	**Represent**	0.40847	1.00000	0.28799	0.42375	0.25019	0.59060	0.19932
	**Analog 1**	ATAGGCGG	**Analog 1**	0.85233	0.28799	1.00000	0.51767	0.58407	0.29202	0.76665
	**Analog 2**	GGCCGCGG	**Analog 2**	0.61215	0.42375	0.51767	1.00000	0.61126	0.15077	0.42598
	**Analog 3**	GGGAGCGG	**Analog 3**	0.68633	0.25019	0.58407	0.61126	1.00000	0.16782	0.47732
	**Analog 4**	GGAGGGGC	**Analog 4**	0.49344	0.59060	0.29202	0.15077	0.16782	1.00000	0.39100
	**Analog 5**	AGAGGCGT	**Analog 5**	0.80942	0.19932	0.76665	0.42598	0.47732	0.39100	1.00000
**6**	**Origin**	TCCCGGTG	**Origin**	1.00000	0.41071	0.51265	0.60301	0.32593	0.51500	0.84442
	**Represent**	TCGCGGCG	**Represent**	0.41071	1.00000	0.65035	0.09815	0.48337	0.16314	0.35306
	**Analog 1**	TTCCGGCG	**Analog 1**	0.51265	0.65035	1.00000	0.17788	-0.01053	0.48820	0.51341
	**Analog 2**	TCCCTGTC	**Analog 2**	0.60301	0.09815	0.17788	1.00000	0.25740	0.18372	0.60342
	**Analog 3**	TCGCAGTG	**Analog 3**	0.32593	0.48337	-0.01053	0.25740	1.00000	0.07414	0.16662
	**Analog 4**	TTCCGCTG	**Analog 4**	0.51500	0.16314	0.48820	0.18372	0.07414	1.00000	0.42637
	**Analog 5**	ACCCGGTC	**Analog 5**	0.84442	0.35306	0.51341	0.60342	0.16662	0.42637	1.00000
**7**	**Origin**	AAACCGCG	**Origin**	1.00000	0.60783	0.61078	0.74443	0.77953	0.24814	0.75832
	**Represent**	TAACGGCG	**Represent**	0.60783	1.00000	0.43692	0.50369	0.45691	0.60990	0.41923
	**Analog 1**	TAACCACG	**Analog 1**	0.61078	0.43692	1.00000	0.34117	0.43889	0.16526	0.39614
	**Analog 2**	CAATCGCG	**Analog 2**	0.74443	0.50369	0.34117	1.00000	0.65087	0.05917	0.62755
	**Analog 3**	TACCCGCG	**Analog 3**	0.77953	0.45691	0.43889	0.65087	1.00000	0.02599	0.73546
	**Analog 4**	AAACGGTG	**Analog 4**	0.24814	0.60990	0.16526	0.05917	0.02599	1.00000	-0.00501
	**Analog 5**	ACTCCGCG	**Analog 5**	0.75832	0.41923	0.39614	0.62755	0.73546	-0.00501	1.00000
**8**	**Origin**	GTGGTAAG	**Origin**	1.00000	0.21077	0.75771	0.64640	0.78405	0.43906	0.52347

	**Represent**	CTGCTGAC	**Represent**	0.21077	1.00000	0.32472	0.30035	0.16803	0.42625	0.34303
	**Analog 1**	GTGGTTAC	**Analog 1**	0.75771	0.32472	1.00000	0.64308	0.72698	0.25053	0.25271
	**Analog 2**	GTGGTGTG	**Analog 2**	0.64640	0.30035	0.64308	1.00000	0.70047	0.18422	0.18130
	**Analog 3**	GTGGTATC	**Analog 3**	0.78405	0.16803	0.72698	0.70047	1.00000	0.25372	0.27125
	**Analog 4**	GTGCGAAG	**Analog 4**	0.43906	0.42625	0.25053	0.18422	0.25372	1.00000	0.38353
	**Analog 5**	CTGATAAG	**Analog 5**	0.52347	0.34303	0.25271	0.18130	0.27125	0.38353	1.00000
**9**	**Origin**	CGCAACAA	**Origin**	1.00000	0.34915	0.63166	0.56163	0.60071	0.60770	0.46347
	**Represent**	CGAGACGA	**Represent**	0.34915	1.00000	0.22146	0.19036	0.48417	0.45283	0.38386
	**Analog 1**	CGCTAAAA	**Analog 1**	0.63166	0.22146	1.00000	0.44045	0.29475	0.52498	0.57539
	**Analog 2**	CGCAGCCA	**Analog 2**	0.56163	0.19036	0.44045	1.00000	0.37489	0.42251	0.21743
	**Analog 3**	AGCAACGA	**Analog 3**	0.60071	0.48417	0.29475	0.37489	1.00000	0.40668	0.09741
	**Analog 4**	CGCGACTA	**Analog 4**	0.60770	0.45283	0.52498	0.42251	0.40668	1.00000	0.24345
	**Analog 5**	CGAAAAAA	**Analog 5**	0.46347	0.38386	0.57539	0.21743	0.09741	0.24345	1.00000
**10**	**Origin**	GGTGACGC	**Origin**	1.00000	0.74247	0.61711	0.49341	0.53704	0.70005	0.67281
	**Represent**	GGTGCCGC	**Represent**	0.74247	1.00000	0.87777	0.33669	0.49897	0.51118	0.50896
	**Analog 1**	CGTGCCGC	**Analog 1**	0.61711	0.87777	1.00000	0.20250	0.39212	0.37547	0.39219
	**Analog 2**	GGTGAGAC	**Analog 2**	0.49341	0.33669	0.20250	1.00000	0.11488	0.30559	0.11993
	**Analog 3**	GGCGTCGC	**Analog 3**	0.53704	0.49897	0.39212	0.11488	1.00000	0.38381	0.72355
	**Analog 4**	GGTAACGT	**Analog 4**	0.70005	0.51118	0.37547	0.30559	0.38381	1.00000	0.56293
	**Analog 5**	GGCTACGC	**Analog 5**	0.67281	0.50896	0.39219	0.11993	0.72355	0.56293	1.00000
**11**	**Origin**	ATCCCTGT	**Origin**	1.00000	0.14992	0.58778	0.53981	0.53120	0.30531	0.59474
	**Represent**	CTCGCCGG	**Represent**	0.14992	1.00000	0.16714	0.27583	0.19743	0.58083	0.14180
	**Analog 1**	ATTCCTGG	**Analog 1**	0.58778	0.16714	1.00000	0.24160	0.40048	0.06468	0.19837
	**Analog 2**	ATCCCCCT	**Analog 2**	0.53981	0.27583	0.24160	1.00000	0.28943	0.14577	0.25582
	**Analog 3**	ATCCGTGG	**Analog 3**	0.53120	0.19743	0.40048	0.28943	1.00000	0.11985	0.82414
	**Analog 4**	ATCGCAGT	**Analog 4**	0.30531	0.58083	0.06468	0.14577	0.11985	1.00000	0.17724
	**Analog 5**	CTCCGTGT	**Analog 5**	0.59474	0.14180	0.19837	0.25582	0.82414	0.17724	1.00000
**12**	**Origin**	GCGTAGAG	**Origin**	1.00000	0.56756	0.77963	0.58819	0.53958	0.33426	0.56698
	**Represent**	GCGCAGTG	**Represent**	0.56756	1.00000	0.45845	0.60051	0.36743	0.46587	0.44971
	**Analog 1**	GCGTTGAT	**Analog 1**	0.77963	0.45845	1.00000	0.58341	0.53750	0.12007	0.35349
	**Analog 2**	GCGTCGTG	**Analog 2**	0.58819	0.60051	0.58341	1.00000	0.47859	-0.00859	0.20137
	**Analog 3**	GCGTGGCG	**Analog 3**	0.53958	0.36743	0.53750	0.47859	1.00000	-0.01275	0.18123
	**Analog 4**	GAGCAGAG	**Analog 4**	0.33426	0.46587	0.12007	-0.00859	-0.01275	1.00000	0.44529
	**Analog 5**	GCACAGAG	**Analog 5**	0.56698	0.44971	0.35349	0.20137	0.18123	0.44529	1.00000
**13**	**Origin**	TGAATGGA	**Origin**	1.00000	0.33531	0.33219	0.58504	0.36472	0.41120	0.45581
	**Represent**	TAAATGCA	**Represent**	0.33531	1.00000	0.87100	0.18495	0.30198	0.42408	0.25584
	**Analog 1**	TTAATGCA	**Analog 1**	0.33219	0.87100	1.00000	0.18770	0.14327	0.57800	0.25822
	**Analog 2**	TGCATGGT	**Analog 2**	0.58504	0.18495	0.18770	1.00000	0.07672	0.23521	0.40322

	**Analog 3**	TAAATTGA	**Analog 3**	0.36472	0.30198	0.14327	0.07672	1.00000	0.20229	0.02979
	**Analog 4**	TTAATGTA	**Analog 4**	0.41120	0.42408	0.57800	0.23521	0.20229	1.00000	0.58281
	**Analog 5**	TGTATGTA	**Analog 5**	0.45581	0.25584	0.25822	0.40322	0.02979	0.58281	1.00000
**14**	**Origin**	GGCTTCTA	**Origin**	1.00000	0.80098	0.63811	0.59399	0.76193	0.51922	0.27126
	**Represent**	CGCTTCTA	**Represent**	0.80098	1.00000	0.83608	0.81062	0.57691	0.49388	0.25559
	**Analog 1**	CGCTTCAA	**Analog 1**	0.63811	0.83608	1.00000	0.73372	0.44303	0.32836	0.11086
	**Analog 2**	CGCTTTTA	**Analog 2**	0.59399	0.81062	0.73372	1.00000	0.36703	0.25225	0.02745
	**Analog 3**	GGCATCTT	**Analog 3**	0.76193	0.57691	0.44303	0.36703	1.00000	0.27353	0.44117
	**Analog 4**	ACCTTCTA	**Analog 4**	0.51922	0.49388	0.32836	0.25225	0.27353	1.00000	0.58299
	**Analog 5**	GCCATCTA	**Analog 5**	0.27126	0.25559	0.11086	0.02745	0.44117	0.58299	1.00000
**15**	**Origin**	TACGGAGC	**Origin**	1.00000	0.41688	0.72145	0.68853	0.68806	0.49378	0.65666
	**Represent**	TAGGGCGC	**Represent**	0.41688	1.00000	0.36559	0.16401	0.73426	0.52471	0.18049
	**Analog 1**	GACGGGGC	**Analog 1**	0.72145	0.36559	1.00000	0.51542	0.62726	0.23572	0.41761
	**Analog 2**	AACGGAAC	**Analog 2**	0.68853	0.16401	0.51542	1.00000	0.53270	0.35593	0.56589
	**Analog 3**	AACGGCGC	**Analog 3**	0.68806	0.73426	0.62726	0.53270	1.00000	0.22555	0.40183
	**Analog 4**	TAGGGAGA	**Analog 4**	0.49378	0.52471	0.23572	0.35593	0.22555	1.00000	0.52252
	**Analog 5**	TCCGGAGA	**Analog 5**	0.65666	0.18049	0.41761	0.56589	0.40183	0.52252	1.00000
**16**	**Origin**	ATCTGCGA	**Origin**	1.00000	0.78271	0.51974	0.42007	0.47262	0.64185	0.32496
	**Represent**	ATCAGCGA	**Represent**	0.78271	1.00000	0.39602	0.20037	0.43428	0.51873	0.58989
	**Analog 1**	AACTTCGA	**Analog 1**	0.51974	0.39602	1.00000	0.12976	0.42181	0.76470	0.10550
	**Analog 2**	ATCTGGGC	**Analog 2**	0.42007	0.20037	0.12976	1.00000	0.07504	0.25524	0.23288
	**Analog 3**	ATGTACGA	**Analog 3**	0.47262	0.43428	0.42181	0.07504	1.00000	0.46392	0.14419
	**Analog 4**	CTCTTCGA	**Analog 4**	0.64185	0.51873	0.76470	0.25524	0.46392	1.00000	0.25672
	**Analog 5**	ATCAGAGA	**Analog 5**	0.32496	0.58989	0.10550	0.23288	0.14419	0.25672	1.00000
**17**	**Origin**	CATAGAGT	**Origin**	1.00000	0.21552	0.48635	0.50266	0.53607	0.51804	0.52118
	**Represent**	CGCAGACT	**Represent**	0.21552	1.00000	0.49791	0.18023	0.32934	0.45263	0.19070
	**Analog 1**	GATAGACT	**Analog 1**	0.48635	0.49791	1.00000	0.32512	0.24706	0.23653	0.33119
	**Analog 2**	CCTAGAGG	**Analog 2**	0.50266	0.18023	0.32512	1.00000	0.60419	0.34211	0.46730
	**Analog 3**	CCCAGAGT	**Analog 3**	0.53607	0.32934	0.24706	0.60419	1.00000	0.41181	0.37550
	**Analog 4**	CGGAGAGT	**Analog 4**	0.51804	0.45263	0.23653	0.34211	0.41181	1.00000	0.36203
	**Analog 5**	CTTAGAGC	**Analog 5**	0.52118	0.19070	0.33119	0.46730	0.37550	0.36203	1.00000
**18**	**Origin**	CTCGGTCA	**Origin**	1.00000	0.62450	0.56625	0.73872	0.44279	0.64189	0.47586
	**Represent**	CCCGGTCG	**Represent**	0.62450	1.00000	0.81254	0.53397	0.40025	0.40138	0.29792
	**Analog 1**	CCCGGTAA	**Analog 1**	0.56625	0.81254	1.00000	0.69572	0.12211	0.39854	0.33905
	**Analog 2**	GTCGGTAA	**Analog 2**	0.73872	0.53397	0.69572	1.00000	0.25616	0.54181	0.41029
	**Analog 3**	CTCCGTCG	**Analog 3**	0.44279	0.40025	0.12211	0.25616	1.00000	0.14958	0.21761
	**Analog 4**	ATCGGGCA	**Analog 4**	0.64189	0.40138	0.39854	0.54181	0.14958	1.00000	0.19068
	**Analog 5**	CTAGGTGA	**Analog 5**	0.47586	0.29792	0.33905	0.41029	0.21761	0.19068	1.00000

**19**	**Origin**	AGTTTTTA	**Origin**	1.00000	-0.02111	0.34988	0.32100	0.56421	0.48464	0.30285
	**Represent**	ACTGGTCA	**Represent**	-0.02111	1.00000	0.56727	-0.08703	-0.02794	0.53745	0.28025
	**Analog 1**	ACTTTTCA	**Analog 1**	0.34988	0.56727	1.00000	0.19948	0.35627	-0.02001	0.47085
	**Analog 2**	AATTTGTA	**Analog 2**	0.32100	-0.08703	0.19948	1.00000	0.30128	-0.01130	0.06391
	**Analog 3**	ATTTTTTC	**Analog 3**	0.56421	-0.02794	0.35627	0.30128	1.00000	0.10971	0.28151
	**Analog 4**	AGTGGTTA	**Analog 4**	0.48464	0.53745	-0.02001	-0.01130	0.10971	1.00000	0.11937
	**Analog 5**	ACTCTTTA	**Analog 5**	0.30285	0.28025	0.47085	0.06391	0.28151	0.11937	1.00000
**20**	**Origin**	GCCATCTA	**Origin**	1.00000	0.63611	0.44015	0.55237	0.48212	0.46445	0.54852
	**Represent**	GCCAACAA	**Represent**	0.63611	1.00000	0.48537	0.38276	0.53243	0.47691	0.43000
	**Analog 1**	GCGAACTA	**Analog 1**	0.44015	0.48537	1.00000	0.06821	0.68445	0.65815	0.25647
	**Analog 2**	CCCATTTA	**Analog 2**	0.55237	0.38276	0.06821	1.00000	0.07094	0.09347	0.49455
	**Analog 3**	GCAAACTA	**Analog 3**	0.48212	0.53243	0.68445	0.07094	1.00000	0.31048	0.28281
	**Analog 4**	GCGATCAA	**Analog 4**	0.46445	0.47691	0.65815	0.09347	0.31048	1.00000	0.24132
	**Analog 5**	GCCCTTTA	**Analog 5**	0.54852	0.43000	0.25647	0.49455	0.28281	0.24132	1.00000
**21**	**Origin**	GATTTCAA	**Origin**	1.00000	0.37997	0.62283	0.60415	0.31086	0.55137	0.38115
	**Represent**	GTTTGCAA	**Represent**	0.37997	1.00000	0.46258	0.19317	0.52864	0.19361	0.48020
	**Analog 1**	GTATTCAA	**Analog 1**	0.62283	0.46258	1.00000	0.47825	-0.00148	0.26611	0.48874
	**Analog 2**	GAACTCAA	**Analog 2**	0.60415	0.19317	0.47825	1.00000	0.09067	0.13127	0.06473
	**Analog 3**	GATTGCCA	**Analog 3**	0.31086	0.52864	-0.00148	0.09067	1.00000	0.15598	0.04087
	**Analog 4**	AATTTTAA	**Analog 4**	0.55137	0.19361	0.26611	0.13127	0.15598	1.00000	0.44786
	**Analog 5**	GTTTTAAA	**Analog 5**	0.38115	0.48020	0.48874	0.06473	0.04087	0.44786	1.00000
**22**	**Origin**	ACGAACTG	**Origin**	1.00000	0.48302	0.64857	0.60687	0.38989	0.73153	0.48302
	**Represent**	ACCCACTG	**Represent**	0.48302	1.00000	0.13872	0.34782	0.51323	0.23923	1.00000
	**Analog 1**	TCGAAGTG	**Analog 1**	0.64857	0.13872	1.00000	0.38906	0.27000	0.46328	0.13872
	**Analog 2**	ACGCTCTG	**Analog 2**	0.60687	0.34782	0.38906	1.00000	0.20890	0.37173	0.34782
	**Analog 3**	ACCAATTG	**Analog 3**	0.38989	0.51323	0.27000	0.20890	1.00000	0.20594	0.51323
	**Analog 4**	ACGAACCT	**Analog 4**	0.73153	0.23923	0.46328	0.37173	0.20594	1.00000	0.23923
	**Analog 5**	ACCCACTG	**Analog 5**	0.48302	1.00000	0.13872	0.34782	0.51323	0.23923	1.00000
**23**	**Origin**	ATTCCACT	**Origin**	1.00000	0.31159	0.63972	0.33882	0.78137	0.52451	0.45195
	**Represent**	TGTGCACG	**Represent**	0.31159	1.00000	0.13446	0.56220	0.53287	0.53059	0.31767
	**Analog 1**	CTTCCAGT	**Analog 1**	0.63972	0.13446	1.00000	0.27887	0.49313	0.26322	0.21579
	**Analog 2**	ATTGCATT	**Analog 2**	0.33882	0.56220	0.27887	1.00000	0.16977	0.32620	0.09578
	**Analog 3**	TGTCCACT	**Analog 3**	0.78137	0.53287	0.49313	0.16977	1.00000	0.34235	0.28946
	**Analog 4**	ATTTCACG	**Analog 4**	0.52451	0.53059	0.26322	0.32620	0.34235	1.00000	0.53275
	**Analog 5**	ATTCGACG	**Analog 5**	0.45195	0.31767	0.21579	0.09578	0.28946	0.53275	1.00000
**24**	**Origin**	GTGCATCT	**Origin**	1.00000	0.31749	0.65722	0.67627	0.68540	0.37725	0.76298
	**Represent**	TCGCCGCT	**Represent**	0.31749	1.00000	0.52167	0.48771	0.59524	0.23552	0.19784
	**Analog 1**	CTGCAGCT	**Analog 1**	0.65722	0.52167	1.00000	0.49511	0.48399	0.12008	0.51433

	**Analog 2**	TTGCCTCT	**Analog 2**	0.67627	0.48771	0.49511	1.00000	0.49734	0.55693	0.45145
	**Analog 3**	TCGCATCT	**Analog 3**	0.68540	0.59524	0.48399	0.49734	1.00000	0.19258	0.46675
	**Analog 4**	GTCCCTCT	**Analog 4**	0.37725	0.23552	0.12008	0.55693	0.19258	1.00000	0.14959
	**Analog 5**	GTGCATGA	**Analog 5**	0.76298	0.19784	0.51433	0.45145	0.46675	0.14959	1.00000
**25**	**Origin**	TGTCTATC	**Origin**	1.00000	0.03436	0.45316	0.36904	0.46415	0.41380	0.39477
	**Represent**	GGGCAACC	**Represent**	0.03436	1.00000	0.23274	0.34578	0.20225	0.40896	-0.01300
	**Analog 1**	TATCAATC	**Analog 1**	0.45316	0.23274	1.00000	0.32922	0.23677	0.10651	0.43135
	**Analog 2**	TATCTACC	**Analog 2**	0.36904	0.34578	0.32922	1.00000	0.16753	0.08015	0.17075
	**Analog 3**	GGACTATC	**Analog 3**	0.46415	0.20225	0.23677	0.16753	1.00000	0.28275	0.20307
	**Analog 4**	TGGCTGTC	**Analog 4**	0.41380	0.40896	0.10651	0.08015	0.28275	1.00000	0.08423
	**Analog 5**	TTTCCATC	**Analog 5**	0.39477	-0.01300	0.43135	0.17075	0.20307	0.08423	1.00000
**26**	**Origin**	CCGTATTT	**Origin**	1.00000	0.07869	0.27126	0.64045	0.69127	0.66067	0.57817
	**Represent**	CGGCACTT	**Represent**	0.07869	1.00000	0.54786	0.31882	-0.00053	0.03281	0.37187
	**Analog 1**	CGGTAATT	**Analog 1**	0.27126	0.54786	1.00000	0.27335	0.19694	0.04705	-0.00496
	**Analog 2**	TCGTACTT	**Analog 2**	0.64045	0.31882	0.27335	1.00000	0.69970	0.38026	0.33143
	**Analog 3**	TCGTATAT	**Analog 3**	0.69127	-0.00053	0.19694	0.69970	1.00000	0.35961	0.54314
	**Analog 4**	CCGGTTTT	**Analog 4**	0.66067	0.03281	0.04705	0.38026	0.35961	1.00000	0.44479
	**Analog 5**	CCGCATAT	**Analog 5**	0.57817	0.37187	-0.00496	0.33143	0.54314	0.44479	1.00000
**27**	**Origin**	TGGCGAAT	**Origin**	1.00000	1.00000	0.50082	0.54350	0.59922	0.49966	0.50434
	**Represent**	TGGCGAAT	**Represent**	1.00000	1.00000	0.50082	0.54350	0.59922	0.49966	0.50434
	**Analog 1**	TGACGACT	**Analog 1**	0.50082	0.50082	1.00000	0.13947	0.78866	0.52241	0.40376
	**Analog 2**	TGGCCAGT	**Analog 2**	0.54350	0.54350	0.13947	1.00000	0.14228	0.35445	-0.00235
	**Analog 3**	TGACGAAG	**Analog 3**	0.59922	0.59922	0.78866	0.14228	1.00000	0.53828	0.51016
	**Analog 4**	TGTCGAGT	**Analog 4**	0.49966	0.49966	0.52241	0.35445	0.53828	1.00000	0.39996
	**Analog 5**	TACCGAAT	**Analog 5**	0.50434	0.50434	0.40376	-0.00235	0.51016	0.39996	1.00000
**28**	**Origin**	ATCCCTTG	**Origin**	1.00000	0.40908	0.43728	0.68990	0.60816	0.68641	0.40205
	**Represent**	ATACCTCG	**Represent**	0.40908	1.00000	0.42070	0.28458	0.09092	0.36174	0.81225
	**Analog 1**	ATACCGTG	**Analog 1**	0.43728	0.42070	1.00000	0.32540	0.07863	0.23114	0.24808
	**Analog 2**	ATCCCATT	**Analog 2**	0.68990	0.28458	0.32540	1.00000	0.49902	0.57935	0.27926
	**Analog 3**	ATCCATTA	**Analog 3**	0.60816	0.09092	0.07863	0.49902	1.00000	0.44225	0.08858
	**Analog 4**	ATCCCTGC	**Analog 4**	0.68641	0.36174	0.23114	0.57935	0.44225	1.00000	0.35550
	**Analog 5**	ATTCCTCG	**Analog 5**	0.40205	0.81225	0.24808	0.27926	0.08858	0.35550	1.00000
**29**	**Origin**	TGATTATT	**Origin**	1.00000	0.63159	0.66777	0.57690	0.39074	0.47446	0.30126
	**Represent**	GGATTATG	**Represent**	0.63159	1.00000	0.79894	0.69502	0.18988	0.63679	0.26581
	**Analog 1**	AGATTATG	**Analog 1**	0.66777	0.79894	1.00000	0.46099	0.19593	0.68531	0.28882
	**Analog 2**	GGATAATT	**Analog 2**	0.57690	0.69502	0.46099	1.00000	0.04272	0.24801	0.09636
	**Analog 3**	TGTTTACT	**Analog 3**	0.39074	0.18988	0.19593	0.04272	1.00000	0.14622	0.55771
	**Analog 4**	TAATTATG	**Analog 4**	0.47446	0.63679	0.68531	0.24801	0.14622	1.00000	0.32771

	**Analog 5**	TTATTACT	**Analog 5**	0.30126	0.26581	0.28882	0.09636	0.55771	0.32771	1.00000
**30**	**Origin**	AATCGGGC	**Origin**	1.00000	0.48080	0.77166	0.62596	0.47166	0.26031	0.65910
	**Represent**	AACCGGAC	**Represent**	0.48080	1.00000	0.44585	0.48877	0.48837	0.36421	0.75930
	**Analog 1**	GATCGGGG	**Analog 1**	0.77166	0.44585	1.00000	0.64463	0.43756	0.20774	0.62287
	**Analog 2**	TATCGGTC	**Analog 2**	0.62596	0.48877	0.64463	1.00000	0.71095	0.22914	0.47409
	**Analog 3**	AAGCGGTC	**Analog 3**	0.47166	0.48837	0.43756	0.71095	1.00000	0.09639	0.47333
	**Analog 4**	AATCAGAC	**Analog 4**	0.26031	0.36421	0.20774	0.22914	0.09639	1.00000	0.09353
	**Analog 5**	AACCGGGT	**Analog 5**	0.65910	0.75930	0.62287	0.47409	0.47333	0.09353	1.00000
**Mismatch-4**		**Sequence**	**Pearson's correlation coefficient**	**Origin**	**Origin**	**Represent**	**Analog 1**	**Analog 2**	**Analog 3**	**Analog 4**
**1**	**Origin**	CTATAAGC	**Origin**	1.00000	-0.02287	0.06836	0.10165	0.10044	0.11527	0.00838
	**Represent**	AAATCGCG	**Represent**	-0.02287	1.00000	0.15242	0.28245	0.08464	0.31083	-0.08313
	**Analog 1**	ACATCATC	**Analog 1**	0.06836	0.15242	1.00000	-0.03350	-0.02942	-0.08772	0.24992
	**Analog 2**	CATTAACG	**Analog 2**	0.10165	0.28245	-0.03350	1.00000	0.10337	0.01472	0.10409
	**Analog 3**	AAAAAAAC	**Analog 3**	0.10044	0.08464	-0.02942	0.10337	1.00000	-0.04137	-0.08809
	**Analog 4**	CTAGAGCA	**Analog 4**	0.11527	0.31083	-0.08772	0.01472	-0.04137	1.00000	-0.08753
	**Analog 5**	ATTTTATC	**Analog 5**	0.00838	-0.08313	0.24992	0.10409	-0.08809	-0.08753	1.00000
**2**	**Origin**	CCCCACTC	**Origin**	1.00000	0.12412	0.12384	0.28981	0.33523	0.11451	0.30723
	**Represent**	GCAGACGC	**Represent**	0.12412	1.00000	0.10190	0.20529	0.23218	0.05615	0.54418
	**Analog 1**	ACTGAGTC	**Analog 1**	0.12384	0.10190	1.00000	-0.06808	0.22746	0.33465	0.03981
	**Analog 2**	CCCAGAGC	**Analog 2**	0.28981	0.20529	-0.06808	1.00000	0.13722	-0.01446	-0.03735
	**Analog 3**	ACCGACCT	**Analog 3**	0.33523	0.23218	0.22746	0.13722	1.00000	0.00980	0.16595
	**Analog 4**	ACACGGTC	**Analog 4**	0.11451	0.05615	0.33465	-0.01446	0.00980	1.00000	0.14828
	**Analog 5**	GCATACTA	**Analog 5**	0.30723	0.54418	0.03981	-0.03735	0.16595	0.14828	1.00000
**3**	**Origin**	TTCGGTGT	**Origin**	1.00000	0.28003	0.44157	0.40304	0.15853	0.08692	0.04161
	**Represent**	TCAGGCGT	**Represent**	0.28003	1.00000	0.25844	0.16638	0.35697	0.32539	-0.06505
	**Analog 1**	TCCGGATC	**Analog 1**	0.44157	0.25844	1.00000	0.21967	0.08963	-0.06343	-0.01348
	**Analog 2**	GTTGGTTA	**Analog 2**	0.40304	0.16638	0.21967	1.00000	-0.01328	-0.06315	0.25999
	**Analog 3**	TCAGATGG	**Analog 3**	0.15853	0.35697	0.08963	-0.01328	1.00000	-0.00573	-0.01889
	**Analog 4**	TGGGTCGT	**Analog 4**	0.08692	0.32539	-0.06343	-0.06315	-0.00573	1.00000	0.14749
	**Analog 5**	GTGGCTTT	**Analog 5**	0.04161	-0.06505	-0.01348	0.25999	-0.01889	0.14749	1.00000
**4**	**Origin**	CTATCGGC	**Origin**	1.00000	0.44042	0.34357	0.10935	0.07397	0.37574	0.33245
	**Represent**	CCACCGGG	**Represent**	0.44042	1.00000	0.22431	0.28105	0.46700	0.19447	0.37183
	**Analog 1**	GAATAGGA	**Analog 1**	0.34357	0.22431	1.00000	-0.09110	0.09155	-0.00757	-0.04872
	**Analog 2**	CTACCCTG	**Analog 2**	0.10935	0.28105	-0.09110	1.00000	-0.07829	-0.02637	0.29388
	**Analog 3**	CCATTAGG	**Analog 3**	0.07397	0.46700	0.09155	-0.07829	1.00000	-0.08671	-0.12182
	**Analog 4**	TTGTCGCA	**Analog 4**	0.37574	0.19447	-0.00757	-0.02637	-0.08671	1.00000	0.38953
	**Analog 5**	CTGCCGAT	**Analog 5**	0.33245	0.37183	-0.04872	0.29388	-0.12182	0.38953	1.00000

**5**	**Origin**	CTGGGGGT	**Origin**	1.00000	0.63423	0.20298	0.48807	0.19202	0.32762	0.00189
	**Represent**	CCGGGGGG	**Represent**	0.63423	1.00000	0.13868	0.43494	0.36095	0.60042	-0.01035
	**Analog 1**	ATTGGTCT	**Analog 1**	0.20298	0.13868	1.00000	0.20636	0.18167	-0.06703	-0.05395
	**Analog 2**	AGGGGGCA	**Analog 2**	0.48807	0.43494	0.20636	1.00000	0.12489	0.15503	0.00173
	**Analog 3**	CCAGGAGA	**Analog 3**	0.19202	0.36095	0.18167	0.12489	1.00000	0.03294	-0.06122
	**Analog 4**	ACGGCGGG	**Analog 4**	0.32762	0.60042	-0.06703	0.15503	0.03294	1.00000	0.38343
	**Analog 5**	CACGCGTT	**Analog 5**	0.00189	-0.01035	-0.05395	0.00173	-0.06122	0.38343	1.00000
**6**	**Origin**	CATTTAAT	**Origin**	1.00000	0.04548	0.23073	0.11512	-0.03743	0.04485	0.15927
	**Represent**	GACTGTAC	**Represent**	0.04548	1.00000	0.35460	0.38113	-0.12791	0.20893	0.30361
	**Analog 1**	AACTTATC	**Analog 1**	0.23073	0.35460	1.00000	-0.06905	0.04451	0.23878	-0.02476
	**Analog 2**	CCTTGTAA	**Analog 2**	0.11512	0.38113	-0.06905	1.00000	0.13938	0.15684	0.02537
	**Analog 3**	CCGCTATT	**Analog 3**	-0.03743	-0.12791	0.04451	0.13938	1.00000	0.13677	-0.08909
	**Analog 4**	AAGTGATT	**Analog 4**	0.04485	0.20893	0.23878	0.15684	0.13677	1.00000	-0.08114
	**Analog 5**	GATGTTAC	**Analog 5**	0.15927	0.30361	-0.02476	0.02537	-0.08909	-0.08114	1.00000
**7**	**Origin**	ATCGTGCT	**Origin**	1.00000	0.08897	0.26531	0.18342	0.24696	0.00114	0.44000
	**Represent**	GCTTTGGT	**Represent**	0.08897	1.00000	0.29648	0.23055	0.39872	0.23272	0.18803
	**Analog 1**	ACTGTGTC	**Analog 1**	0.26531	0.29648	1.00000	0.32031	0.48816	-0.07945	0.11170
	**Analog 2**	TACTTGTT	**Analog 2**	0.18342	0.23055	0.32031	1.00000	0.36497	0.00277	0.24113
	**Analog 3**	GCAGTGTT	**Analog 3**	0.24696	0.39872	0.48816	0.36497	1.00000	-0.07979	0.13364
	**Analog 4**	AGCCAGGT	**Analog 4**	0.00114	0.23272	-0.07945	0.00277	-0.07979	1.00000	-0.05952
	**Analog 5**	GTATTGCG	**Analog 5**	0.44000	0.18803	0.11170	0.24113	0.13364	-0.05952	1.00000
**8**	**Origin**	ACGCGGCG	**Origin**	1.00000	0.31044	0.38035	0.09430	0.22288	0.25412	0.24794
	**Represent**	AGCCGCCG	**Represent**	0.31044	1.00000	0.53242	0.28846	-0.06404	0.30835	0.37037
	**Analog 1**	GGGCGCCT	**Analog 1**	0.38035	0.53242	1.00000	-0.00915	-0.02201	0.12351	0.20524
	**Analog 2**	AGCTGGAG	**Analog 2**	0.09430	0.28846	-0.00915	1.00000	-0.06590	-0.12739	-0.00359
	**Analog 3**	AAGGTGCC	**Analog 3**	0.22288	-0.06404	-0.02201	-0.06590	1.00000	0.00176	-0.07277
	**Analog 4**	ACAATCCG	**Analog 4**	0.25412	0.30835	0.12351	-0.12739	0.00176	1.00000	-0.00486
	**Analog 5**	CCCCGTCA	**Analog 5**	0.24794	0.37037	0.20524	-0.00359	-0.07277	-0.00486	1.00000
**9**	**Origin**	CGACTCGC	**Origin**	1.00000	0.21664	0.06995	0.24368	0.30873	0.41397	0.06845
	**Represent**	CGGGTGGG	**Represent**	0.21664	1.00000	-0.12923	0.27186	0.42644	0.14338	-0.12999
	**Analog 1**	CAACGAAC	**Analog 1**	0.06995	-0.12923	1.00000	-0.08106	-0.13038	0.10529	0.34811
	**Analog 2**	ACAATGGC	**Analog 2**	0.24368	0.27186	-0.08106	1.00000	-0.00154	-0.07654	-0.08172
	**Analog 3**	CTGGTCGG	**Analog 3**	0.30873	0.42644	-0.13038	-0.00154	1.00000	-0.12767	-0.13122
	**Analog 4**	CGACCTTT	**Analog 4**	0.41397	0.14338	0.10529	-0.07654	-0.12767	1.00000	0.14835
	**Analog 5**	CAACATCC	**Analog 5**	0.06845	-0.12999	0.34811	-0.08172	-0.13122	0.14835	1.00000
**10**	**Origin**	GCGAAGCG	**Origin**	1.00000	0.52125	0.28005	0.18944	0.33240	0.24452	0.03821
	**Represent**	GCGAGGAG	**Represent**	0.52125	1.00000	0.23010	0.34851	0.29791	-0.05398	0.58307
	**Analog 1**	GCAAATGT	**Analog 1**	0.28005	0.23010	1.00000	0.31573	-0.00930	0.23348	-0.01903

	**Analog 2**	GCTAGAGG	**Analog 2**	0.18944	0.34851	0.31573	1.00000	-0.01675	-0.05608	0.29239
	**Analog 3**	CCGACCCC	**Analog 3**	0.33240	0.29791	-0.00930	-0.01675	1.00000	0.00191	-0.03215
	**Analog 4**	ACACATCG	**Analog 4**	0.24452	-0.05398	0.23348	-0.05608	0.00191	1.00000	-0.07043
	**Analog 5**	TCTAGGAG	**Analog 5**	0.03821	0.58307	-0.01903	0.29239	-0.03215	-0.07043	1.00000
**11**	**Origin**	TACGTGGC	**Origin**	1.00000	0.37762	0.28060	0.26961	0.34498	0.14773	0.33425
	**Represent**	GGCGTTGG	**Represent**	0.37762	1.00000	0.26603	-0.05054	0.72555	0.27339	0.32302
	**Analog 1**	TCCGAAGT	**Analog 1**	0.28060	0.26603	1.00000	-0.00826	0.26847	0.00067	0.51130
	**Analog 2**	TATAAGGA	**Analog 2**	0.26961	-0.05054	-0.00826	1.00000	-0.11504	0.01137	-0.12754
	**Analog 3**	GGCGTAAC	**Analog 3**	0.34498	0.72555	0.26847	-0.11504	1.00000	-0.04458	0.35227
	**Analog 4**	TACCCTGG	**Analog 4**	0.14773	0.27339	0.00067	0.01137	-0.04458	1.00000	-0.06119
	**Analog 5**	TCCGTCCT	**Analog 5**	0.33425	0.32302	0.51130	-0.12754	0.35227	-0.06119	1.00000
**12**	**Origin**	TGGCGTAT	**Origin**	1.00000	0.00302	0.15162	0.22200	0.31546	0.03633	0.49888
	**Represent**	CAGGCTAG	**Represent**	0.00302	1.00000	0.00801	0.26018	-0.10325	0.43795	0.15401
	**Analog 1**	TGTAGGAG	**Analog 1**	0.15162	0.00801	1.00000	0.12797	0.21255	0.14099	-0.05801
	**Analog 2**	AGGGGCAG	**Analog 2**	0.22200	0.26018	0.12797	1.00000	0.03847	0.02672	0.00049
	**Analog 3**	CGTCGAAA	**Analog 3**	0.31546	-0.10325	0.21255	0.03847	1.00000	0.12225	0.33732
	**Analog 4**	GGTGCTAT	**Analog 4**	0.03633	0.43795	0.14099	0.02672	0.12225	1.00000	-0.13146
	**Analog 5**	CAGCGAGT	**Analog 5**	0.49888	0.15401	-0.05801	0.00049	0.33732	-0.13146	1.00000
**13**	**Origin**	GAGACACA	**Origin**	1.00000	-0.02062	0.03451	0.13717	-0.02988	0.15028	0.08963
	**Represent**	GGGGCCGA	**Represent**	-0.02062	1.00000	0.76212	0.17311	0.21735	0.34909	-0.07375
	**Analog 1**	GGGGCCCT	**Analog 1**	0.03451	0.76212	1.00000	0.17520	0.08769	0.53570	-0.02809
	**Analog 2**	GTAACCTA	**Analog 2**	0.13717	0.17311	0.17520	1.00000	-0.08634	0.19629	-0.01842
	**Analog 3**	GGTAGAGA	**Analog 3**	-0.02988	0.21735	0.08769	-0.08634	1.00000	-0.06289	-0.12897
	**Analog 4**	AGGTCCCA	**Analog 4**	0.15028	0.34909	0.53570	0.19629	-0.06289	1.00000	0.07603
	**Analog 5**	GCACCTCA	**Analog 5**	0.08963	-0.07375	-0.02809	-0.01842	-0.12897	0.07603	1.00000
**14**	**Origin**	CACGTCGT	**Origin**	1.00000	0.61098	0.40092	0.46525	0.34005	0.36409	0.10323
	**Represent**	CGCGTCGG	**Represent**	0.61098	1.00000	0.47449	0.40931	0.22261	0.32181	0.50160
	**Analog 1**	ACCGTAGG	**Analog 1**	0.40092	0.47449	1.00000	0.36267	0.57219	0.33953	-0.02071
	**Analog 2**	ATCGTCCC	**Analog 2**	0.46525	0.40931	0.36267	1.00000	0.20649	0.11616	0.31252
	**Analog 3**	ACCGAGGT	**Analog 3**	0.34005	0.22261	0.57219	0.20649	1.00000	0.28356	-0.05726
	**Analog 4**	ACCCTCGA	**Analog 4**	0.36409	0.32181	0.33953	0.11616	0.28356	1.00000	0.10267
	**Analog 5**	CGCTTCCA	**Analog 5**	0.10323	0.50160	-0.02071	0.31252	-0.05726	0.10267	1.00000
**15**	**Origin**	GCCTCATT	**Origin**	1.00000	-0.00149	0.14758	0.22011	-0.00003	0.41719	0.32902
	**Represent**	CACGCCAT	**Represent**	-0.00149	1.00000	0.06781	-0.02291	0.92027	0.19955	-0.12595
	**Analog 1**	CATTAATT	**Analog 1**	0.14758	0.06781	1.00000	-0.01968	-0.07268	-0.09166	0.10674
	**Analog 2**	CCCCGCTT	**Analog 2**	0.22011	-0.02291	-0.01968	1.00000	-0.02198	0.14883	-0.02239
	**Analog 3**	GACGCCAT	**Analog 3**	-0.00003	0.92027	-0.07268	-0.02198	1.00000	0.20255	-0.12548
	**Analog 4**	GCCGTGTA	**Analog 4**	0.41719	0.19955	-0.09166	0.14883	0.20255	1.00000	0.31049

	**Analog 5**	GCTTTTTA	**Analog 5**	0.32902	-0.12595	0.10674	-0.02239	-0.12548	0.31049	1.00000
**16**	**Origin**	CGCCACAC	**Origin**	1.00000	0.29908	0.18603	0.23431	0.36395	0.26829	0.38340
	**Represent**	CGGCATGG	**Represent**	0.29908	1.00000	0.57437	0.05150	0.16018	0.29270	0.22017
	**Analog 1**	CGGCCATC	**Analog 1**	0.18603	0.57437	1.00000	-0.06139	-0.05230	-0.05621	0.21013
	**Analog 2**	CTCCAGCG	**Analog 2**	0.23431	0.05150	-0.06139	1.00000	-0.03748	0.26034	-0.07829
	**Analog 3**	GGCAACGG	**Analog 3**	0.36395	0.16018	-0.05230	-0.03748	1.00000	0.05001	0.25507
	**Analog 4**	AACCATGC	**Analog 4**	0.26829	0.29270	-0.05621	0.26034	0.05001	1.00000	-0.07814
	**Analog 5**	CGAAACTA	**Analog 5**	0.38340	0.22017	0.21013	-0.07829	0.25507	-0.07814	1.00000
**17**	**Origin**	GCGCGGAC	**Origin**	1.00000	0.57091	-0.01201	0.25948	-0.00763	0.43080	0.43303
	**Represent**	GCGCTACC	**Represent**	0.57091	1.00000	0.05561	0.36680	-0.05313	0.87106	0.47406
	**Analog 1**	GTACTGTC	**Analog 1**	-0.01201	0.05561	1.00000	0.29145	0.02241	0.06542	0.26465
	**Analog 2**	GTGCTTAT	**Analog 2**	0.25948	0.36680	0.29145	1.00000	-0.01774	0.40042	0.00083
	**Analog 3**	GGGTGTTC	**Analog 3**	-0.00763	-0.05313	0.02241	-0.01774	1.00000	-0.04833	-0.02859
	**Analog 4**	TCGCTACC	**Analog 4**	0.43080	0.87106	0.06542	0.40042	-0.04833	1.00000	0.28258
	**Analog 5**	GCGATGTA	**Analog 5**	0.43303	0.47406	0.26465	0.00083	-0.02859	0.28258	1.00000
**18**	**Origin**	TCGCCACA	**Origin**	1.00000	0.16170	0.44922	0.21657	0.64101	0.27899	0.20331
	**Represent**	CCCCCGGA	**Represent**	0.16170	1.00000	0.19004	0.24596	0.17887	0.21091	0.13406
	**Analog 1**	TCGCGGGG	**Analog 1**	0.44922	0.19004	1.00000	0.42061	0.41900	0.25550	-0.06622
	**Analog 2**	TTGCTGGA	**Analog 2**	0.21657	0.24596	0.42061	1.00000	0.23792	0.10102	0.04880
	**Analog 3**	ACGCCTTT	**Analog 3**	0.64101	0.17887	0.41900	0.23792	1.00000	0.26271	-0.06370
	**Analog 4**	CCGTAAGA	**Analog 4**	0.27899	0.21091	0.25550	0.10102	0.26271	1.00000	-0.09789
	**Analog 5**	AGCCTACA	**Analog 5**	0.20331	0.13406	-0.06622	0.04880	-0.06370	-0.09789	1.00000
**19**	**Origin**	TCATGAAT	**Origin**	1.00000	-0.00949	0.15826	0.23116	0.28224	0.36852	0.35182
	**Represent**	CAACGTGA	**Represent**	-0.00949	1.00000	0.46362	0.43065	0.16868	-0.07139	0.12788
	**Analog 1**	CTACGAGT	**Analog 1**	0.15826	0.46362	1.00000	0.03935	0.13492	-0.02120	0.11912
	**Analog 2**	TGATGTGA	**Analog 2**	0.23116	0.43065	0.03935	1.00000	-0.02862	0.03024	0.14160
	**Analog 3**	TCACATTT	**Analog 3**	0.28224	0.16868	0.13492	-0.02862	1.00000	0.28767	-0.00540
	**Analog 4**	ACATAATA	**Analog 4**	0.36852	-0.07139	-0.02120	0.03024	0.28767	1.00000	0.08360
	**Analog 5**	CATTGATT	**Analog 5**	0.35182	0.12788	0.11912	0.14160	-0.00540	0.08360	1.00000
**20**	**Origin**	ATCTGACC	**Origin**	1.00000	0.26964	0.30275	0.31792	-0.02161	0.32335	0.15209
	**Represent**	TGCCGACG	**Represent**	0.26964	1.00000	0.66261	0.19913	0.30973	0.12199	-0.00794
	**Analog 1**	TGCTTACG	**Analog 1**	0.30275	0.66261	1.00000	0.09222	-0.06380	0.15335	-0.04537
	**Analog 2**	GTTGGACT	**Analog 2**	0.31792	0.19913	0.09222	1.00000	-0.06929	0.15617	0.20825
	**Analog 3**	ACCCGGGC	**Analog 3**	-0.02161	0.30973	-0.06380	-0.06929	1.00000	-0.06889	0.10440
	**Analog 4**	CACGAACC	**Analog 4**	0.32335	0.12199	0.15335	0.15617	-0.06889	1.00000	0.17734
	**Analog 5**	AAGGGGCC	**Analog 5**	0.15209	-0.00794	-0.04537	0.20825	0.10440	0.17734	1.00000
**21**	**Origin**	ACCCGGTC	**Origin**	1.00000	0.33075	0.16127	0.20256	0.33173	-0.01255	0.10015
	**Represent**	GCCAGGGC	**Represent**	0.33075	1.00000	0.53582	0.39679	0.21022	0.35789	0.09944

	**Analog 1**	GCCGGAGC	**Analog 1**	0.16127	0.53582	1.00000	-0.01535	0.21414	0.17919	-0.01314
	**Analog 2**	TTCAGGAC	**Analog 2**	0.20256	0.39679	-0.01535	1.00000	0.03681	-0.00730	0.07655
	**Analog 3**	ACCTGTAT	**Analog 3**	0.33173	0.21022	0.21414	0.03681	1.00000	0.07166	0.32476
	**Analog 4**	AGCTTGGC	**Analog 4**	-0.01255	0.35789	0.17919	-0.00730	0.07166	1.00000	-0.12852
	**Analog 5**	ACTAGTCC	**Analog 5**	0.10015	0.09944	-0.01314	0.07655	0.32476	-0.12852	1.00000
**22**	**Origin**	ACAGCAGG	**Origin**	1.00000	0.47608	0.03917	0.54410	0.23557	0.17567	0.08931
	**Represent**	CCAGCGGC	**Represent**	0.47608	1.00000	-0.00500	0.47007	0.20716	0.48907	-0.05747
	**Analog 1**	GCCGGATG	**Analog 1**	0.03917	-0.00500	1.00000	0.04519	0.33333	-0.05592	-0.02680
	**Analog 2**	TCAGCTTT	**Analog 2**	0.54410	0.47007	0.04519	1.00000	-0.00631	0.00243	-0.05999
	**Analog 3**	GCCCCAGC	**Analog 3**	0.23557	0.20716	0.33333	-0.00631	1.00000	0.04722	0.46922
	**Analog 4**	CCGACGGG	**Analog 4**	0.17567	0.48907	-0.05592	0.00243	0.04722	1.00000	-0.06671
	**Analog 5**	AACCCAAG	**Analog 5**	0.08931	-0.05747	-0.02680	-0.05999	0.46922	-0.06671	1.00000
**23**	**Origin**	AGTTCGGG	**Origin**	1.00000	0.19543	-0.02009	0.44400	0.32394	0.55187	0.25505
	**Represent**	CCGACGTG	**Represent**	0.19543	1.00000	0.28223	0.47115	0.39046	0.43228	0.38670
	**Analog 1**	ATTACATG	**Analog 1**	-0.02009	0.28223	1.00000	0.22382	-0.12426	0.15805	0.12479
	**Analog 2**	ATGACGGA	**Analog 2**	0.44400	0.47115	0.22382	1.00000	0.20956	0.82471	0.21343
	**Analog 3**	CCGTAGGG	**Analog 3**	0.32394	0.39046	-0.12426	0.20956	1.00000	0.32964	-0.06698
	**Analog 4**	GTGACGGG	**Analog 4**	0.55187	0.43228	0.15805	0.82471	0.32964	1.00000	0.19179
	**Analog 5**	CGACCGTG	**Analog 5**	0.25505	0.38670	0.12479	0.21343	-0.06698	0.19179	1.00000
**24**	**Origin**	AGTTGTCT	**Origin**	1.00000	0.47914	0.23596	0.15301	-0.02788	0.20952	0.03895
	**Represent**	AGTGGTAT	**Represent**	0.47914	1.00000	-0.01428	0.15548	0.08936	0.34260	0.32121
	**Analog 1**	GCTTGGCA	**Analog 1**	0.23596	-0.01428	1.00000	-0.08417	0.08666	-0.02626	0.54016
	**Analog 2**	AGCTATAA	**Analog 2**	0.15301	0.15548	-0.08417	1.00000	-0.03620	-0.01600	-0.12064
	**Analog 3**	ACTCTTAT	**Analog 3**	-0.02788	0.08936	0.08666	-0.03620	1.00000	0.11128	0.19604
	**Analog 4**	CTTAGTAT	**Analog 4**	0.20952	0.34260	-0.02626	-0.01600	0.11128	1.00000	-0.01669
	**Analog 5**	ACTGGGCC	**Analog 5**	0.03895	0.32121	0.54016	-0.12064	0.19604	-0.01669	1.00000
**25**	**Origin**	ATGCACAT	**Origin**	1.00000	0.00479	0.21621	0.16952	0.31481	0.14309	0.23113
	**Represent**	GCGATCCA	**Represent**	0.00479	1.00000	0.06653	0.15373	-0.03656	0.54967	0.33392
	**Analog 1**	TTTCTCAC	**Analog 1**	0.21621	0.06653	1.00000	0.33687	0.18084	-0.06590	-0.01595
	**Analog 2**	AGAATCAT	**Analog 2**	0.16952	0.15373	0.33687	1.00000	0.18212	-0.03075	-0.00068
	**Analog 3**	GTCAACAG	**Analog 3**	0.31481	-0.03656	0.18084	0.18212	1.00000	0.16587	0.18624
	**Analog 4**	GCGAACGT	**Analog 4**	0.14309	0.54967	-0.06590	-0.03075	0.16587	1.00000	0.10858
	**Analog 5**	ATATACCA	**Analog 5**	0.23113	0.33392	-0.01595	-0.00068	0.18624	0.10858	1.00000
**26**	**Origin**	GTTTGGCG	**Origin**	1.00000	-0.02238	-0.02282	0.20800	0.31072	0.07872	0.11250
	**Represent**	TTGGTGTG	**Represent**	-0.02238	1.00000	0.47516	-0.03047	0.30487	0.11304	-0.08915
	**Analog 1**	GATATGTG	**Analog 1**	-0.02282	0.47516	1.00000	-0.03758	-0.05246	0.18928	-0.13021
	**Analog 2**	GTTCTTCT	**Analog 2**	0.20800	-0.03047	-0.03758	1.00000	0.07495	-0.02907	-0.02616
	**Analog 3**	GTGGCGCC	**Analog 3**	0.31072	0.30487	-0.05246	0.07495	1.00000	-0.06257	-0.09357

	**Analog 4**	ACTTTGAG	**Analog 4**	0.07872	0.11304	0.18928	-0.02907	-0.06257	1.00000	-0.08724
	**Analog 5**	CTCTGTGG	**Analog 5**	0.11250	-0.08915	-0.13021	-0.02616	-0.09357	-0.08724	1.00000
**27**	**Origin**	CTTTGTTG	**Origin**	1.00000	0.11236	0.18947	0.16954	-0.03439	0.21282	0.31688
	**Represent**	CACTATTT	**Represent**	0.11236	1.00000	0.19537	-0.08770	0.76537	0.13649	-0.01024
	**Analog 1**	CTCGTTTT	**Analog 1**	0.18947	0.19537	1.00000	0.03124	-0.01133	0.25858	0.26882
	**Analog 2**	CTTCAAAG	**Analog 2**	0.16954	-0.08770	0.03124	1.00000	0.09638	0.10037	0.08606
	**Analog 3**	CACTATAG	**Analog 3**	-0.03439	0.76537	-0.01133	0.09638	1.00000	0.00758	0.05329
	**Analog 4**	CTGGATTA	**Analog 4**	0.21282	0.13649	0.25858	0.10037	0.00758	1.00000	0.09226
	**Analog 5**	CTCAGTAT	**Analog 5**	0.31688	-0.01024	0.26882	0.08606	0.05329	0.09226	1.00000
**28**	**Origin**	GGAGGCTA	**Origin**	1.00000	0.10857	0.07013	0.37011	0.41986	0.11086	0.36267
	**Represent**	GCGGGGGC	**Represent**	0.10857	1.00000	0.13855	0.48153	0.34296	-0.07096	0.18274
	**Analog 1**	AGTAGGTA	**Analog 1**	0.07013	0.13855	1.00000	-0.06490	0.09421	-0.09398	0.16924
	**Analog 2**	CCGGGCGA	**Analog 2**	0.37011	0.48153	-0.06490	1.00000	0.17008	-0.01187	0.25898
	**Analog 3**	AGAGGAGC	**Analog 3**	0.41986	0.34296	0.09421	0.17008	1.00000	-0.06003	-0.05626
	**Analog 4**	GTCGACTT	**Analog 4**	0.11086	-0.07096	-0.09398	-0.01187	-0.06003	1.00000	0.06231
	**Analog 5**	GCTAGCTG	**Analog 5**	0.36267	0.18274	0.16924	0.25898	-0.05626	0.06231	1.00000
**29**	**Origin**	CGCATGTC	**Origin**	1.00000	0.57800	0.36864	0.24316	0.21531	0.14047	0.12375
	**Represent**	CGCGCGTC	**Represent**	0.57800	1.00000	0.07340	0.23250	0.39006	0.34517	0.21041
	**Analog 1**	GAAATGTA	**Analog 1**	0.36864	0.07340	1.00000	-0.09334	0.15945	0.17167	0.16539
	**Analog 2**	CGGGGCTC	**Analog 2**	0.24316	0.23250	-0.09334	1.00000	0.03329	-0.02774	-0.07036
	**Analog 3**	GGACCGTC	**Analog 3**	0.21531	0.39006	0.15945	0.03329	1.00000	0.06881	-0.07014
	**Analog 4**	CACGAGTG	**Analog 4**	0.14047	0.34517	0.17167	-0.02774	0.06881	1.00000	0.49229
	**Analog 5**	CACGTGGG	**Analog 5**	0.12375	0.21041	0.16539	-0.07036	-0.07014	0.49229	1.00000
**30**	**Origin**	ACTTGCCA	**Origin**	1.00000	0.21308	0.28402	0.23530	-0.02326	0.20339	0.09508
	**Represent**	GGGTGGCA	**Represent**	0.21308	1.00000	0.07028	0.69208	0.22750	0.09521	-0.01765
	**Analog 1**	TGTCCCCA	**Analog 1**	0.28402	0.07028	1.00000	0.08293	0.07897	0.15954	-0.01966
	**Analog 2**	GGGTGACA	**Analog 2**	0.23530	0.69208	0.08293	1.00000	-0.00105	0.10758	-0.01780
	**Analog 3**	AGTAGGAA	**Analog 3**	-0.02326	0.22750	0.07897	-0.00105	1.00000	-0.09332	-0.08751
	**Analog 4**	GATTCTCA	**Analog 4**	0.20339	0.09521	0.15954	0.10758	-0.09332	1.00000	0.33291
	**Analog 5**	ATTTTTCT	**Analog 5**	0.09508	-0.01765	-0.01966	-0.01780	-0.08751	0.33291	1.00000
